# Using Ambient Concentration Measurements to Quantify Volatile Organic Compound Emissions from Unconventional Oil and Gas Operations

**DOI:** 10.1021/acs.est.5c03994

**Published:** 2025-12-16

**Authors:** Weixin Zhang, Da Pan, I-Ting Ku, Yong Zhou, Jeffrey R. Pierce, Jeffrey L. Collett

**Affiliations:** † Department of Atmospheric Science, 3447 Colorado State University, Fort Collins, Colorado 80521, United States; ‡ School of Civil and Environmental Engineering, 1372 Georgia Institute of Technology, Atlanta, Georgia 30332, United States

**Keywords:** unconventional oil and gas development, VOC, emission rate, dispersion model, emission inversion, air quality

## Abstract

Oil and gas (O&G) development in the U.S. has accelerated in the past two decades, aided by unconventional extraction techniques. Potential environmental and health impacts of volatile organic compounds (VOCs) originating from O&G activities have raised concerns, but emission estimates remain highly uncertain. This study offers new insights into operation-specific VOC emission rates during unconventional O&G development (UOGD). We utilize dispersion model simulations with a new emission inversion method to analyze four years (2019–2022) of weekly air canister samples, measuring 48 VOCs at 10 monitoring sites in Broomfield, Colorado, where several large multiwell pads were drilled, completed, and entered production during the study period. Emissions are characterized for well drilling, hydraulic fracturing, coiled tubing/millout, flowback, and production operations. Drilling using synthetic drilling muds and coiled tubing operations exhibit the highest NMVOC emission rates, with median values of 2.8 g/s and 1.1 g/s, respectively. NMVOC and benzene emission rates during flowback were 96% and 98% lower, respectively, than previously reported values, highlighting the effectiveness of improved management practices in reducing air pollutant emissions from what used to be often the most significant emission source during UOGD. Our findings provide the first report of VOC emissions from coiled tubing/millout operations and show that the EPA’s nonpoint oil and gas emission estimation tool underestimates VOC emissions from drilling mud volatilization and flowback green completions.

## Graphical abstract

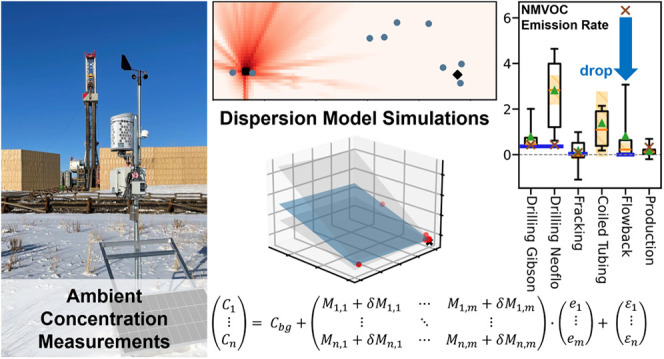

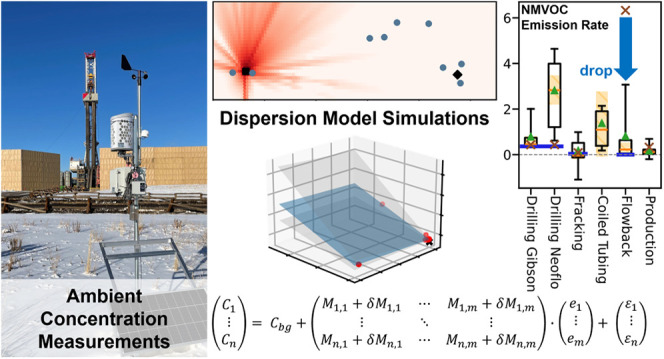

## Introduction

The United States became the world’s largest producer of oil and natural gas in 2014, a status enabled by significant advances in unconventional oil and gas (O&G) extraction techniques, most notably horizontal drilling and hydraulic fracturing.^1^ The rapid expansion of O&G activities has had significant environmental consequences. In basins across the country, from the Bakken in North Dakota to the Eagle Ford in Texas, emissions of volatile organic compounds (VOCs) from O&G operations have emerged as a pressing public health and environmental concern.^2–11^ These emissions often contain a complex mixture of compounds, including hazardous air pollutants such as benzene, toluene, ethylbenzene, and xylenes (BTEX), which are known to pose direct health risks.^12^ Beyond their immediate toxicity, VOCs are key precursors in atmospheric reactions that form secondary pollutants like ground-level ozone and fine particulate matter.^13–16^ The formation of ozone, in particular, is a persistent air quality challenge in many O&G production regions. Recognizing the scale of these emissions, the U.S. Environmental Protection Agency (EPA) has identified the O&G industry as the nation’s largest industrial source of VOCs, underscoring the critical need to accurately quantify and mitigate its atmospheric impact.^17^

A primary challenge in assessing this impact lies in the operational complexity of unconventional O&G development (UOGD). A modern multiwell pad is not a single, static source but rather a dynamic site that progresses, as it is developed, through multiple, distinct operational phases over weeks or months. The process begins with well drilling, where rigs—powered by diesel, natural gas, or increasingly, the electrical grid—use specialized hydrocarbon-based drilling muds to lubricate the drill bit and transport rock cuttings to the surface. This is followed by hydraulic fracturing (fracking), where a slurry of water, sand, and chemical additives is injected at high pressure into the target rock formation to propagate fractures, enhancing permeability. Subsequently, coiled tubing/millout operations are utilized to mill out the plugs that isolate different zones of the wellbore during fracking. The well then enters the flowback phase, where injected fluids and formation water, laden with dissolved hydrocarbons, return to the surface. Finally, once the flow of water subsides, the well transitions to its long-term production phase. Each of these stages involves different equipment, materials, and physical processes, creating the potential for a unique, operation-specific VOC emission profile (see Figure S1 for more details).

The existence of these distinct emission profiles has been demonstrated by source apportionment techniques like positive matrix factorization (PMF) using near-source concentration observations.^2,3,6,11,18^ For example, the volatilization of synthetic drilling muds can release a specific suite of heavy alkanes, while the operation of numerous diesel engines during fracking can be a significant source of combustion byproducts like ethyne. However, very few studies have successfully quantified speciated, operation-specific VOC emission factors. This lack of detailed data creates a significant knowledge gap, hindering the development of accurate emission inventories, which are critical for regional air quality modeling, human health exposure assessments, and the design of effective emission-control strategies.^5^

This knowledge gap persists due to limitations in both existing observation databases and conventional measurement methodologies. Existing inventories are based on EPA’s 2020 Emission Estimation Tool.^19^ However, these resources often rely on emission factors derived from decades-old observations that may not accurately reflect the practices, scale, and intensity of modern UOGD.^20^ For instance, the EPA tool does not provide emission factors for critical preproduction phases like coiled tubing/millout and assumes that certain modern practices, such as “green completions” during flowback, produce zero emissions. Field studies have directly contradicted this, revealing that even controlled operations can be significant sources. While foundational research by investigators like Hecobian et al.^5^ has provided invaluable direct measurements using tracer-ratio methods, these studies also highlight the challenges. Their work and following studies revealed significant variability between operational phases and demonstrated how rapidly evolving industry practices, such as the adoption of tankless, closed-loop systems during flowback, can dramatically reduce emissions compared to older techniques.^7,18^

Existing emission quantification methods also have inherent limitations for investigating UOGD emissions. Field-intensive techniques, including top-down mass balance methods with airborne data,^21–27^ mobile sampling methods with Gaussian-plume inversion,^28–32^ and tracer-ratio methods,^5,33–35^ typically provide only emission “snapshots” that can miss intermittent events and may not capture the full range of operational variability.^5,27,32,36^ Moreover, some of these methods require direct, on-site access to the well pad, which can be difficult to obtain and limits the feasibility of widespread, long-term monitoring campaigns. Conversely, complex Lagrangian models like Stochastic Time-Inverted Lagrangian Transport (STILT) and FLEXible PARTicle (FLEXPART), while suitable for long-term observations, are designed for regional-scale analysis. Their coarse grid resolution is insufficient for resolving emissions from a specific well pad using near-source observations (within 500 m).^37,38^ This highlights a clear and urgent need for a methodology that is capable of leveraging the increasing availability of continuous, long-term, near-source monitoring data to quantify emissions at the operational level.

To address the observation and methodology gaps, we introduce an innovative quantification method that leverages an extensive, multiyear ambient air monitoring data set. Our approach combines four years (2019–2022) of weekly VOC measurements with the AERMOD dispersion model through a multiple linear regression (MLR) emission inversion technique, supported by comprehensive uncertainty and quality control analyses. This method is specifically designed for the near-field, long-term analysis required to provide robust, time-integrated weekly emission rates that are more representative than snapshot measurements and better suited to resolving local sources than regional models. In this study, we apply this method to quantify emission rates for 48 individual VOCs across the full lifecycle of UOGD operations. By generating detailed emission rates for distinct preproduction phases, we aim to provide crucial insights into VOC emission dynamics during UOGD, improve the accuracy of emission inventories, and support the protection of public health in communities near O&G development.

## Materials and Methods

### Site Description and Ambient Air Monitoring

The City and County of Broomfield is located in the southwest portion of the Denver-Julesburg (DJ) Basin in northern Colorado. Six large multiwell O&G pads, consisting of 6–18 wells each, were developed at different times in Broomfield between 2019 and 2022 (Figure S2). The Broomfield Air Quality Monitoring Program (AQM), including 10 near-source, neighborhood, and regional VOC monitoring sites utilized here, was established to monitor the impacts of UOGD on local air quality (see Text S1 for more details).^7^ The locations of the six well pads and ten air monitoring sites are shown in Figure 1. The Commons (COM) site is located ∼ 5 km south of the well pads. Although the COM site was intended to serve as a regional benchmark away from local UOGD operations, it is sometimes influenced more heavily by local traffic emissions that lead to higher concentrations of some VOCs than near-pad sites (Figure S3). A meteorological station (39.9835° N, 105.0362° W) provided in situ wind measurements starting in April 2020.

**Figure 1:**
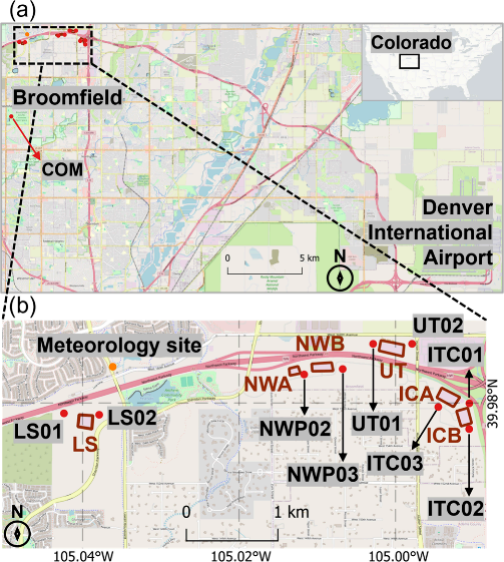
Study area and monitoring network. (a) Map of the Broomfield study area, showing Denver International Airport and the regional background monitoring site (COM). The inset shows Colorado’s location within the contiguous U.S. (b) Detailed view of Broomfield Air Quality Monitoring Network, showing the locations of the six large oil and gas well pads (shown as polygons, from left to right: Livingston (LS), Northwest A (NWA), Northwest B (NWB), United (UT), Interchange A (ICA), and Interchange B (ICB)), nine monitoring sites (shown as red dots, from left to right: LS01, LS02, NWP02, NWP03, UT01, UT02, ITC03, ITC01, and ITC02), and one meteorology site (providing in situ wind measurements, shown as orange dot).

Over the 192-week study period (April 2019 to December 2022, see Table S1), 1171 week-long time-integrated whole air canister samples (hereafter, weekly samples) were collected using evacuated 6.0L Silonite-coated stainless-steel canisters equipped with a flow controller (Entech Instruments Inc., Simi Valley, CA, USA) to regulate consistent sample collection over a 7-day period. Samples are collected as whole air samples without removal of ozone, water vapor, or other gases. Quality assurance and control protocols were followed, including canister cleaning and evacuation (see Text S2 for more details). Within 1–4 days, these air samples were analyzed using a five-channel Gas Chromatography (GC) at Colorado State University (CSU) for 48 VOCs and a Shimadzu GC-8A Flame Ionization Detector (FID) system for methane (CH_4_), providing long-term observations of VOCs from UOGD activities. Measurement uncertainties for alkanes are within 10% for most species, except for 2,2,4-trimethylpentane and alkylbenzenes (Table S2). Sample recovery tests confirmed that losses of hydrocarbons, including isoprene and ethene, from these canisters during typical weekly sampling and analysis schedules are not evident (within corresponding measurement uncertainties, see Table S3). This study utilizes weekly samples to quantify weekly emission rates from major operation phases, including drilling, fracking, coiled tubing/millout, flowback, and production. All operations lasted longer than 1 week for the well pads developed in Broomfield, and weekly canisters were collected from the 10 AQM monitoring sites over the same period (Figure S2).

### Dispersion Model and Meteorological Data

Dispersion models have been widely used to infer site-level emissions based on in situ measurements, a process known as inversion, where emission rates are estimated by minimizing the differences between predicted and observed concentrations. Previous studies differ mostly in their choices of dispersion models and inversion methods. Both the analytical Gaussian dispersion model and the AMS and EPA Regulatory Model (AERMOD)^39,40^ have been used to estimate CH_4_ emissions from landfills, O&G facilities, agricultural sources, and wastewater treatment facilities.^41–48^ The analytical Gaussian dispersion model has been used more frequently due to its lower requirements for terrain and upper atmospheric data, especially with mobile observations. However, Lan et al.^42^ compared the simple analytical Gaussian dispersion model with AERMOD and found that AERMOD simulations were more accurate for emission inversion.

AERMOD requires a comprehensive range of meteorological inputs, including vertical wind and temperature profiles.^49^ The AQM meteorological station only provided surface wind measurements at 5.6 m. The Denver International Airport weather station (DIA) operated by the National Weather Service provides vertical profiles but is located approximately 60 km away and had a data gap between July 9th and December 31st, 2022. Meteorological data generated using the Weather Research and Forecasting (WRF) – Advanced Research WRF (WRF-ARW, v4.2) model provide continuous and comprehensive coverage, with a horizontal grid resolution of 4 km × 4 km (Text S3 and Table S4). However, WRF-ARW wind directions have large discrepancies when compared with in situ wind measurements (Text S3 and Figures S4–S7). Given that no single set of data provides sufficient meteorological data for AERMOD simulations, we integrate WRF data and in situ observations to create a WRF-observation hybrid data set, which reduced discrepancies between predicted and observed concentrations and was used for the following analyses (Text S3 and Figure S8).

### Emission Inversion Method

To quantify weekly emissions, we developed a multiple linear regression (MLR) model where AERMOD-simulated dispersion coefficients (*M*_*i*,*j*_) serve as basis functions. These are scaled by the fitted emission rates (*e*_*j*_) to best match observed concentrations (*C*_*i*_) after accounting for a background concentration (*C*_*bg*_). The underlying equation, excluding fit residuals, applies the same linear assumption between the simulated dispersion pattern and ambient concentration as that used in multisource dispersion simulations for exposure assessment, which is the intended use case for the emission rates. We chose to infer *C*_*bg*_ instead of using observations from COM because some VOC concentrations observed at the COM site could at times be higher than those from near-pad AQM sites (Figure S3). The numbers of active monitoring sites (*n* ≤ 10) and operating sources (*m* ≤ 6) varied each week, and only weeks with *n* > m + 1 were used for inversion to ensure a nonzero degree of freedom (Figure S2). An example of the spatial layout of sources and monitoring sites, along with the corresponding AERMOD-simulated dispersion coefficients (*M*_*i*,*j*_), is shown in Figures 2a and 2b. The unknown parameters (*C*_*bg*_ and *e*_*j*_) were solved using orthogonal distance regression (ODR), a method chosen for its ability to consider fitting residuals in both the measured concentrations (ε_*i*_) and the AERMOD simulations (δ*M*_*i*,*j*_). The regression can be expressed as

1 $$\left(\begin{array}{c} C_{1}\\ \vdots \\ C_{n} \end{array}\right)=C_{bg}+\left(\begin{array}{ccc} M_{1,1}+\delta M_{1,1} & \cdots & M_{1,m}+\delta M_{1,m}\\ \vdots & \ddots & \vdots \\ M_{n,1}+\delta M_{n,1} & \cdots & M_{n,m}+\delta M_{n,m} \end{array}\right)\cdot \left(\begin{array}{c} e_{1}\\ \vdots \\ e_{m} \end{array}\right)+\left(\begin{array}{c} \varepsilon_{1}\\ \vdots \\ \varepsilon_{n} \end{array}\right)$$

**Figure 2:**
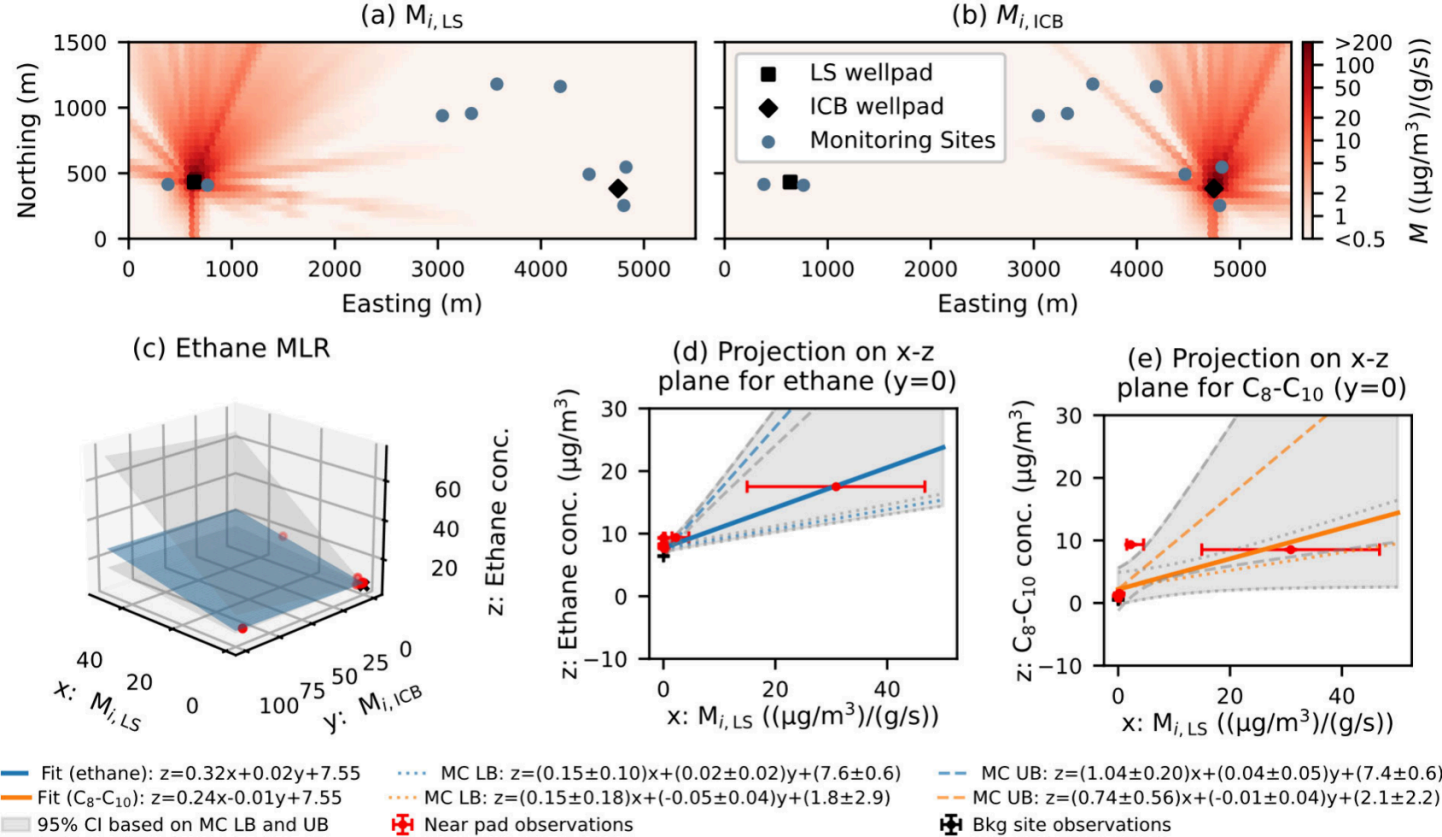
An example (July 11th, 2019) of the weekly multiple linear regression (MLR) method, where AERMOD-simulated dispersion coefficients for two sources (LS and ICB pads, a–b) are used to fit observed concentrations (markers). Panel (c) shows the resulting best-fit plane for ethane, and its slopes correspond to the estimated emission rates. Panels (d) and (e) visualize the fit for the drilling source (LS) for ethane (blue) and C_8_–C_10_ *n*-alkanes (orange), respectively, as two-dimensional projections. The solid lines are the best-fit, while the shaded gray areas represent the 95% confidence intervals (CIs). This interval is bounded by fits corresponding to the 97.5th percentile of the upper analytical bounds (UB, dashed lines) and the 2.5th percentile of the lower analytical bounds (LB, dotted lines) from the 2,000 Monte Carlo (MC) simulations. Red and black dots are observations from near-source and background sites. The horizontal and vertical bars denote simulation and observation uncertainties, respectively.

The ODR algorithm (Python SciPy.odr, version 1.13.1) minimizes a weighted sum of squared residuals (*W*) for both the measured concentrations and the model predictions:^50^

2 $$W=\overset{n}{\underset{i=1}{\sum }}\left(w_{\varepsilon_{i}}\varepsilon_{i}^{2}+\overset{m}{\underset{j=1}{\sum }}w_{\delta_{i,j}}{\delta M}_{i,j}^{2}\right)$$

where *w*_*ε*_*i*__ and *w*_δ_*i*,*j*__ are the weights for *C*_*i*_ and *M*_*i*,*j*_. *w*_*ε*_*i*__ are based on their uncertainties, as detailed in Table S2. For *w*_δ_*i*,*j*__, a standard weighting based on absolute model uncertainty (Δ*M*_*i*,*j*_) would give inappropriately large weight to background sites and bias the emission estimates because uncertainties from unmodeled local sources are not considered. To mitigate this bias, *w*_δ_*i*,*j*__ was calculated using relative uncertainty (*w*_δ_*i*,*j*__ = (*M*_*i*,*j*_/Δ*M*_*i*,*j*_)^2^), which focuses the analysis on data points where the signal from our target sources is strongest. The resulting fits using this relative uncertainty approach for the example are shown in Figures 2c–2e, while a comparison showing the biased results from using standard absolute uncertainty weights is provided in Figure S9.

A comprehensive uncertainty analysis was performed to combine input uncertainty (e.g., from wind fields and concentrations) and structural uncertainty (e.g., from AERMOD parametrizations and source configurations). We quantified input-related uncertainty using a 2,000-run Monte Carlo (MC) simulation with perturbed meteorological data and concentration observations (see Text S4 for more details). Δ*M*_*i*,*j*_ used for *w*_δ_*i*,*j*__ calculation was derived from the standard deviation of these MC simulations. We also conducted an additional 2,000-run MC simulation in which only the concentration observations were perturbed to investigate the impacts of VOC measurement precision. The structural uncertainty is inherently captured by the goodness-of-fit in the regression and is reflected as the analytical confidence intervals (CIs) of the MLR results. The final, conservative uncertainty was then defined by the 2.5th percentile of all lower bounds and the 97.5th percentile of all upper bounds. As illustrated in Figure 2, this method differentiates between input and structural errors, which vary by species. Ethane uncertainty is dominated by input error, as indicated by its wide confidence bounds, but C_8_–C_10_ *n*-alkanes show primarily structural uncertainty, with largely overlapping confidence intervals. This structural error suggests there are unmodeled sources of C_8_–C_10_ *n*-alkanes (such as drill cuttings stored at different locations within the pad), because the dispersion model accurately simulated ethane concentrations but not those of the C_8_–C_10_ *n*-alkanes.

Finally, the comprehensive uncertainty estimates serve as a robust metric for quality control. A large uncertainty in an emission estimate signifies a substantial discrepancy between simulated and observed concentration patterns or high model instability in response to input variations. Such cases indicate that the model cannot reliably constrain the emission rate. Therefore, we applied a statistical outlier test to remove results with extremely large uncertainties to ensure that our reported results are based only on the most robust and well-constrained emission estimates (Text S4). This procedure removed 24%, 40%, 14%, 46%, and 31% of NMVOC emission rates for drilling, fracking, coiled tubing, flowback, and production, respectively (see Figures S10–S17 and Table S5 for more details).

## Results

### Method and Uncertainty Evaluation

The performance of weekly AERMOD simulations using inversion-based emission rates is evaluated by comparing the predicted and observed VOC concentrations. The predictions are calculated using MLR inferred emission rates, and prediction 95% CIs are estimated using corresponding uncertainties of the emission rates (Text S4). Ku et al.^7^ and Lachenmayer et al.^18^ found that octane, nonane, and decane (C_8_–C_10_ *n*-alkanes) were unique tracers for drilling operations using synthetic Neoflo drilling mud (drilling with Neoflo) in Broomfield (Text S5) and that these compounds had very low concentrations in air not impacted by drilling with Neoflo. Neoflo mud was used at all pads except for the ICB well pad, which used a diesel-based Gibson drilling mud (hereafter referred to as drilling with Gibson). Therefore, we first quantify the emission rates of the sum of C_8_–C_10_ *n*-alkanes during drilling operations to assess AERMOD performance (Figure 3a). Predicted and observed C_8_–C_10_ *n*-alkanes show a good agreement with a regression slope (Ordinary Least Square (OLS)), a determination coefficient (R^2^), a root-mean-square error (RMSE), and a mean bias of 0.94 ± 0.01, 0.92, 0.92 μg/m^3^, and 0.02 μg/m^3^, as shown in Figure 3c. Moreover, our evaluation reveals that the MLR-inversion method appropriately assigns C_8_–C_10_ *n*-alkane emissions to well pad locations with active drilling operations and does not assign C_8_–C_10_ *n*-alkane emissions to other well pad locations (Figure 3a).

**Figure 3:**
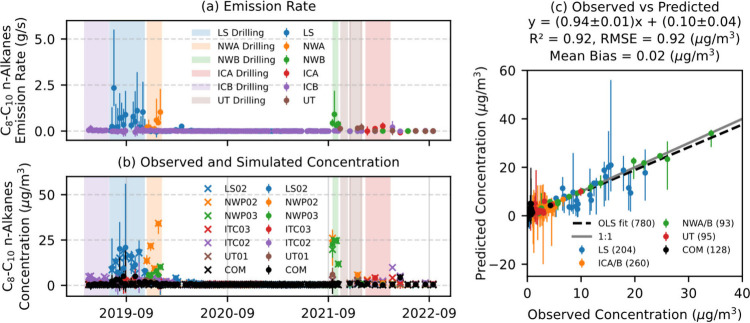
Time series of (a) derived C_8_–C_10_ *n*-alkane emission rates and (b) corresponding observed (crosses) and simulated (circles) concentrations at selected monitoring sites. In panel (a), different colors and markers represent unique well pads, while the shaded areas denote drilling periods of the well pads. Error bars in the panels (a) and (b) are uncertainties of constrained emission rates and predicted concentrations. Panel (c) shows a scatter plot of the final predicted and observed concentrations. The dashed black line indicates the ordinary least-squares (OLS) fit, and the solid gray line represents a 1:1 relationship.

Over the full 4-year observation period, the predicted and observed concentrations of ethane, benzene, and nonmethane total VOC (NMVOC), also show good agreement, with regression slopes ranging from 0.91 to 0.98, R^2^ ranging from 0.89 to 0.93, RMSE ranging from 0.08 to 11.11 μg/m^3^, and mean biases ranging from −0.0003 to 0.16 μg/m^3^ (Figure S18). These results demonstrate the reliability of using the MLR inversion method to obtain emission rates of other VOCs during UOGD operations.

The uncertainty in weekly emission rates varies significantly by operational phase and compound (see Figures S19–S24 and Text S4.8). A critical finding is that analytical precision is a minor component of the uncertainty in inferred emission rates. For all species, the overall uncertainty is dominated by other factors like atmospheric modeling, making the overall lower-bound uncertainty, on average, 15 to 70 times larger than the uncertainty from observation precision alone (see Figures S10–S13).

### VOC Emission Rates by UOGD Operation and Comparison with Previous Studies

Figure 4 presents UOGD operation-composited emission rates for key VOC species and groups (see Tables S6 and S7 for median and mean emission rates, respectively, for 48 VOCs during each UOGD operation). The reported uncertainties are aggregated 95% CIs for the median emission rates (for mean values in Table S7) specific to each operational phase. These were estimated using a hierarchical simulation that combines bootstrapping with Monte Carlo results to propagate all three sources of error: week-to-week sampling variability, input-related uncertainty, and structural uncertainty from the individual inversions (Text S4). This aggregation assumes that the true emission rates for a specific operation are consistent across different well pads and development times. Our 4-year trend analysis supports this assumption, as it found no significant systematic changes for most species (see Text S6 and Tables S8–S10). Except for coiled tubing, these aggregated uncertainties are considerably smaller than those for individual weekly estimates (Table S11), reflecting the increased statistical power gained from the larger sample size. Emission rates of coiled tubing have large variability and a small sample size, leading to high uncertainties. Comparing these emission rates from Broomfield with previously published values helps evaluate the current representativeness of prior data and the effectiveness of modern emission management practices. The EPA Emission tool^19^ is a particularly valuable comparison given its wide use by industry and government.

**Figure 4:**
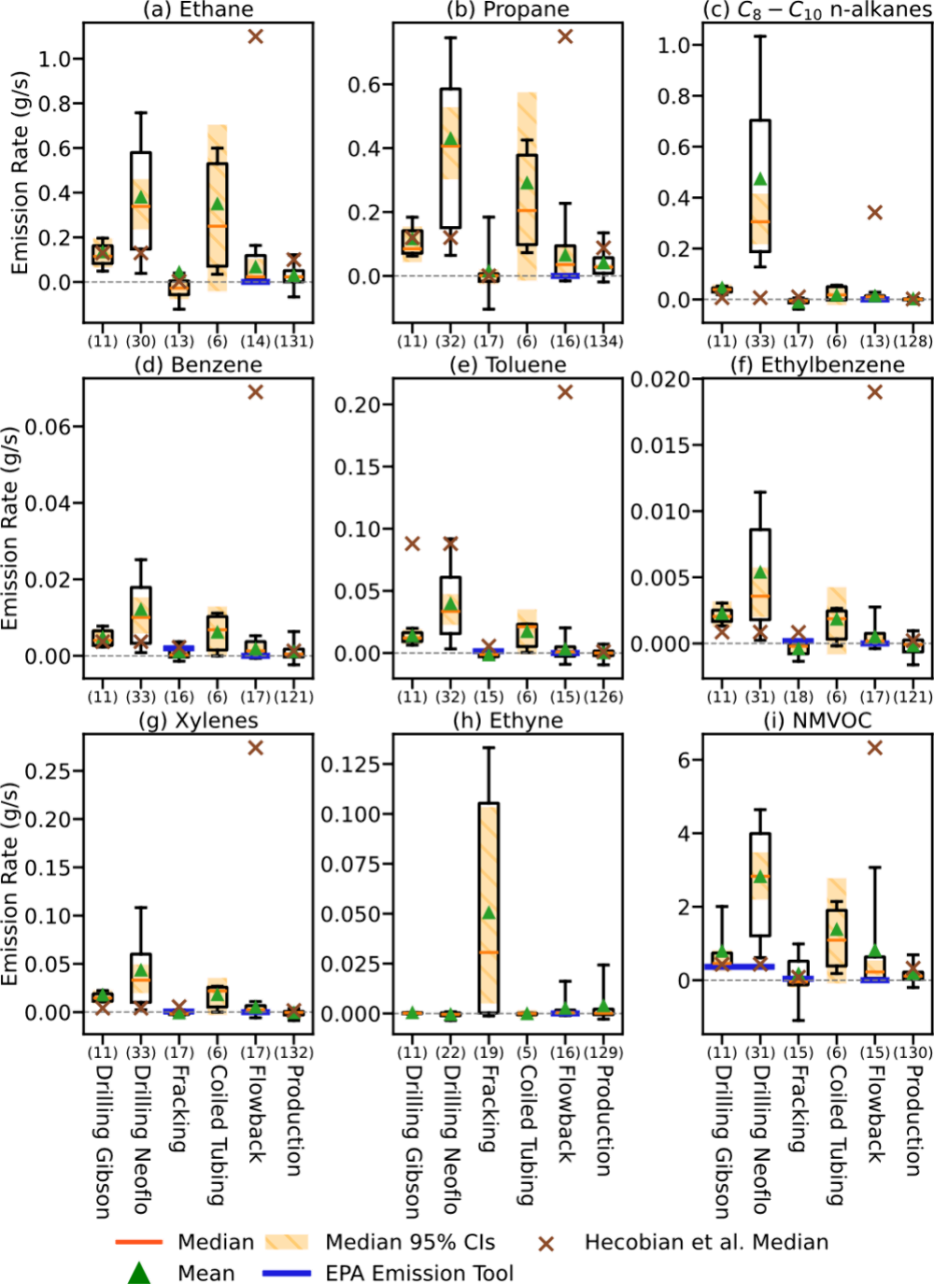
Emission rates of ethane, propane, C_8_–C_10_ *n*-alkanes, benzene, toluene, ethylbenzene, xylenes, ethyne, and nonmethane total VOC (NMVOC) during drilling with Gibson mud, drilling with Neoflo mud, fracking, coiled tubing/millout, flowback, and production operations. The boxes and whiskers represent the 5th, 25th, 75th, and 95th percentiles, respectively. The orange lines and green triangles represent the median and mean values, respectively. The orange shaded areas represent the aggregated 95% confidence intervals (CIs) for median emission rates as defined in Results. The blue lines represent emission factors listed in the EPA Emission Tool. The brown cross signs represent the median emission rates reported by Hecobian et al. (2019) from Colorado O&G operations between 2013 and 2016. The number of individual weekly average emission rates for each operation is listed in parentheses on the *x*-axis.

During drilling operations, the highest median emission rates of C_8_–C_10_ *n*-alkanes were observed when using Neoflo mud (0.30 [0.22, 0.42] g/s, values in brackets represent the lower- and upper-bound CIs), consistent with volatilization from this synthetic mud. Much smaller C_8_–C_10_ *n*-alkane emission rates were seen with Gibson mud (0.04 [0.02, 0.06] g/s). Median BTEX emission rates were also higher during drilling than in other operations. Compared to previous work (Table S12), median emission rates during drilling with Gibson mud in Broomfield of ethane, propane, benzene, and NMVOC are consistent with the median values reported by Hecobian et al. During drilling with Neoflo, median emission rates for most VOCs are higher than reported by Hecobian et al. Hecobian et al. did report a higher toluene emission rate (0.088 g/s) than Broomfield drilling with Neoflo (0.033 [0.023, 0.047] g/s), likely due to their study of conventional, fossil fuel-powered drill rigs, whereas the rigs in Broomfield were electrified. Overall, the NMVOC median emission rate for drilling with Neoflo mud (2.83 [2.20, 3.48] g/s) is higher than values provided in the EPA Emission Tool (0.36 g/s) and by Hecobian et al. (0.43 g/s). The drilling mud degassing emission factors in the EPA Emission Tool were based on a 1977 EPA report for offshore oil and gas development,^40^ yet the values are comparable to drilling with Gibson mud. However, NMVOC emissions from drilling with synthetic Neoflo-based mud in Broomfield are much larger, driven by increased emissions of heavier alkanes.

Fracking exhibited the lowest median emission rates for most alkanes and BTEX but the highest for the combustion tracer ethyne (0.031 [0.005, 0.103] g/s). This is expected, as fracking was powered by on-site diesel engines. For most other VOCs, emission rates have uncertainties larger than the median values, which are close to zero (Table S13), indicating that emissions are low enough that they do not raise local concentrations much above background levels. The NMVOC emission rates in the EPA Emission Tool for 1500 hp engines (a more realistic assumption for engine size^51^ compared to the default 700 hp option in the EPA Emission Tool) are small (0.08 g/s), and the NMVOC emissions reported by Hecobian et al. (0.08 g/s) are consistent with these values.

Coiled tubing/millout showed high median emission rates of ethane (0.25 [-0.04, 0.70] g/s), propane (0.2 [-0.01, 0.57] g/s), BTEX, and NMVOC (1.09 [-0.09, 2.77] g/s) (see Table S14 for more details). These compounds are naturally occurring, volatile constituents of the O&G within the reservoir, and the processes of milling out zone isolation plugs installed for fracking operations can lead to their venting to the atmosphere. This study provides the first report of VOC emissions from coiled tubing/millout operations, as neither Hecobian et al. nor the EPA Emission Tool provide emission rates for this operation. Significant uncertainties remain in the coiled tubing emission rates due to the limited sample size, and more observations are needed to further constrain these estimates. Finally, while shorter in duration than other preproduction operations, its elevated emissions reveal an opportunity for future emission reductions.

Historically, flowback has been identified as a UOGD period with high VOC emissions. Hecobian et al., for example, reported that even green completion flowback operations in the DJ Basin, with gas removal from flowback fluids using separators but on-pad tank storage of hot flowback fluids, still had large emissions (Table S15). Our emissions are significantly lower than those reported by Hecobian et al. For example, the median weekly average NMVOC emission rate of 0.23 [0.12, 0.54] g/s in Broomfield is just 3.6% of the median value reported by Hecobian et al. (6.33 g/s). This represents a 96% reduction in weekly average emissions, demonstrating the effectiveness of newer tankless, closed-loop systems used in Broomfield compared to the green completions studied a few years earlier.^52,53^ The difference is statistically significant, as even the upper-bound estimates for flowback in this study remain more than 90% lower than the median value reported by Hecobian et al. These results highlight a need to subcategorize green completions, a practice the EPA Tool assumes has a zero-emission rate, given their wide-ranging performance. It is important to note, however, that short-term, highly elevated emissions were still observed from Broomfield flowback operations, associated with periodic events like the emptying of sand canisters.^7^

During production, the median NMVOC emission rate (0.113 [0.085, 0.145] g/s) is lower than the 0.33 g/s reported by Hecobian et al. (Table S16). Lower emissions in Broomfield are not surprising, given the modern production facilities constructed in Broomfield compared to a mix of older and newer facilities included in the Hecobian et al. study.

## Discussion

In this study, we introduce a new quantification method that uses the MLR method to obtain VOC emission rates from UOGD activities based on weekly observations of ambient concentrations. Traditional approaches often provide either short-term “snapshots” that can miss operational variability or use regional-scale models with coarse resolution unsuitable for resolving emissions from specific well pads. Our approach bridges this gap by pairing the AERMOD dispersion model with a MLR inversion technique specifically designed for long-term, near-source analysis. We also systematically quantified the impact of input-related errors combining results from a 2,000-run MC simulation and analytical errors of the MLR methods. We found that the primary source of uncertainty in the derived weekly emission rates stems not from the analytical uncertainty of the VOC concentration measurements, but from the inherent structural and input-related limitations of the AERMOD dispersion model.

The use of week-long integrated samples aligns the measurement time scale with the operational time scale for large, multiwell pads. The major operational phases investigated in this study were not short-term events but rather continuous processes that often lasted for weeks or months. By averaging over a full week, the method effectively smooths out the subhourly meteorological fluctuations and plume meander that AERMOD cannot resolve, yielding a more stable, robust, and defensible estimate of the weekly emission rate for a given operation. However, the operation phases defined here also have suboperations lasting minutes, hours, or days with varying emission rates. For example, drilling operations include several phases for each well, including rig setup/movement, vertical drilling, horizontal drilling through the hydrocarbon “pay zone,” pipe tripping (pulling of pipe out of the wellbore), and cementing/casing, and each of these activities can have distinct emission rates. Closed-loop, tankless flowback operations require periodic emptying of sand from canisters used to trap frack sand emerging from the wellbore. Changing emissions across such short duration suboperations, however, are not discernible using weeklong observations. Using shorter time scale observations, such as triggered canisters lasting for a few minutes, remains challenging, because of dispersion modeling artifacts that compromise short-term analyses as discussed in previous work.^54,55^ This warrants future research in improving model capability for accurate dispersion estimates on hourly or shorter time scales.

VOC emission rates can vary significantly across different operations. Drilling and coiled tubing exhibit higher VOC emission rates as hydrocarbons, including alkanes and aromatics, are released from subsurface deposits and, in the case of drilling, recycled drilling mud and drill cuttings. Conversely, fracking emissions were generally lowest as material is primarily being pushed downhole. However, fracking’s reliance on powerful diesel engines produced the highest emissions of the combustion tracer ethyne. These engines are also significant NO_*x*_ sources, an important ozone precursor. While drilling operations in the DJ Basin are increasingly being electrified, the larger power requirements of fracking operations presently limit electrification as an emission reduction strategy. In contrast to earlier studies by Hecobian et al.,^5^ we found average flowback emissions to be greatly reduced. Reductions of 96% and 98% in emissions of NMVOC and benzene, respectively, demonstrate the efficacy of modern tankless, closed-loop systems over the green completions with on-pad flowback fluid storage in vented tanks studied by Hecobian et al. The success of this strategy highlights a crucial opportunity for mitigating air quality impacts. Wider adoption of these improved management practices is especially valuable in populated, ozone-sensitive, or disproportionately impacted communities.

Although median VOC emission rates during the production phase are much lower than those seen during many preproduction operations, the extended duration of production means that emissions continue over years to decades, causing cumulative emissions to climb over time.^56,57^ Increased VOC emissions have also been observed during production site maintenance operations, including separator cleaning.^7^

VOC preproduction and production emissions may also vary substantially across other O&G producing regions. Local requirements on emissions management can vary substantially, different operators and subcontractors often employ varying operational practices, and even an individual operator might use different practices across locations. For example, the use of grid-powered, electrified drill rigs; closed-loop, tankless fluid handling systems; and synthetic drilling mud in Broomfield are not common practices at every location and differences in these practices can significantly alter emissions. Moreover, different O&G reservoirs have distinct oil and gas compositions, which may also affect the composition of emissions from a well pad.^5^

The findings presented here fill a crucial knowledge gap regarding VOC emission rates during different UOGD operations. Accurate knowledge of these emission rates, across a range of industry practices that are evolving over time, can be used to properly assess health risks associated with air toxics exposure for individuals living, working, or playing near O&G operations and to improve our understanding of how these operations contribute to the formation of ozone and regional haze.

## Supplementary Material



## Notes

Six supplementary texts covering Broomfield O&G well pad development timelines and air quality monitoring program, whole air canister sample collection and VOC analysis, meteorological data for AERMOD simulations, emission inversion method with uncertainty and performance analysis, drilling tracers, and trend analysis; 24 supplementary figures and 16 supplementary tables detailing model inputs, quality control, model evaluation, and complete emission rate results (PDF)
