# Supplementary material for: Using Ambient Concentration Measurements to Quantify Volatile Organic Compound Emissions from Unconventional Oil and Gas Operations
Source: Environ Sci Technol. 2025 Dec 16;59(51):27881–90. doi: 10.1021/acs.est.5c03994 (PMC12756904; doi:10.1021/acs.est.5c03994)
Supplement: Supplementary file 1 [file es5c03994_si_001.pdf]

Supporting Information for

Using Ambient Concentration Measurements to Quantify Volatile Organic Compound Emissions from Unconventional Oil and Gas Operations

Weixin Zhang[1,2], Da Pan[1,2,*], I-Ting Ku[1], Yong Zhou[1], Jeffrey R. Pierce[1], and Jeffrey L. Collett, Jr. [1,*]

[1]Department of Atmospheric Science, Colorado State University, Fort Collins, Colorado, 80521, United States

[2]School of Civil and Environmental Engineering, Georgia Institute of Technology, Atlanta, Georgia, 30332, United States

[*]Corresponding authors:

Jeffrey L. Collett, Jr. (collett@colostate.edu)

Da Pan (da.pan@ce.gatech.edu)

Summary of content:

Number of pages	73
Number of texts	6
Number of figures	24
Number of tables	16

Supplementary Texts

Supplementary Text 1. Broomfield O&G Well Pads Development Timelines and Air Quality Monitoring Program

In Broomfield, Colorado, six O&G well pads were developed between 2019 and 2022. The pre-production development timelines of each O&G well pad are shown in Figure S2 panel (a) including drilling, hydraulic fracturing, coiled tubing/millout, production tubing installation and flowback. After flowback, production begins. For the six O&G well pads in Broomfield, the pre-production developments included here spanned 192 weeks (from 2019-04-18 to 2022-12-29).

Based on O&G well pads development schedules, the Air Quality Monitoring program began in October 2018, prior to the start of development of the first well pad and contained multiple monitoring sites (details can be found in Ku et al. (2024)). Some of these sites were set up near the well pads and one was set at a background/reference location several kilometers from the new UOGD activities. Weekly whole air canister samples (weekly samples) collected at these sites were scheduled based on the operational timelines of nearby O&G well pads. Therefore, the sampling schedules varied from site to site. Table S1 lists the number of weekly samples collected at the 10 monitoring sites in Broomfield between the listed start date and end date. Figure S2 panel (b) depicts the sampling timeline for each monitoring site in Broomfield. Samples were collected at COM, the background/reference site, continuously active throughout the whole period except for two weeks. Weekly samples at other monitoring sites were not always continuous because the quantity of active monitoring sites for each week varied in accordance with operations at nearby O&G well pads.

Supplementary Text 2. Whole Air Canister Sample Collection and VOC Analysis

This section, adapted from Weber (2018), details the protocols for gas sample collection, chemical analysis, and quality assurance and control (QA/QC) for the measurement of 48 volatile organic compounds (VOCs).

2.1 Sample Collection

Weekly time-integrated whole air samples were collected using Entech Instrument 6.0L stainless steel canisters with an internal Silonite® coating. This internal surface allows for stable storage of VOCs for over one month (LeBouf et al., 2012). An Entech CS1200ES Flow Controller was attached to each canister to maintain a constant flow rate over the 7-day collection period. Samples were analyzed within 1 to 4 days of collection and always within 30 days.

To address concerns about potential reactions between ozone and reactive species like alkenes during the week-long sampling integration, a stability test was performed. Ambient air containing 56 ppbv of ozone was collected in the canisters and analyzed over seven days. The results, summarized in Table S3 below, showed no significant loss of key alkenes. The most reactive species of interest, isoprene and ethene, had recoveries of 95.8% and 99.8%, respectively. Their difference from 100% are within measurement uncertainty. This suggests that losses of hydrocarbons during the sampling and analysis schedule are likely insignificant, possibly due to the loss of ozone to the inert canister walls.

2.2 Sample Analysis

VOC concentrations are analyzed using a custom-built 5-channel gas chromatography (GC) system equipped with three Flame Ionization Detectors (FID), an Electron Capture Detector (ECD), and a quadrupole mass spectrometer (MS). Prior to analysis, a 540 cm^3 (STP) aliquot of each sample was cryogenically pre-concentrated to effectively trap light VOCs. The analytical

procedures followed the guidelines of EPA Compendium Method TO-15. A complete analysis cycle for a single sample took approximately 33 minutes. Measurement precisions are provided in Table S2.

2.3 Quality Assurance and Quality Control (QA/QC)

Canister Cleaning: Before deployment, canisters were cleaned and evacuated using an automated Entech 3100D Canister Cleaning System. The process involved eight cycles of evacuating the canister and refilling it with Ultra High Purity Nitrogen at 80 degree C to remove residual VOCs. For each cleaning batch, one canister was randomly selected as a batch blank and analyzed to verify cleaning efficacy.

Instrument Performance and Uncertainty: The GC system's baseline and carryover were checked by analyzing VOC-free air. Instrument drift was monitored by analyzing a Linde Gas North America LLC high-pressure standard gas at the end of each analysis day. The relative standard deviation (RSD) of the 5-channel GC system was determined to be 1–8% for non-methane hydrocarbons, 3–15% for halocarbons, and 3–8% for alkylnitrates. RSDs are combined with uncertainties of standard gas composition to estimate measurement uncertainties for all reported species, which are provided in Table S2.

Detection Limits: A nine-point calibration was performed for all 48 target VOCs before sample analysis. The limit of detection (LOD) for each compound was calculated from the analysis of VOC-free air blanks, defined as the mean blank signal plus three times its standard deviation, divided by the calibration slope. For any measurement below the LOD, a value of one-half the LOD was used.

Supplementary Text 3. Meteorological Data for AERMOD Simulations

3.1 Weather Research and Forecasting (WRF) Configuration

The hourly meteorological data used for the AERMOD simulations were generated using the Weather Research and Forecasting (WRF) model. The detailed configuration of the WRF model and its processing are provided below.

The model used was the WRF-ARW (Advanced Research WRF) core, version 4.2. The model was initialized using data from the National Centers for Environmental Prediction (NCEP) Global Forecast System (GFS) at 0.5-degree resolution.

The WRF simulation employed a nested grid approach, with the innermost domain having a horizontal grid resolution of 4 km×4 km, from the data for the study site were extracted. The model was configured with 35 vertical ETA pressure levels, and the map projection was Lambert Conformal Conic. Land use characterization was based on the MODIS 21 land use category data. For each 24-hour simulation period, a 6-hour spin-up time was used for model initialization, and these initial 6 hours were discarded from the final output. The specific physics option selected for the WRF simulation are detailed in Table S4.

Following the simulation, the WRF output was processed for use in AERMET using the US EPA's Mesoscale Model Interface Program (MMIF). Data were extracted from the center of the grid cell in the innermost domain that was closest to the study area's specific latitude and longitude. The processing with MMIF followed the recommendations outlined in the US EPA guidance document, "Guidance on the use of the mesoscale model interface program (MMIF) for AERMOD applications." The specific parameters for our study location (Broomfield, CO) are: center point (39.97173° N, 105.0407° W), datum (WGS 84), UTM zone (13), base elevation (1604.91 m),

WRF grid cell (4 km×4 km), site time zone (UTC-0700), closest city & country (Broomfield, USA).

3.2 Wind Data Comparison

EPA recommends using meteorological data from National Weather Service (NWS) stations. The closest station to the Broomfield area is at Denver International Airport (DIA). The DIA station provides hourly surface and twice daily upper air meteorological data. However, the study areas are approximately 60 km away from DIA. Considering the influence of complex terrain that is much closer to Broomfield than to DIA and that the local wind field can change dramatically during a short period of time, the meteorological data from DIA station might not be representative for our study area. As an alternative to NWS station data, EPA also suggests using the prognostic meteorological data generated by the WRF model. We obtained both meteorological data sets (DIA & WRF), with the WRF data provided by Lakes Environmental Consultants Inc. We found that even with the supplementary Automated Surface Observing Systems (ASOS) wind data prepared by AERMINUTE, there are still some missing values in the DIA data set. For example, as of September 2025, the DIA upper air sounding data after July 9, 2022, are not published. Using the WRF meteorological data can avoid problems with such missing values. Moreover, the airport only provides twice-daily upper air sounding data which is extrapolated into hourly upper air data by AERMET while the WRF data provides a higher resolution for the upper air meteorology.

In-situ hourly winds collected at the Meteorology site (orange dot in Figure 1, 39.9835° N, 105.0362° W) are compared with WRF and DIA wind data for the period from January to June 2022. The site is close to all the monitoring sites (<5 km). The in-situ wind data were measured at a height of 5.6 meters above ground, whereas WRF and DIA wind data were reported at 10 meters. Therefore, we adjusted the in-situ wind speed (WS) to 10 meters using Equation S1 (Stull, 1988),

where z is the height of the wind (z_2 = 10 m and z_1 = 5.6 m); u is the WS at the given height; d is the displacement height; and z_0 is the surface roughness length obtained from the WRF meteorological data, ranging from 0.1–0.12 m. The log wind profile is valid for neutral conditions. We did not include the corrections for non-neutral conditions to avoid introducing additional uncertainties. Given that the measurement site is in a grassland, d << z_1 and z_2 and is ignored.

$$u(z_2) = u(z_1) \cdot \frac{\ln\left(\frac{z_2 - d}{z_0}\right)}{\ln\left(\frac{z_1 - d}{z_0}\right)} \quad \text{(S1)}$$

Figure S4 presents the hourly averaged wind direction (WD) and WS from WRF and DIA compared with in-situ measurements, while Figure S5 shows daily averaged wind comparison. Wind directions (left panels in Figures S4 and S5) are colored by WS. Wind speeds (right panels in Figures S4 and S5) are compared with in-situ measurements using orthogonal distance regression (ODR). The comparisons include mean bias, mean error, normalized mean bias (NMB), and root mean square error (RMSE) of both WRF and DIA wind data.

Analysis of Figures S4 and S5 reveals that DIA WD observations are more consistent with in-situ WD measurements than those of WRF. When low WS values (< 2 m/s) are excluded, the majority of DIA WD data points fall near the 1:1 line in relation to in-situ WD, while WRF WD data show greater scattering (Figures S6 and S7). Furthermore, DIA hourly WD observations exhibit higher fractions of WD differences that are smaller than 15 and 30 degrees (25% and 46%) compared to WRF (17% and 30%), reflecting the limitations in WRF's ability to simulate WD accurately. Conversely, the ODR slope between WRF WS and in-situ WS is closer to 1 than the slope between DIA WS and in-situ WS. WRF WS values show lower mean bias, mean error, RMSE, and NMB than DIA WS measurements, indicating that DIA wind measurements likely underestimate WS.

3.3 Impacts of Wind Direction on AERMOD Simulations

With the WRF dataset as inputs, AERMOD simulations miss several plumes observed at LS01 site and substantially underestimate C_8–C_{10} n-alkanes (Figure S8 left panel). In contrast, the WRF-observation hybrid dataset has lower WD errors and significantly improves the performance of AERMOD simulations (Figure S8 right panel). For example, with the hybrid dataset, the regression slope, R^2 value, and overall RMSE between predicted and observed concentrations are 0.92, 0.87, and 2.24 μg/m3, respectively. When using WRF dataset alone, these values are 0.75, 0.69, and 3.51 μg/m3, respectively. Therefore, simulations with the hybrid dataset and corresponding constrained emission rates are reported in this study.

Supplementary Text 4. Emission Inversion Method

4.1 Multiple Linear Regression Method with Orthogonal Distance Regression

To infer weekly emission rates, this study employs a multiple linear regression (MLR) framework (Eq. 1 in the main text), where AERMOD-simulated dispersion coefficients serve as the basis functions. The core of this inversion is the statistical method used to solve unknown parameters: the emission rates (e_j) and the background concentration (C_{bg}). A critical methodological decision was the use of Orthogonal Distance Regression (ODR) instead of the more common Ordinary Least Squares (OLS) regression. The OLS method operates on the fundamental assumption that the independent variables (in this case, the AERMOD-simulated dispersion coefficients, $M_{i,j}$ are known without error). Any discrepancy between the model and observations is attributed solely to random error in the dependent variable (the measured concentrations, C_i). However, this assumption is invalid in this application. The AERMOD dispersion coefficients are themselves model outputs, subject to significant uncertainty from errors

in their meteorological inputs (e.g., wind speed and direction) and from the physical parameterizations of the model.

The ODR method provides a more statistically rigorous and physically defensible alternative by acknowledging that errors exist in both independent and dependent variables (Brown and Fuller, 1990). The ODR algorithm does not simply minimize the vertical distance (residuals in C_i) between the data points and the regression hyperplane. Instead, it minimizes the weighted sum of the squared orthogonal distances from each data point to the regression hyperplane (Eq. 2 in the main text). The algorithm's objective is to find the set of parameters ($\hat{e}_j$ and $\hat{C}_{bg}$) that defines a best-fit hyperplane by simultaneously adjusting for errors in both the measured concentrations (ε_i) and the AERMOD simulations ($\delta M_{i,j}$). The use of ODR is thus a foundational choice that reflects a sophisticated understanding of model-data fusion, leading to more reliable parameter estimates and, crucially, more realistic uncertainty bounds that properly account for model fallibility.

4.2 Derivation of the Analytical 95% Confidence Interval

For each weekly inversion, an analytical 95% confidence interval (CI) is calculated for each fit parameter. This CI represents the structural uncertainty for that specific inversion, quantifying the range of parameter values consistent with the data, given the model structure and a single realization of the inputs (Refsgaard et al., 2006). The entire ODR analysis was conducted using the scipy.odr package (version 1.13.1) in Python, and the CI calculation follows standard statistical theory based on the outputs of this specific software package (Brown and Fuller, 1990). The computational steps are as follows:

1. **Calculation of Standard Errors**: The standard error (SE) for each fitted parameter is the square root of its estimated variance. The package provides an output, sd_beta, which contains the standard errors for each parameter.

2. **Construction of the Confidence Interval**: The 95% confidence interval for each parameter is constructed using its estimated value, its standard error, and a critical value $t(0.975, \mathrm{df})$ from the Student's t-distribution. The use of the t-distribution, rather than the normal (Z) distribution, is statistically appropriate for regressions with a small number of data points, as is the case here where the number of active monitoring sites (<10) is limited. The formula for the 95% CI of the j-th parameter is:

$$\mathrm{CI}_{95\%}(\hat{\beta}_j) = \hat{\beta}_j \pm t(0.975, \mathrm{df}) \cdot \mathrm{SE}(\hat{\beta}_j(\mathrm{S2})$$

where:

- $\hat{\beta}_j$ is the best-fit estimate for the parameter (the emission rate (e_j) or background concentration (C_{bg})) returned by the ODR algorithm.
- $\mathrm{SE}(\hat{\beta}_j)$ is the standard error of that parameter, taken directly from the sd_beta array in the scipy.odr.Output object.
- df represents the degrees of freedom for the regression, calculated as $n - (m + 1)$, where n is the number of active monitoring sites and m is the number of active well pads for that week.

This analytical CI provides a robust measure of the structural uncertainty for a single inversion. It quantifies how well the AERMOD-based model structure can constrain the emission rates based on the provided observations, under the temporary assumption that the model inputs are perfectly known. The following section details the methodology used to relax this assumption and incorporate the effects of input uncertainty.

4.3 Quantifying Input Uncertainty via Monte Carlo Simulation

The analytical confidence interval derived from a single ODR fit, while informative, only captures structural uncertainty. It does not account for the significant uncertainty associated with

the inputs to the inversion process. To address this, a comprehensive uncertainty analysis was performed using a 2,000-run Monte Carlo (MC) simulation, which systematically propagates the known uncertainties from meteorological data and measured VOC concentrations through the entire modeling and inversion chain to the final emission rate estimates (Kroese et al., 2006). The framework involves creating a large ensemble of 2,000 plausible input datasets. Each dataset is generated by perturbing the baseline inputs with random error terms drawn from their respective, empirically-derived uncertainty distributions. The full ODR-based inversion is then performed independently for each of the 2,000 perturbed datasets. This process yields a distribution of outcomes that directly reflects the impact of input uncertainty on the derived emission rates. This approach explicitly separates input uncertainty from structural uncertainty: the variability of results across the 2,000 MC runs is driven by input uncertainty, while the width of the confidence interval calculated within each individual run is driven by structural uncertainty.

Uncertainty in meteorological data, particularly wind speed and direction, is a dominant source of error in atmospheric dispersion modeling. The four-year study period (2019–2022) involved a significant change in the source and quality of the available meteorological data, necessitating a time-dependent perturbation scheme. For each of the 2,000 MC iterations, and for each hour of the weekly integration period, the baseline wind speed and direction values were perturbed by adding a random error term. This error was drawn from a normal distribution with the following protocol:

- Period 1 (Prior to April 4, 2020): During this initial phase of the study, local, on-site meteorological measurements were not available. Wind direction data were sourced from the Denver International Airport (DIA) weather station, located approximately 60 km from the study area. The significant distance and potential for differing local wind patterns introduce substantial uncertainty into these data. Wind speed data were taken

from the Weather Research and Forecasting (WRF) model, which also has known uncertainties. To reflect this higher uncertainty, a random error was drawn from a normal distribution defined by a 95% CI of **±30 degrees for wind direction** and **±3 m/s for wind speed**.

- Period 2 (From April 4, 2020, onwards): After this date, in-situ wind measurements became available. Because of this improved data quality, the magnitude of the perturbation was reduced. For this period, a random error was drawn from a normal distribution defined by a 95% CI of **±9 degrees for wind direction** and **±1.5 m/s for wind speed**, which were three times higher than the measurement precisions (3 degrees and 0.5 m/s) to reflect the variability in regional wind directions and speed.

This explicit, time-dependent treatment of meteorological uncertainty is a critical feature of the methodology, demonstrating that the analysis was tailored to the known quality of the input data throughout the study period. Furthermore, this MC process serves a dual purpose. Beyond propagating uncertainty, it also informs the ODR weighting itself. The standard deviation of the 2,000 AERMOD-simulated dispersion coefficients for each source-receptor pair ($M_{i,j}$), resulting from the 2,000 perturbed meteorological scenarios, provides a direct, simulation-based estimate of the model uncertainty, ($\delta M_{i,j}$). This value is then used to calculate the relative uncertainty weights ($w_{\delta_{i,j}} = \left(M_{i,j}/\Delta M_{i,j}\right)^2$) used in the ODR algorithm. This creates a sophisticated and internally consistent feedback loop where the MC simulation not only quantifies the final uncertainty but also improves the central estimate of each ODR fit by providing physically-based regression weights.

In addition to meteorological data, the measured VOC concentrations (C_i) are also subject to analytical uncertainty. To account for this, the observed concentrations were perturbed in each of the 2,000 MC iterations. For each weekly sample at each monitoring site, a random error term was added to the measured concentration. This error was drawn from a normal distribution with a mean of zero and a standard deviation equal to the species-specific measurement uncertainty. These uncertainty values are based on laboratory quality assurance protocols and are detailed for each VOC species in Table S2.

4.4 Synthesis of Structural and Input Uncertainties

The final 95% CI combines the structural uncertainty captured by the ODR and the input uncertainty quantified by the MC simulation. From the collection of 2,000 lower bounds generated from the MC simulation, the 2.5th percentile value was selected to serve as the final lower bound of the reported CI. Similarly, from the collection of 2,000 upper bounds, the 97.5th percentile value was selected to serve as the final upper bound. This approach is considered "conservative" because it constructs an interval designed to contain the true emission rate with at least 95% probability by considering the full range of outcomes from both structural and input errors. The resulting interval is wider than one that might be derived from simply taking the 2.5th and 97.5th percentiles of the 2,000 central emission rate estimates. This method explicitly accounts for scenarios where a specific combination of perturbed inputs might lead to a particularly poor model fit (i.e., a very wide analytical CI), ensuring that this "worst-case" structural uncertainty is reflected in the final reported range.

4.5 An Example of Emission Inversion on 2019-07-11

The emission inversion and uncertainty synthesis method are illustrated with an example using observations from July 11, 2019, as noted in the main text. On this date, two well pads had active

operations and were emitting VOCs. All nine near-pad monitoring sites and the background site (COM) provided weekly samples.

The results using relative model uncertainties as weights are shown in Figure 2 in the main text, while the results using absolute model uncertainties as weights are shown in Figure S9. It is important to note that a Monte Carlo (MC) simulation was not performed using these absolute uncertainty weights; therefore, the 95% CIs in Fig. S9 are the analytical CIs from the ODR method. Compared to the fit that used relative uncertainties, the fit based on absolute AERMOD errors is more sensitive to variability in observations away from the active well pads, which could be impacted by unmodeled emissions. For this reason, we chose to use relative AERMOD errors as weights for the ODR analysis.

The power of the uncertainty synthesis method can be illustrated by considering the different uncertainty characteristics of ethane and C_8–C_{10} n-alkanes. For a species like ethane, which is well-modeled by AERMOD, the structural uncertainty is relatively small. Each of the 2,000 analytical CIs would be narrow. However, the emission estimate is sensitive to meteorological inputs. Therefore, the central values of these narrow CIs would shift significantly with each MC run, leading to a widespread distribution of both lower and upper bounds. The final percentile-based method captures this input-dominated uncertainty. For species like C_8–C_{10} n-alkanes, which were found to have significant unmodeled sources (e.g, cuttings with residual drilling muds and hydrocarbons stored at a different location on the pad), the structural uncertainty is large. Each of the 2,000 analytical CIs would be inherently very wide, reflecting the model's difficulty in fitting the observations. Even if the central estimates do not shift dramatically between MC runs, the sheer width of each CI ensures that the distributions of lower and upper bounds are very broad. The final synthesis method correctly captures the large total uncertainty in both cases, whereas a

simpler method (e.g., one based only on the distribution of central estimates) would fail to capture the full extent of the structural error for the C_8–C_{10} n-alkanes. This comprehensive approach provides a final uncertainty estimate that is not only robust and conservative but also diagnostic of the underlying sources of error in the inversion.

4.6 Quality Control Using Uncertainty Estimates

The comprehensive uncertainty estimates, which synthesize both structural and input-related errors, serve as a robust metric for quality control of the weekly emission inversions. A large uncertainty in a final emission estimate is a key diagnostic indicator that the inversion for that week was not well-constrained. This can arise from two primary issues: (1) a substantial discrepancy between the spatial patterns of simulated and observed concentrations, leading to a poor model fit and high structural uncertainty, or (2) high model instability, where small perturbations in meteorological or concentration inputs lead to large swings in the calculated emission rates, indicating high sensitivity to input uncertainty. In either scenario, a large final uncertainty signifies that the model cannot reliably constrain the emission rate from the available data. Therefore, results with extremely large uncertainties must be identified and removed to ensure the integrity of the final aggregated emission statistics.

A multi-step, data-driven approach was applied to systematically identify and remove these unreliable emission estimates. The weekly emission estimates were first grouped by operational stage (e.g., drilling, fracking, production) and by chemical species. This step was necessary because input-related uncertainties often scale with the magnitude of the emission rates, which can vary by orders of magnitude between different operations and compounds. Grouping ensures that the uncertainty of a given estimate is compared to a distribution of uncertainties from physically similar scenarios, preventing the erroneous flagging of high-but-appropriate uncertainties from

high-emission phases. For each group, a statistical outlier test based on the interquartile range (IQR) was applied to the distributions of the associated uncertainties. This test was performed independently for the lower-bound and upper-bound uncertainties. An emission estimate was flagged as a statistical outlier and removed if its lower- or upper-bound uncertainty exceeded the 75th percentile of its group plus three times the group's IQR for that uncertainty bound.

Figures S10–S13 and S14–S17 show the quality-controlled and raw emission rates for different operation phases. This combined, data-driven approach removes outliers and ensures that the reported results are based only on the most robust and well-constrained emission estimates. The results that were removed through this quality control process were predominantly from the production phase (see Table S5). This outcome is expected, as the typically low-magnitude emissions during the production phase are more difficult for the model to distinguish from background concentrations and measurement noise, leading to less stable inversions and, consequently, higher relative uncertainties.

4.7 Model Performance Evaluation Using Predicted Concentrations

As discussed in the main text, the performance of weekly AERMOD simulations using inversion-based emission rates is evaluated by comparing the predicted and observed VOC concentrations. The predictions are calculated using MLR inferred emission rates, and prediction 95% CIs are estimated by propagating the uncertainty from the fitted parameters ($\hat{\beta}$) to the predicted concentration ($\hat{C}_{pred}$). This is achieved using a first-order Taylor series expansion, also known as the delta method, which is a standard statistical technique for approximating the variance of a function of random variables (ISO., I. and BIPM OIML, 1993).

The procedure is as follows:

1. **Define the Prediction Model**: The predicted concentration at a given site i is a linear function of the fitted parameters ($\hat{\beta} = [\hat{C}_{bg}, \hat{e}_1, ...,\hat{e}_m]$) and the vector of dispersion coefficients for that site $M_i = [1, M_{i,1}, ..., M_{i,m}]$:

$$\hat{C}_{pred,i} = f(\hat{\beta},M_i) = \hat{C}_{bg} + \sum_{j=1}^{m} M_{i,j} \cdot \hat{e}_j = M_i \cdot \hat{\beta}^T (S3)$$

2. **Obtain Parameter Covariance Matrix**: From the ODR output for a given fit, we obtain the variance-covariance matrix of the fitted parameters, $\text{Cov}(\hat{\beta})$. This is calculated as the product of the "cov_beta" matrix and the residual variance "res_var" from the scipy.odr output. This matrix contains the variances of each parameter on its diagonal and the covariances between pairs of parameters on its off-diagonals.

3. **Calculate the Jacobian**: The Jacobian of the prediction function with respect to the parameters, J, describes the sensitivity of the output to changes in each parameter. For this linear model, the Jacobian is simply the vector of dispersion coefficients:

$$J = \frac{\partial f(\hat{\beta},M_i)}{\partial \hat{\beta}} = [1,M_{i,1},..., M_{i,m}] (S4)$$

4. **Propagate Uncertainty**: The variance of the predicted mean concentration, $\text{Var}(\hat{C}_{pred,i})$, is calculated using the matrix formula for propagation of uncertainty:

$$\text{Var}(\hat{C}_{pred,i}) = J \cdot \text{Cov}(\hat{\beta}) \cdot J^T (S5)$$

5. **Calculate Standard Error of the Fit**: The standard error of the predicted mean concentration (also known as the standard error of the fit) is the square root of its variance:

$$\text{SE}(\hat{C}_{pred,\,i}) = \sqrt{\text{Var}(\hat{C}_{pred,i})} (S6)$$

6. **Construct the Confidence Band**: The 95% confidence band is then constructed around the predicted value using a t-multiplier, consistent with the parameter CI calculation:

$$\mathrm{CI}_{95\%}\left(\hat{C}_{pred,\,i}\right) = \hat{C}_{pred,\,i} \pm t(0.975, \mathrm{df}) \cdot \mathrm{SE}\left(\hat{C}_{pred,\,i}\right) (S7)$$

The degrees of freedom, df, are the same as those used for the parameter confidence intervals ($n-(m+1)$).

This confidence band calculation is performed for the specific ODR fits that define the final, conservative uncertainty bounds for the emission rates. Specifically, the confidence band shown in plots such as Figure 2 of the main manuscript is derived from the parameter estimates and covariance matrix of the ODR run that corresponds to the 97.5th percentile of all upper bounds and the run that corresponds to the 2.5th percentile of all lower bounds. This ensures that the uncertainty in the model's predictions is fully consistent with the comprehensive and conservative uncertainty quantification framework used for the emission rates themselves.

4.8 Uncertainty for Weekly Emission Rates

The uncertainty in weekly emission rates varies significantly by operational phase and compound (see Fig. S21–S26). Emissions from drilling and coiled tubing/millout operations have high fractions of individual weekly emission rates significantly different from zero for compounds like ethane (67%), C_8–C_{10} n-alkanes (73% and 50%), benzene (57% and 67%), and total NMVOC (79% and 67%, respectively). In contrast, the 95% CIs of individual weekly emission rate estimates during fracking, flowback, and production often overlap with zero. During fracking, for instance, the fraction of significant non-zero measurements drops to just 13% for both NMVOC and benzene, and to zero for C_8–C_{10} n-alkanes. Notably, ethyne, a combustion tracer, is the only species with a high fraction of significant rates during this phase (53%).

4.9 Aggregated Uncertainty for Median Emission Rates

We report the median VOC emission rates as the typical values for each UOGD operation. To aggregate uncertainties for these median values, we used a hierarchical approach that propagates

all three identified sources of uncertainty, week-to-week sampling variability, input uncertainty, and structural uncertainty, into the final aggregated median estimate (Cohen et al., 1996).

This was accomplished through a nested sampling procedure. First, a bootstrap sample of weeks was created by randomly selecting n weeks with replacement from all weeks within a given operational phase. Second, for each week in the bootstrap sample, a single emission rate was generated through a two-stage draw. This draw involved (a) randomly selecting one of the 2,000 Monte Carlo runs for that week to account for input uncertainty, which provided a parameter estimate (β_k) and its standard error (SE_k), and then (b) drawing a single random value from a Student's t-distribution centered at β_k with a scale of SE_k to account for the structural uncertainty of that specific ODR fit. Finally, the median of the resulting n emission rate values was calculated.

This entire process was repeated 10,000 times to generate a distribution of 10,000 median values. The final 95% confidence interval for the aggregated median was then determined by the 2.5th and 97.5th percentiles of this distribution. This method was used to generate the uncertainties for the median values shown in Fig. 4 and Tables S6 and S11–S16.

Supplementary Text 5. Drilling Tracers

Ku et al. (2024) found that the use of a synthetic drilling mud in Broomfield caused significantly elevated concentrations of C_8–C_{10} n-alkanes (n-octane, n-nonane, and n-decane) near O&G well pads. To alleviate odor complaints, the operators switched from a petroleum hydrocarbon distillate-based drilling mud (Gibson D822) to a synthetic paraffin Neoflo 4633-based drilling mud (Neoflo 4633) after developing the first well in Livingston (LS) pad on 2019-07-11 (CDPHE, 2020). Concentrations of C_8–C_{10} n-alkanes observed near the well pad using the Neoflo 4633 drilling mud (Livingston (LS) pad) were significantly higher than those observed near the prior well pad drilled using the Gibson D822 drilling mud (Interchange B (ICB) pad), as detailed in Fig.

5 of Ku et al. (2024). Headspace analyses of the two drilling muds were conducted in the lab at the Department of Atmospheric Science, Colorado State University (see Fig. S4 in Ku et al., 2024). The results show that the Neoflo 4633 drilling mud emits more n-octane, n-nonane, and n-decane than the Gibson D822 drilling mud by a factor of about 10, consistent with the enhanced concentrations of these species observed during drilling operations at the LS vs. the ICB pads. As discussed by Ku et al. (2024), the C_8–C_{10} n-alkanes are unique tracers for drilling operations using the Neoflo 4633 mud because emissions of C_8–C_{10} n-alkanes from the drilling mud are far higher than from other sources.

Supplementary Text 6 Trend Analysis

The long-term observations in this study present a unique opportunity to investigate temporal trends in UOGD emission rates. A one-year interruption in well development due to the COVID-19 pandemic (see Fig. 3a) provides a distinct before-and-after period for assessing temporal changes in emissions from pre-production activities. To compare emission rates before and after the operational gap, we used the Mann-Whitney U-test (Sheskin 2003). This non-parametric statistical test was chosen because it compares the median emission rate between two periods and is robust against the influence of high-emission outlier events, which are common in UOGD operations. A significance level of $p < 0.05$ was used for all tests. Our analysis was not performed for drilling with Gibson, fracking, or coiled tubing. Gibson muds were not used after July 2019. Coiled tubing and fracking were excluded because the limited number of samples after 2019 ($n<=4$) was insufficient for a reliable statistical analysis.

For drilling with Neoflo, the median emission rates for most species showed no significant change. However, the U-test revealed a statistically significant increase for a suite of aromatic compounds (e.g., propylbenzene, ethyltoluenes, trimethylbenzenes, and xylenes). For flowback,

the analysis showed a significant decrease in emissions for n-hexane, 2-methylheptane, octane, m/p-xylene, and the sum of C_8–C_{10} n-alkanes were lower after the interruption. Although the magnitudes of these trends are statistically significant, they are often in the same order as the measurement uncertainties. For example, the median decrease for the lumped C_8–C_{10} n-alkanes during drilling with Neoflo was -10.9 mg/s, which is only slightly larger than the typical lower-bound uncertainty of 8.8 mg/s. Detailed trend and uncertainty analyses are provided in Tables S8 and 9.

For the quasi-continuous production phase emissions at the ICB and LS well pads, we used the Mann-Kendall test to identify monotonic trends (Hirsch et al., 1982). Ethyne showed a statistically significant decreasing and increasing trends at the ICB and LS well pads, respectively. These opposite trends highlight the variability of production emission rates due to the difference in the age, type, and maintenance schedule of the equipment. For the majority of the NMVOC species measured, no statistically significant trends were observed at either well pad (see Table S10 for more details).

Our 4-year trend analysis indicates a general absence of widespread, systematic trends for most species across the various operational phases. Although a few statistically significant changes were detected, their magnitudes were often comparable to the measurement uncertainties, suggesting they do not reflect a broad evolution in emissions during the study period. Similarly, for the long-term production phase, a Mann-Kendall test revealed no statistically significant monotonic trends for the vast majority of NMVOC species at either of the continuously monitored well pads. The conflicting trends observed for ethyne at different sites further underscore that the primary driver of emission variability is likely site-specific factors rather than a systematic evolution over time. The absence of systematic trends is not surprising given that all the Broomfield well pads studied

here were developed by the same O&G operator, under the same operator with local government and state permitting action.

Supplementary Figures

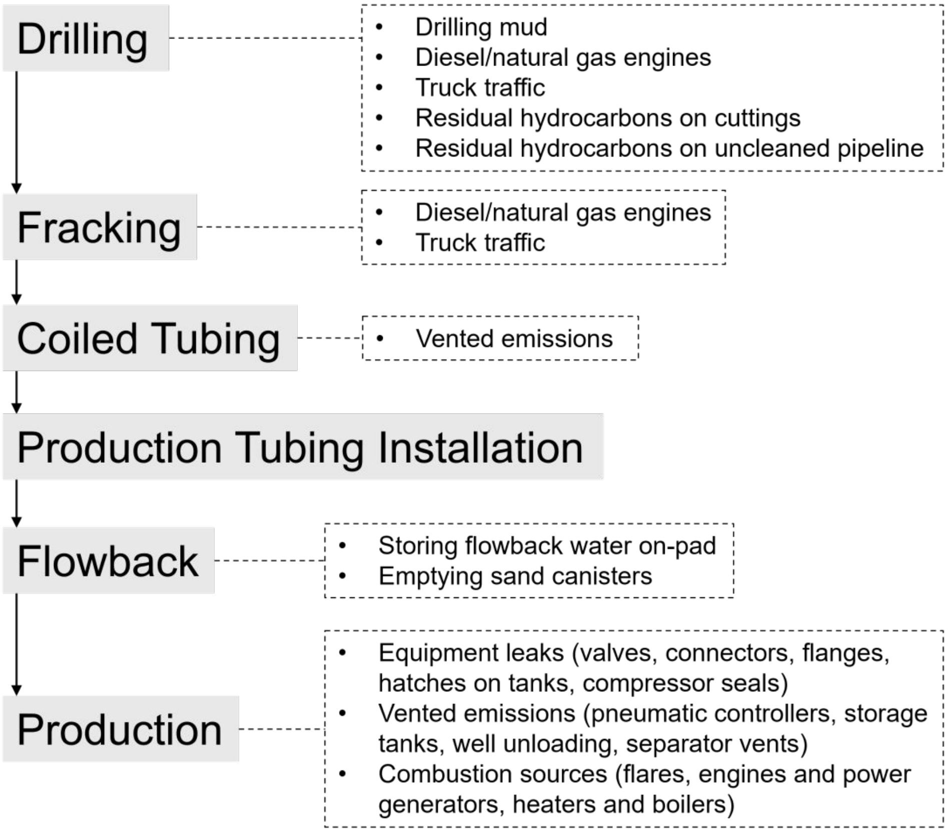

Figure S1. A schematic of unconventional oil and gas development workflow, comprising the key phases of drilling, fracking (hydraulic fracturing), coiled tubing/millout, production tubing installation, flowback, and production. Potential emission sources throughout the process are highlighted in dashed-line boxes.

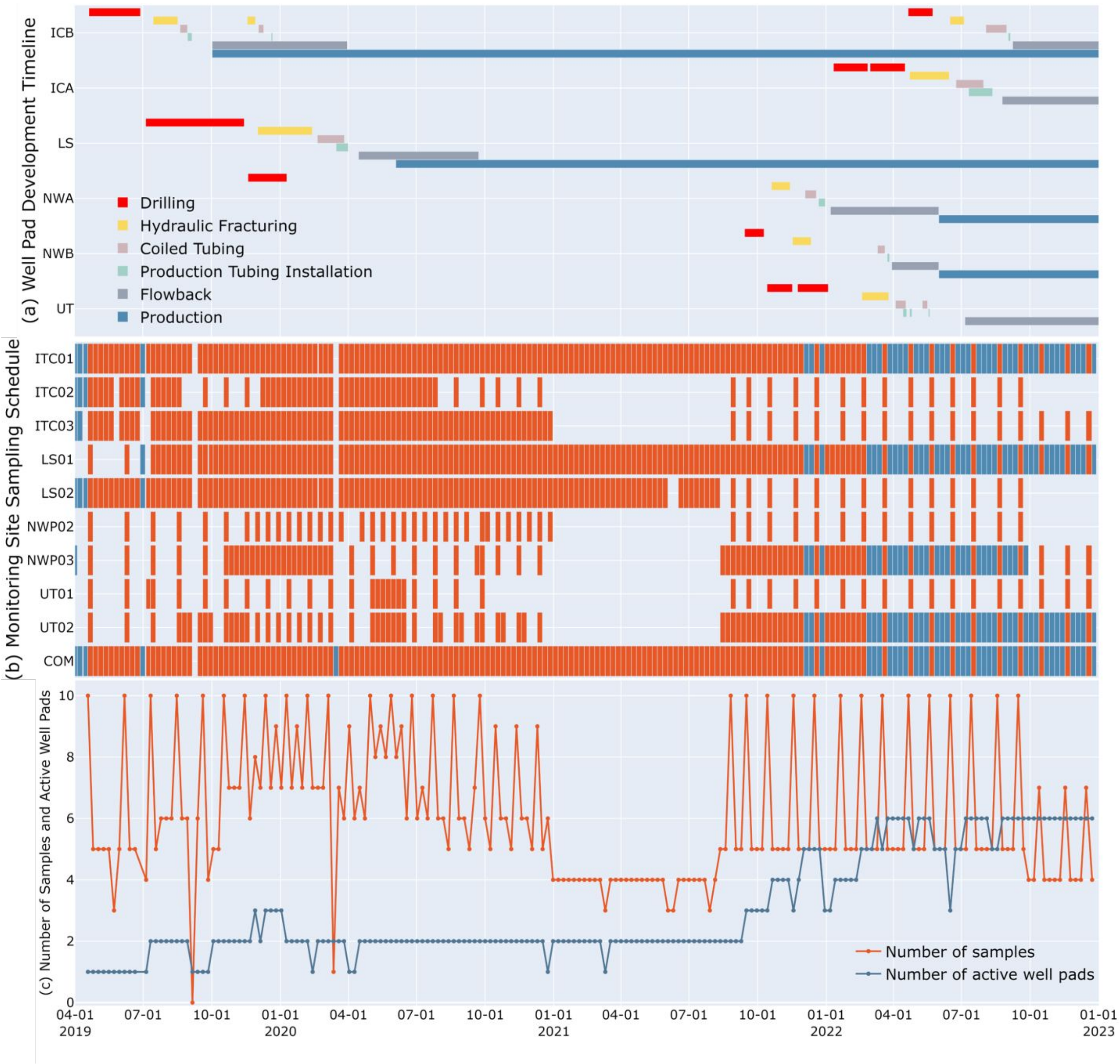

Figure S2. Panel (a) shows the development timelines for the six oil and gas well pads in Broomfield between 2019 and 2022. Six different operations were categorized in different colors here. Panel (b) shows the sampling schedules for the ten monitoring sites between 2019 and 2022. In panel (b), time periods in orange are the weeks we had enough samples to constrain the emission rates of active well pads while time periods in blue are the weeks excluded in the emission

inversion analysis due to the small number of observations. Panel (c) shows the number of active well pads (value of m in Equation 1) and number of samples collected (value of n in Equation 1).

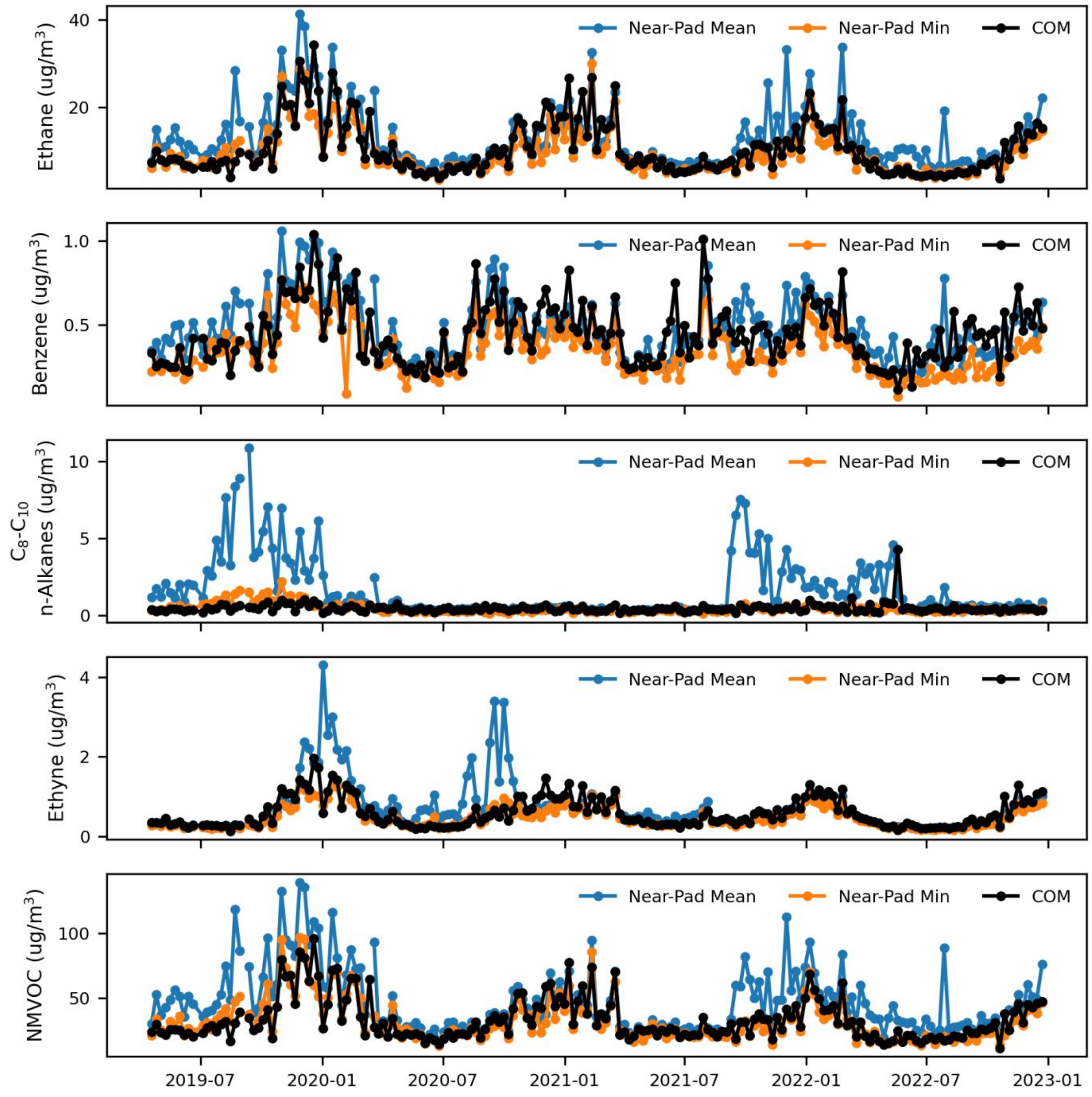

Figure S3. Comparison between observations of ethane, benzene, C_8–C_{10} n-alkanes, ethyne, and total NMVOC made at the background site (COM; black) and the mean (blue) and minimum

(orange) values from the sites near the well pads. Background observations can be higher than near-pad observations, especially for ethane, benzene, and ethyne.

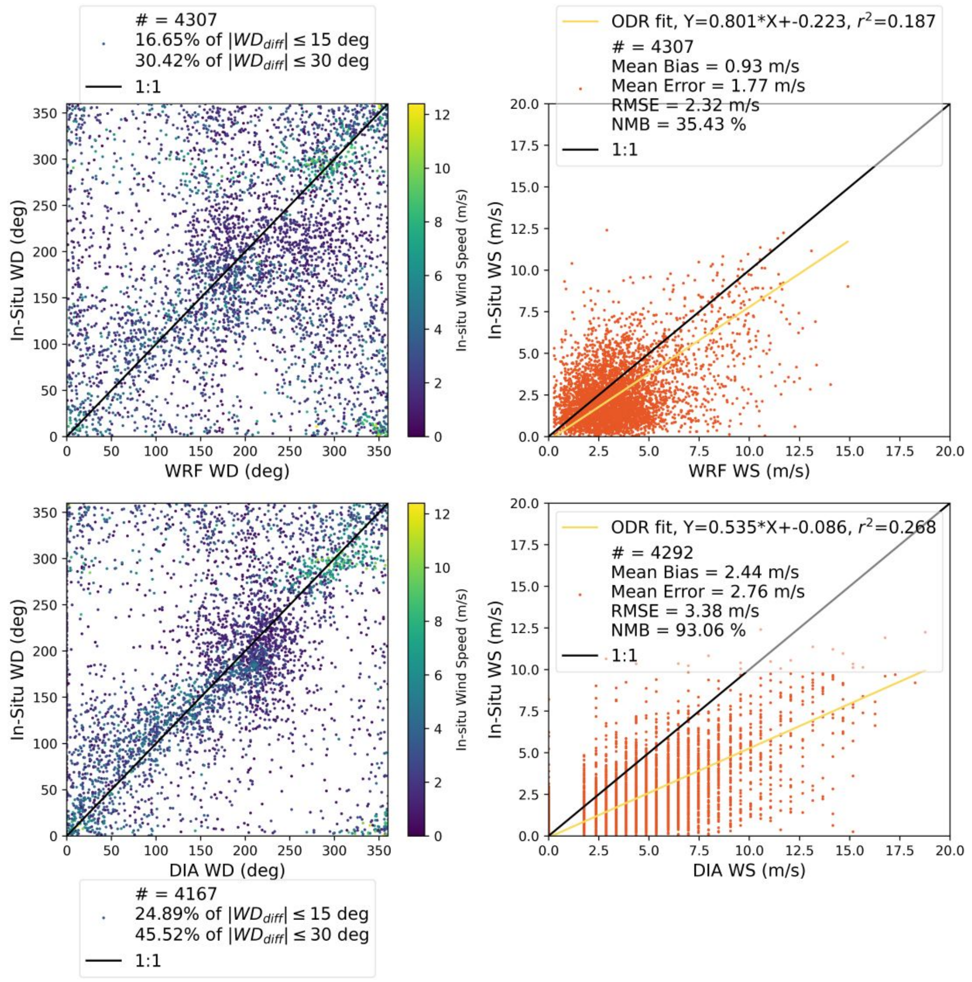

Figure S4. Hourly wind direction (WD) and wind speed (WS) comparison between in-situ observations and WRF (top panels) & Denver International Airport (DIA) (bottom panels) from January to June 2022. For wind direction, scatter plots are colored by wind speed. Fractions of absolute wind direction differences $|WD_{diff}| \leq 15$ deg and 30 deg are included, respectively. For wind speed, statistical metrics are provided: mean bias, mean error, root mean square error (RMSE), and normalized mean bias (NMB). All in-situ wind speeds were corrected to the same altitude using Equation S1, as described in SI Text 3.

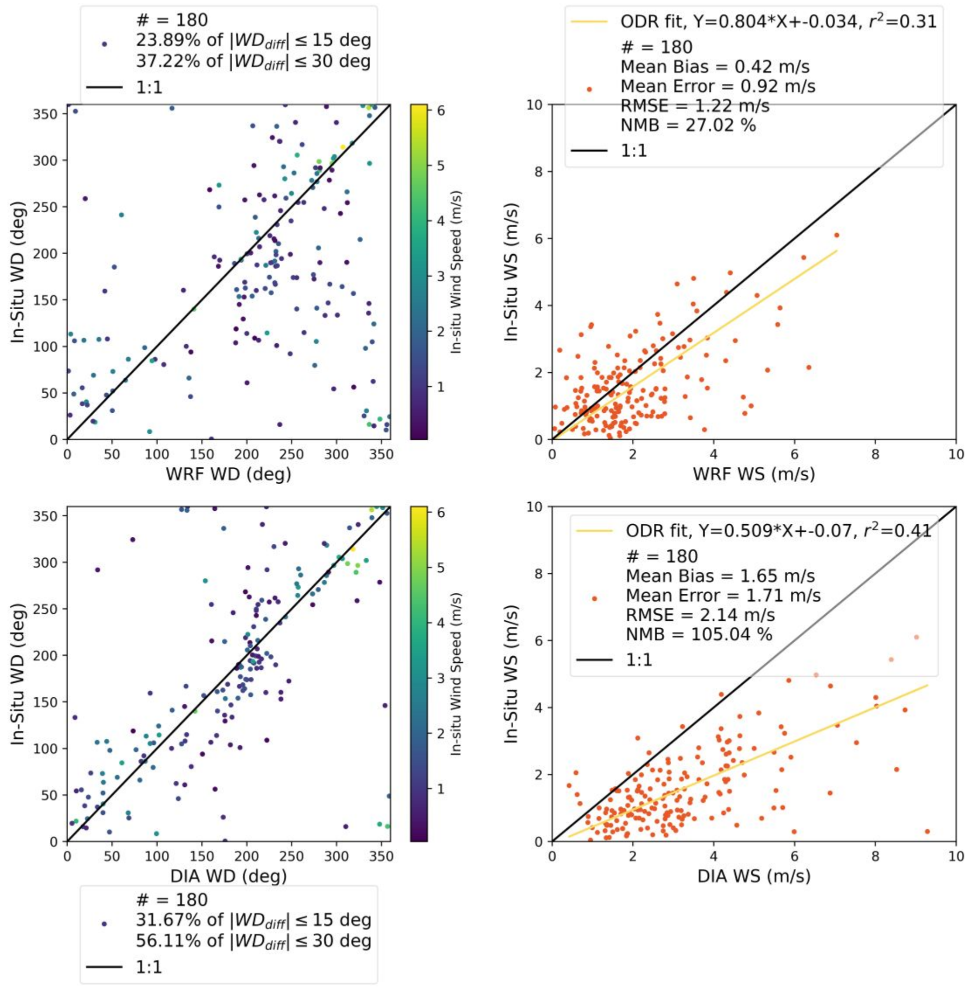

Figure S5. Daily wind direction (WD) and wind speed (WS) comparison between in-situ observations and WRF (top panels) & Denver International Airport (DIA) (bottom panels) from January to June 2022. For wind direction, scatter plots are colored by wind speed. Fractions of absolute wind direction differences $|WD_{diff}| \leq 15$ deg and 30 deg are included, respectively. For wind speed, statistical metrics are provided: mean bias, mean error, root mean square error (RMSE), and normalized mean bias (NMB). All in-situ wind speeds were corrected to the same altitude using Equation S1, as described in SI Text 3.

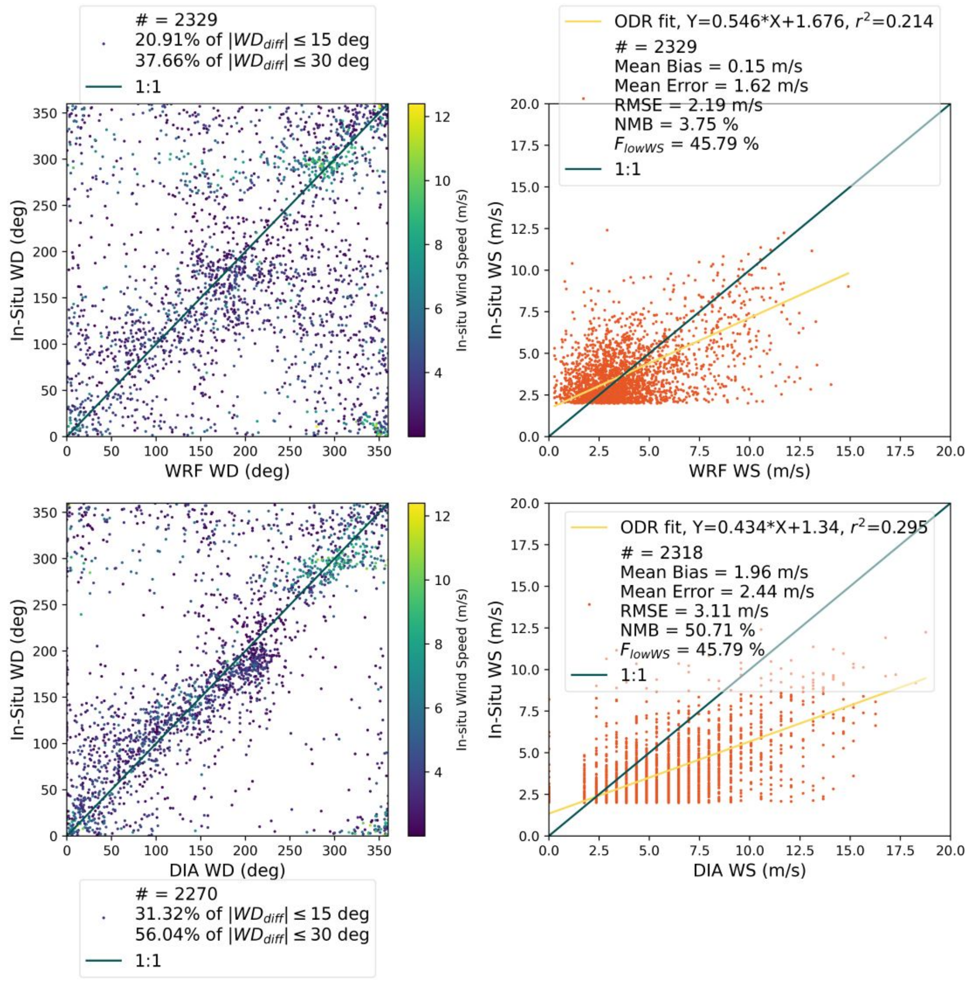

Figure S6. Hourly wind direction (WD) and wind speed (WS) comparison between in-situ observations and WRF (top panels) & Denver International Airport (DIA) (bottom panels) from January to June 2022, after removing low wind speed (in-situ WS < 2 m/s). For wind direction, scatter plots are colored by wind speed. Fractions of absolute wind direction differences $|WD_{diff}| \leq$ 15 deg and 30 deg are included, respectively. For wind speed, statistical metrics are provided: mean bias, mean error, root mean square error (RMSE), and normalized mean bias (NMB). All in-situ wind speeds were corrected to the same altitude using Equation S1, as described in SI Text 3. F_{lowWS} is the fraction of in-situ WS smaller than 2 m/s.

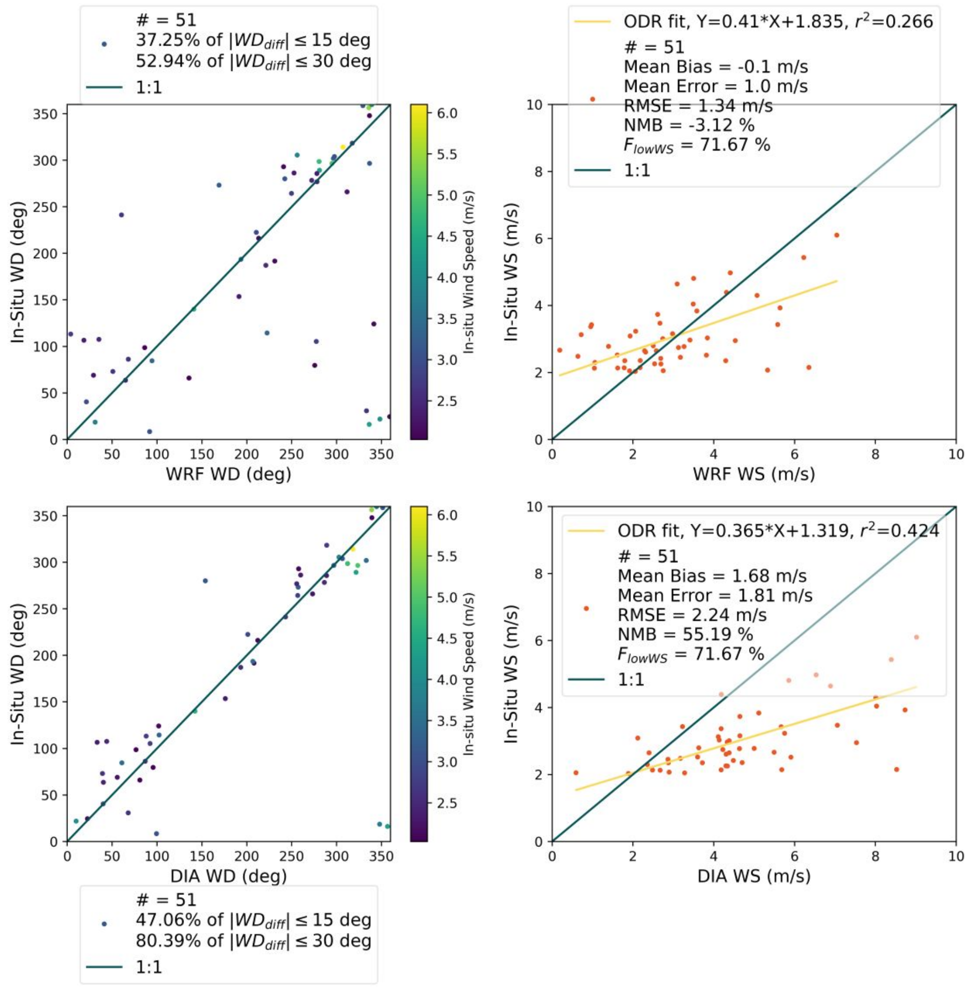

Figure S7. Daily wind direction (WD) and wind speed (WS) comparison between in-situ observations and WRF (top panels) & Denver International Airport (DIA) (bottom panels) from January to June 2022, after removing low wind speed (in-situ WS < 2 m/s). For wind direction, scatter plots are colored by wind speed. Fractions of absolute wind direction differences |WD_{diff}| ≤ 15 deg and 30 deg are included, respectively. For wind speed, statistical metrics are provided: mean bias, mean error, root mean square error (RMSE), and normalized mean bias (NMB). All in-situ wind speeds were corrected to the same altitude using Equation S1, as described in SI Text 3. F_{lowWS} is the fraction of in-situ WS smaller than 2 m/s.

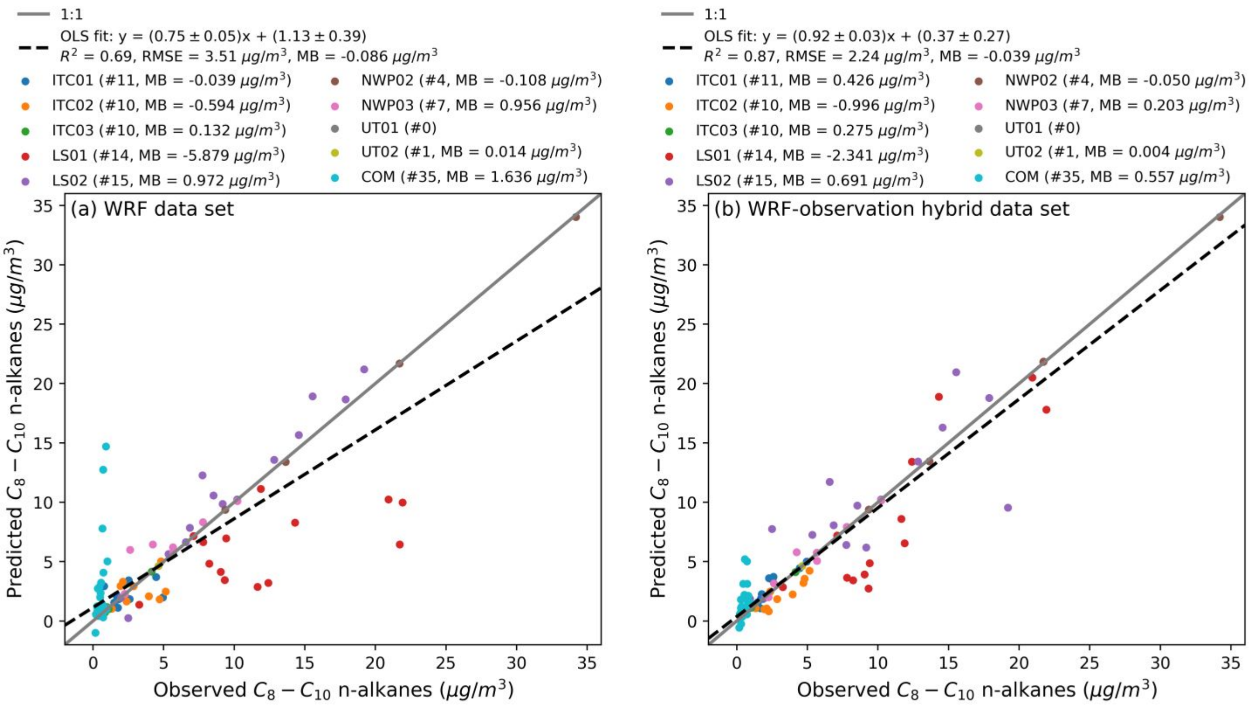

Figure S8. Predicted and observed C_8–C_{10} n-alkane concentrations for drilling operations. Observed concentrations are from weekly canister samples, while predicted concentrations are the AERMOD simulation results using the MLR constrained emission rates. Panels show results for (a) using WRF wind data set as AERMOD input and (b) using WRF-observation hybrid data set as AERMOD input. The solid gray line indicates the 1:1 line. The dashed black line represents the ordinary least squares (OLS) regression fit. The regression slope and intercept (±standard errors), R^2 value, root mean square error (RMSE), the number of weekly concentrations (#), and the mean bias (MB) are shown in each panel's legend. Each of the ten monitoring sites (ITC01, ITC02, ITC03, LS02, LS02, NWP02, NSP03, UT01, UT02, and COM) are shown in different colors.

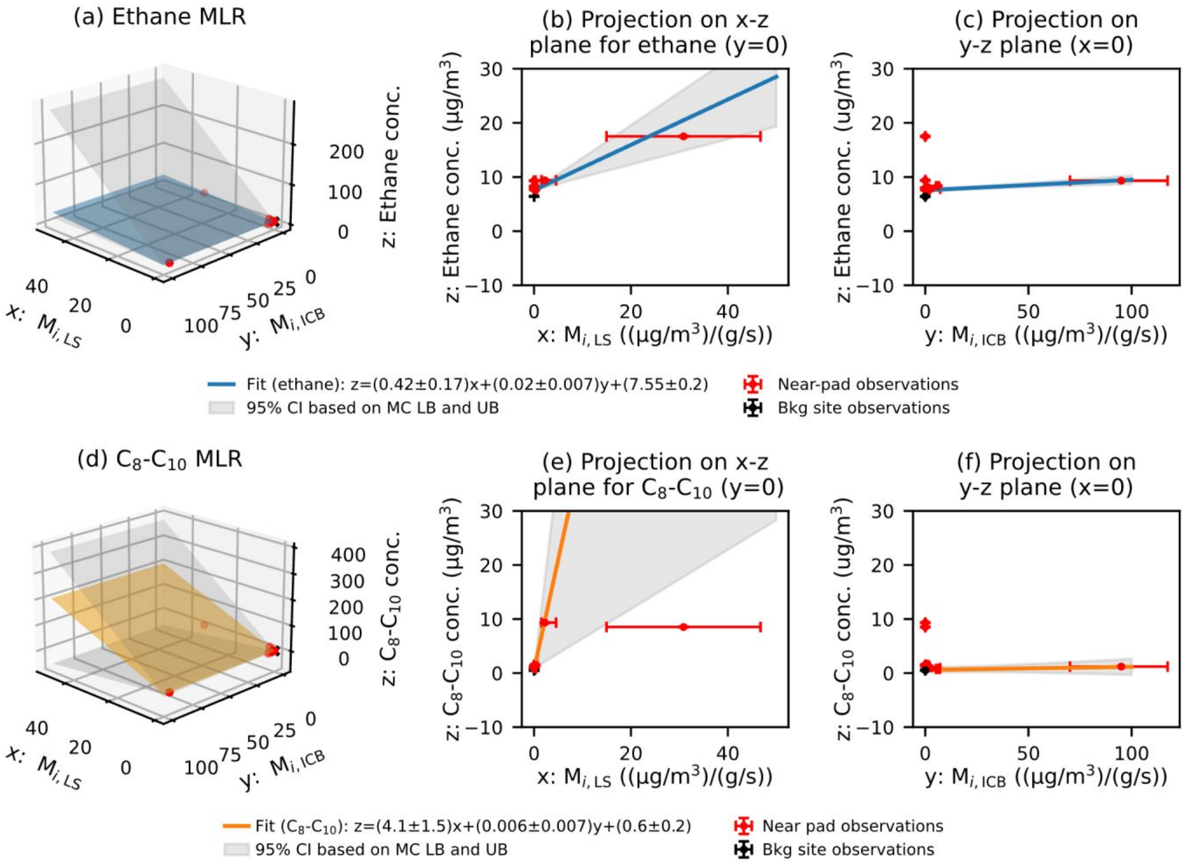

Figure S9. An example (July 11th, 2019) of the weekly multiple linear regression (MLR) method with absolute AERMOD uncertainties as weights. The slopes of the resulting best-fit planes in (a) and (d) correspond to the estimated emission rates for ethane and C_8–C_{10} n-alkanes, respectively. Two-dimensional projections (b, c, e, f) visualize the fit for each source; the solid line is the best-fit emission rate, while the shaded gray area represents the 95% confidence interval. Red and black dots are observations from near-source and background sites. The horizontal and vertical bars denote simulation and observation uncertainties, respectively.

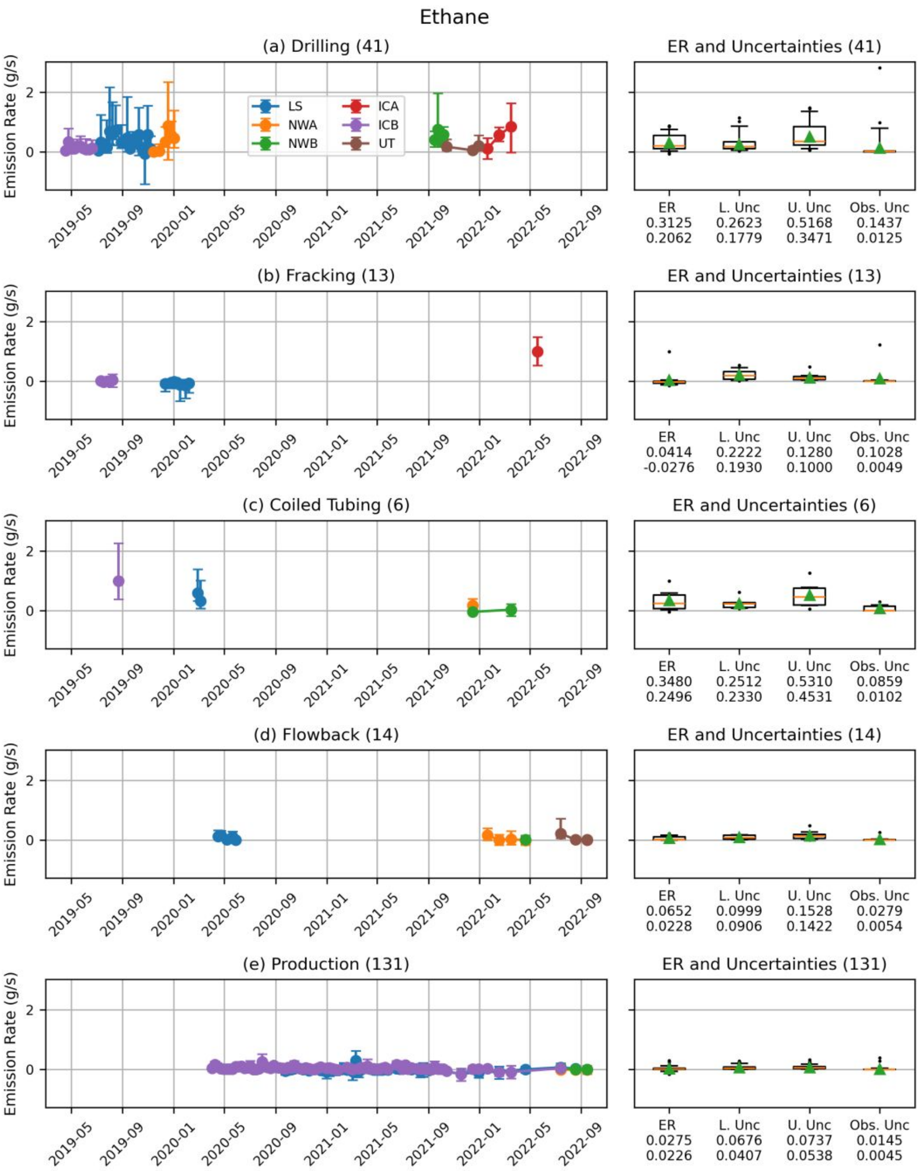

Figure S10. Quality controlled emission rates and uncertainties of ethane for (a) drilling, (b) fracking, (c) coiled tubing, (d) flowback, and (e) production. The panels on the left show the emission rates colored by source locations, and the error bars show 95% CIs. The boxes and whiskers show 5th, 25th, 50th, 75th, and 95th percentiles of the emission rates, overall uncertainties (L. Unc and U. Unc), and observation perturbation alone uncertainties (Obs. Unc). The triangles show mean values. The numeric values for the x-axis ticks show the mean and median values in the first and second rows, respectively.

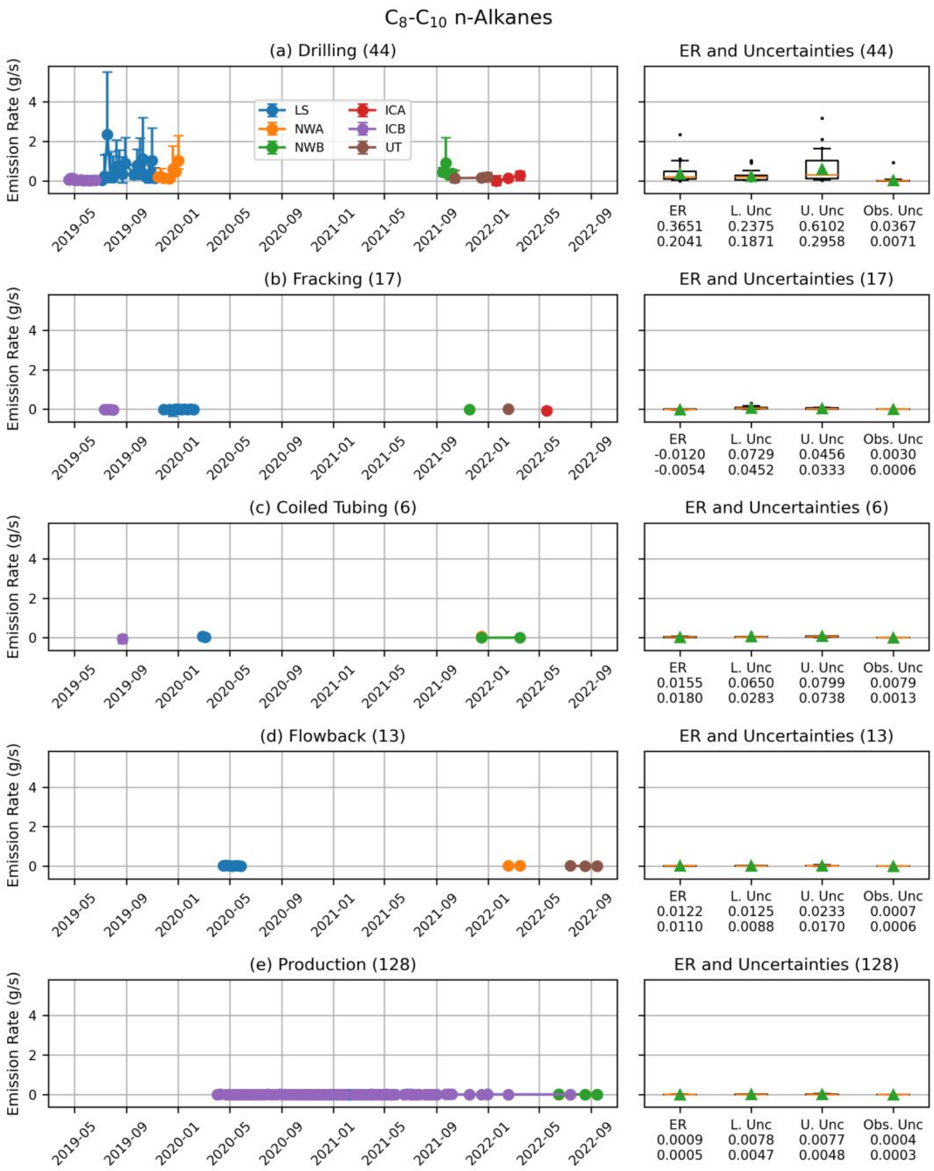

Figure S11. Quality controlled emission rates and uncertainties of C_8–C_{10} n-alkanes for (a) drilling, (b) fracking, (c) coiled tubing, (d) flowback, and (e) production. The panels on the left show the emission rates colored by source locations, and the error bars show 95% CIs. The boxes and whiskers show 5th, 25th, 50th, 75th, and 95th percentiles of the emission rates, overall uncertainties (L. Unc and U. Unc), and observation perturbation alone uncertainties (Obs. Unc). The triangles show mean values. The numeric values for the x-axis ticks show the mean and median values in the first and second rows, respectively.

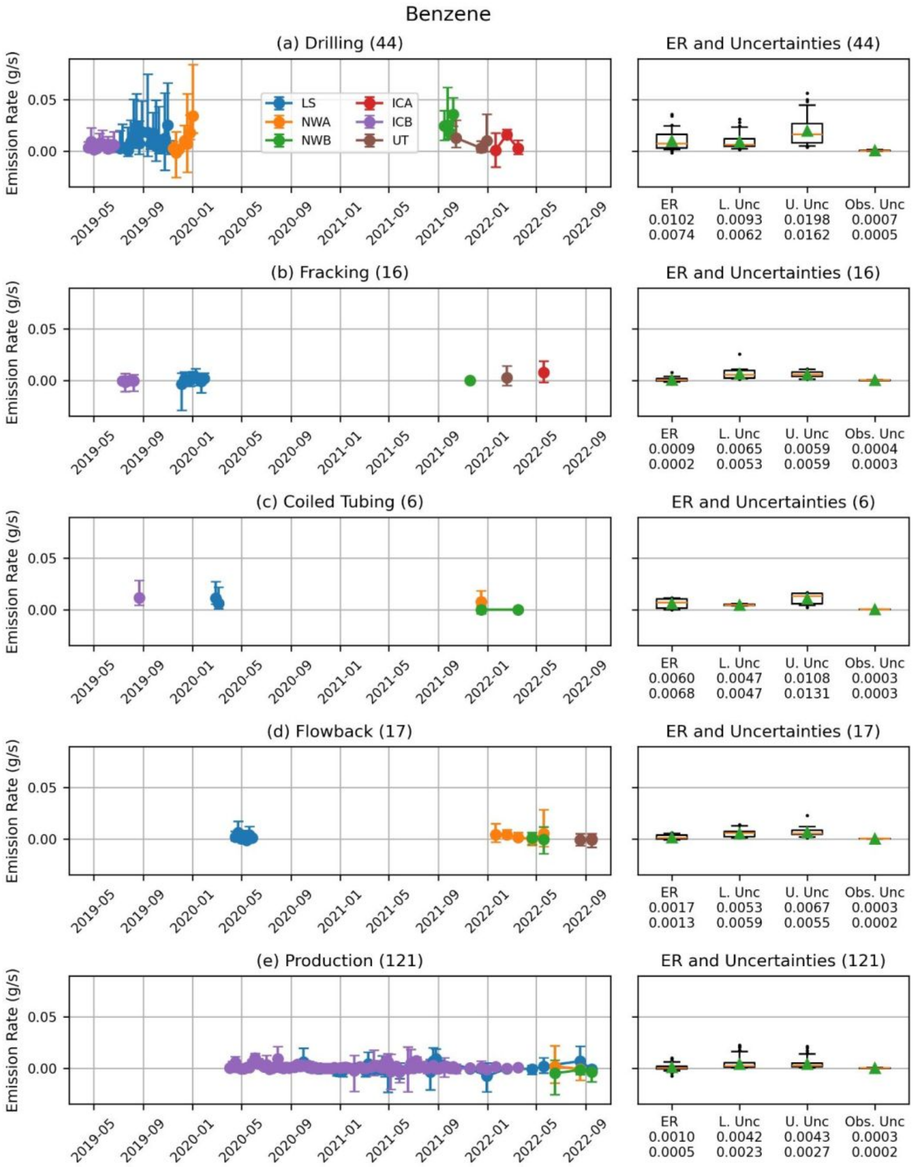

Figure S12. Quality controlled emission rates and uncertainties of benzene for (a) drilling, (b) fracking, (c) coiled tubing, (d) flowback, and (e) production. The panels on the left show the emission rates colored by source locations, and the error bars show 95% CIs. The boxes and whiskers show 5th, 25th, 50th, 75th, and 95th percentiles of the emission rates, overall uncertainties (L. Unc and U. Unc), and observation perturbation alone uncertainties (Obs. Unc). The triangles show mean values. The numeric values for the x-axis ticks show the mean and median values in the first and second rows, respectively.

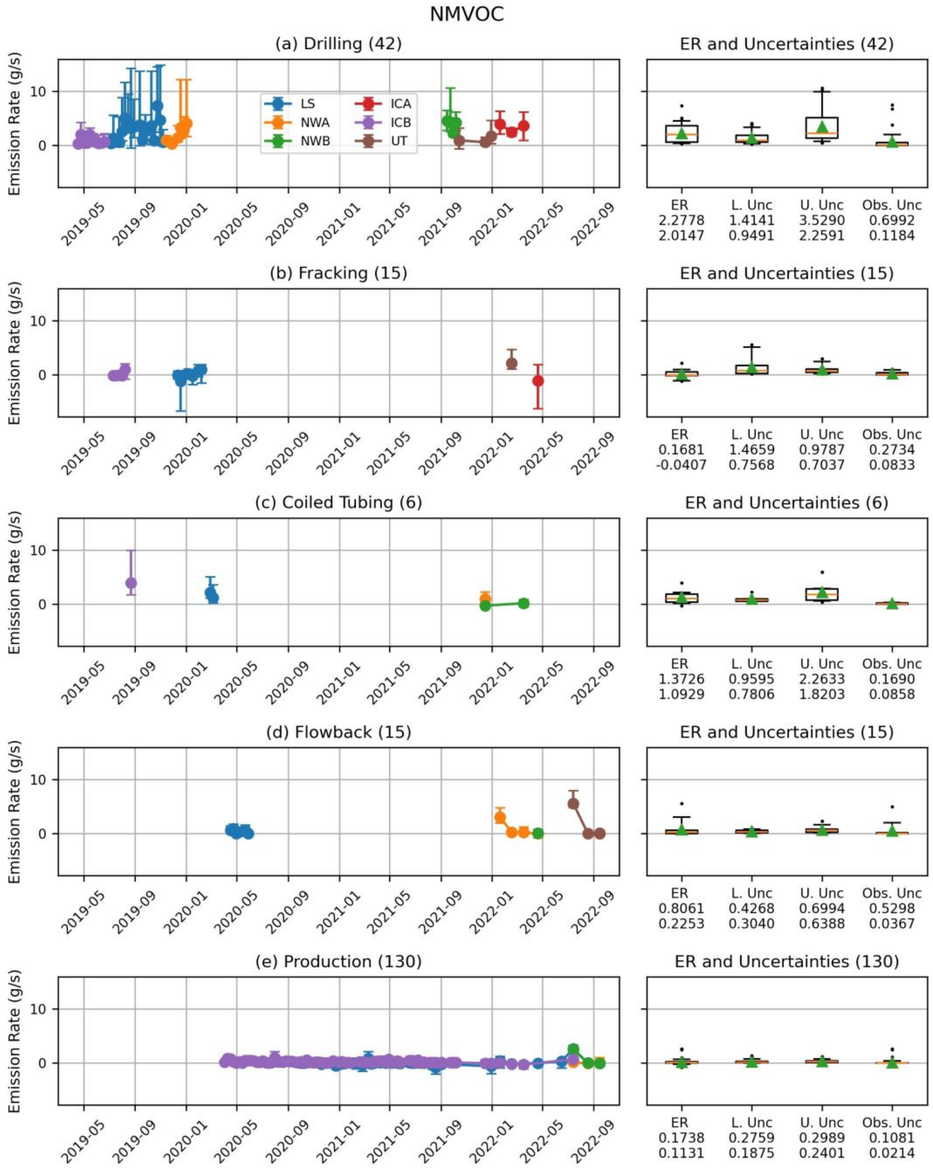

Figure S13. Quality controlled emission rates and uncertainties of NMVOC for (a) drilling, (b) fracking, (c) coiled tubing, (d) flowback, and (e) production. The panels on the left show the emission rates colored by source locations, and the error bars show 95% CIs. The boxes and whiskers show 5th, 25th, 50th, 75th, and 95th percentiles of the emission rates, overall uncertainties (L. Unc and U. Unc), and observation perturbation alone uncertainties (Obs. Unc). The triangles show mean values. The numeric values for the x-axis ticks show the mean and median values in the first and second rows, respectively.

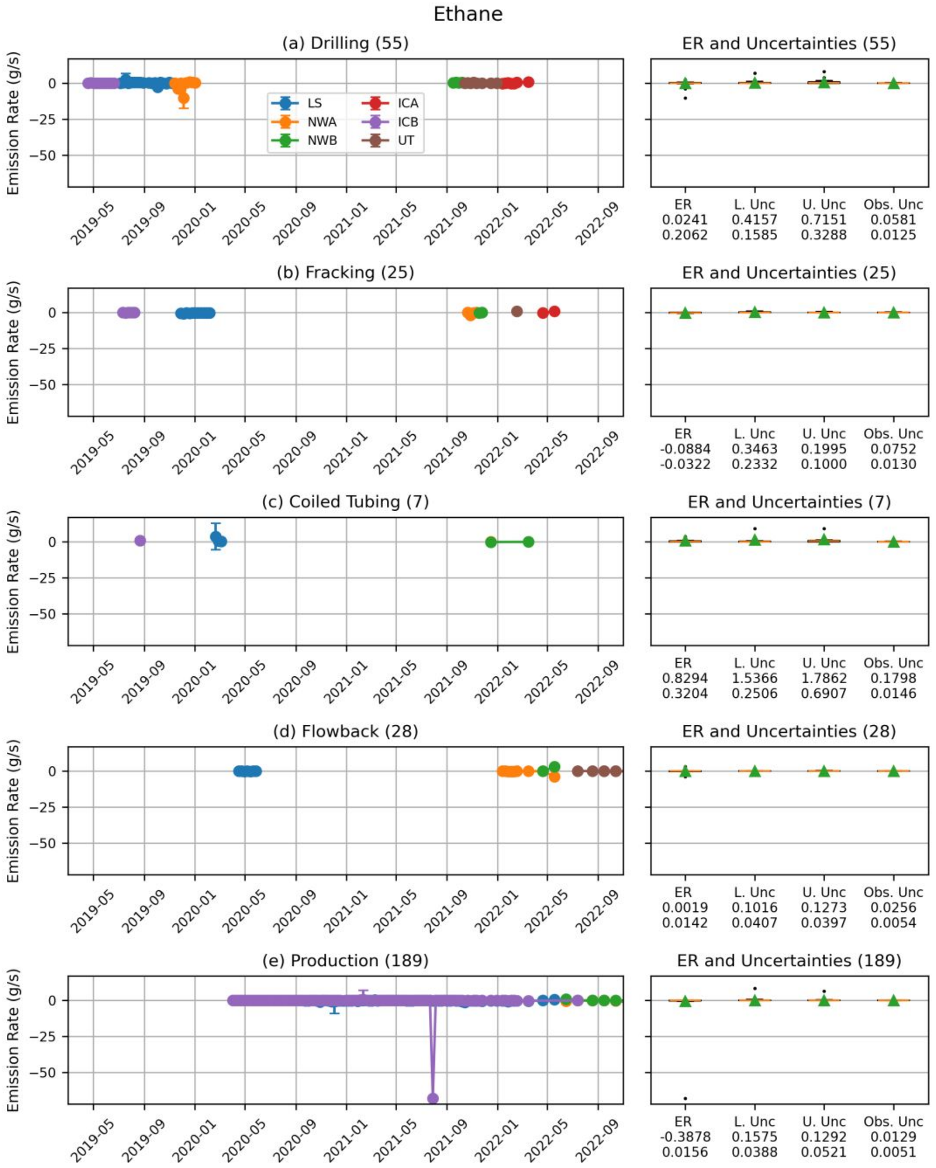

Figure S14. All emission rates and uncertainties of ethane for (a) drilling, (b) fracking, (c) coiled tubing, (d) flowback, and (e) production. The panels on the left show the emission rates colored by source locations, and the error bars show 95% CIs. The boxes and whiskers show 5th, 25th, 50th, 75th, and 95th percentiles of the emission rates, overall uncertainties (L. Unc and U. Unc), and observation perturbation alone uncertainties (Obs. Unc). The triangles show mean values. The numeric values for the x-axis ticks show the mean and median values in the first and second rows, respectively.

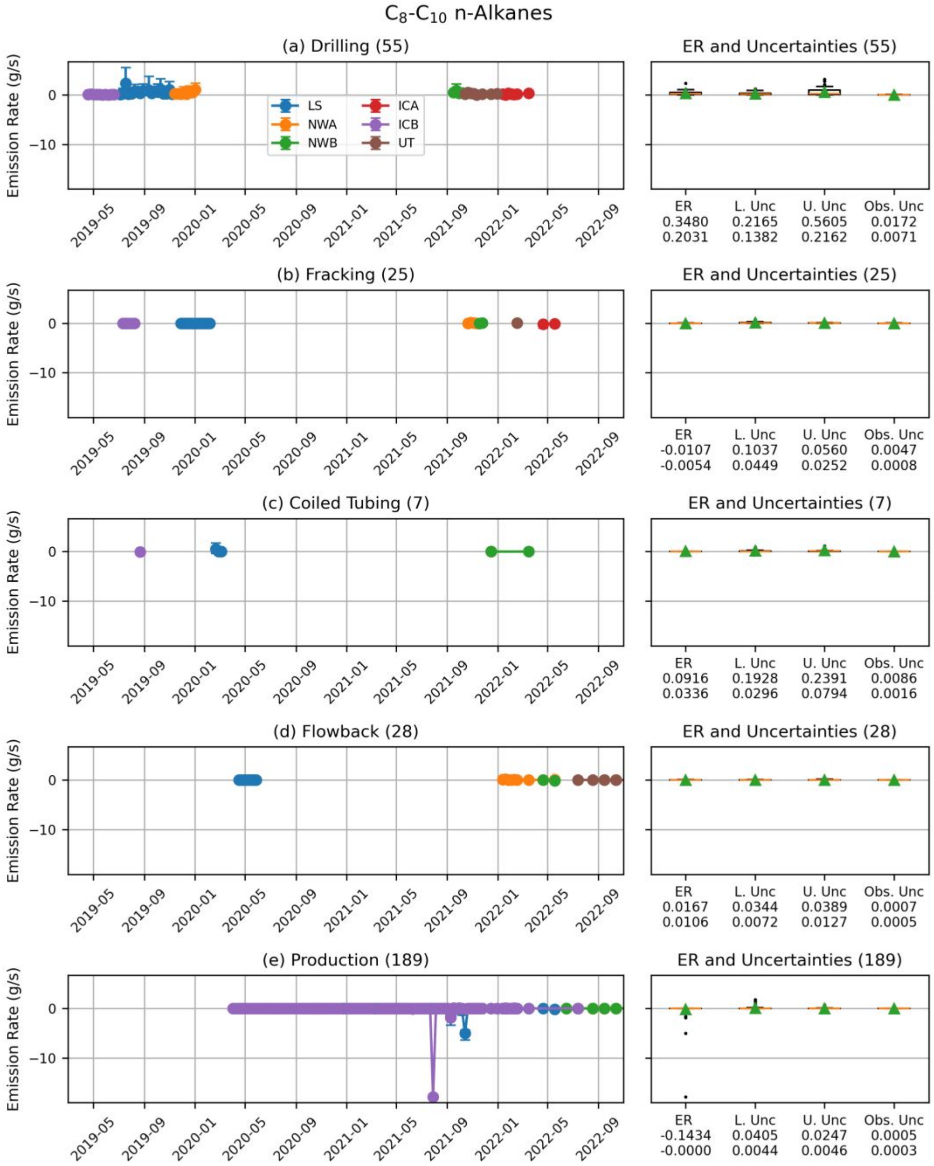

Figure S15. All emission rates and uncertainties of C_8–C_{10} n-alkanes for (a) drilling, (b) fracking, (c) coiled tubing, (d) flowback, and (e) production. The panels on the left show the emission rates colored by source locations, and the error bars show 95% CIs. The boxes and whiskers show 5th, 25th, 50th, 75th, and 95th percentiles of the emission rates, overall uncertainties (L. Unc and U. Unc), and observation perturbation alone uncertainties (Obs. Unc). The triangles show mean values. The

numeric values for the x-axis ticks show the mean and median values in the first and second rows, respectively.

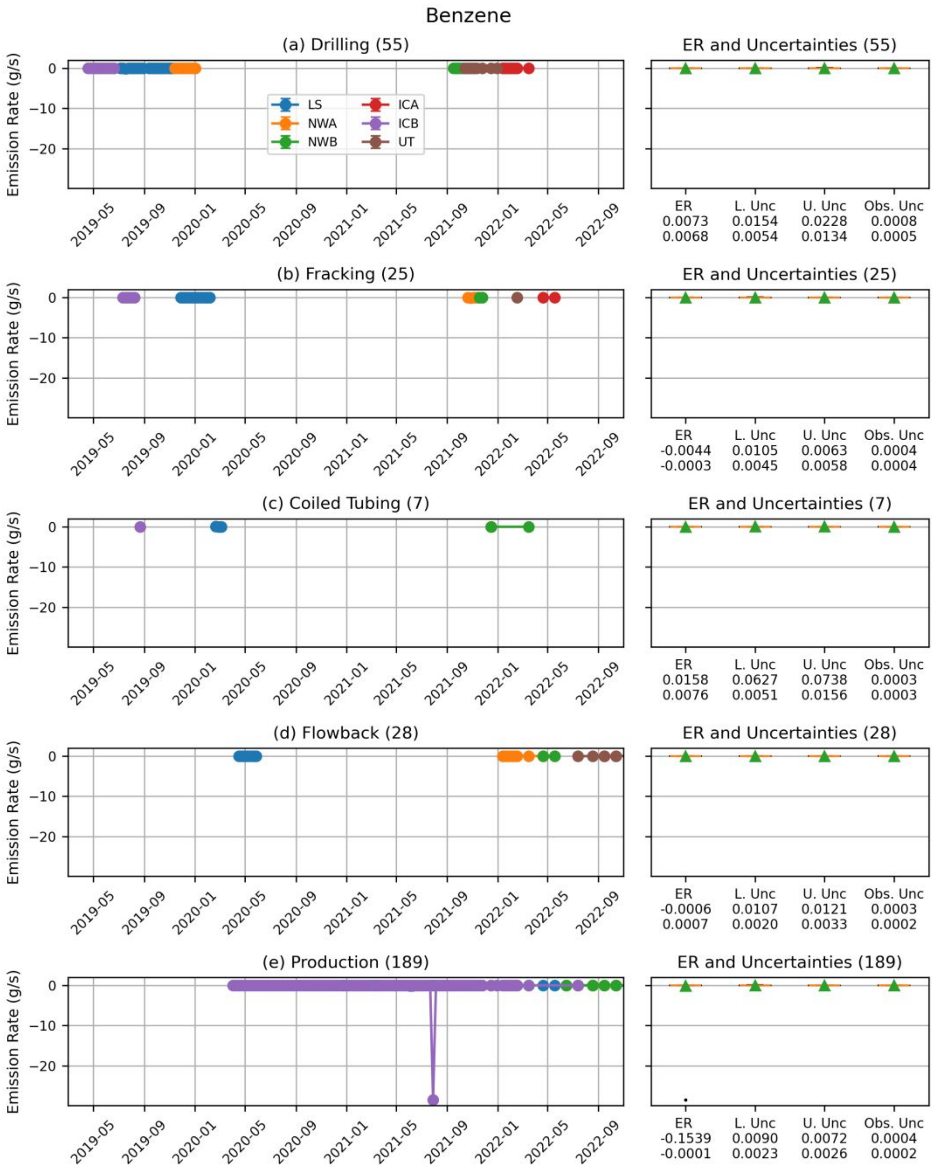

Figure S16. All emission rates and uncertainties of benzene for (a) drilling, (b) fracking, (c) coiled tubing, (d) flowback, and (e) production. The panels on the left show the emission rates colored by source locations, and the error bars show 95% CIs. The boxes and whiskers show 5th, 25th, 50th, 75th, and 95th percentiles of the emission rates, overall uncertainties (L. Unc and U. Unc), and observation perturbation alone uncertainties (Obs. Unc). The triangles show mean values. The

numeric values for the x-axis ticks show the mean and median values in the first and second rows, respectively.

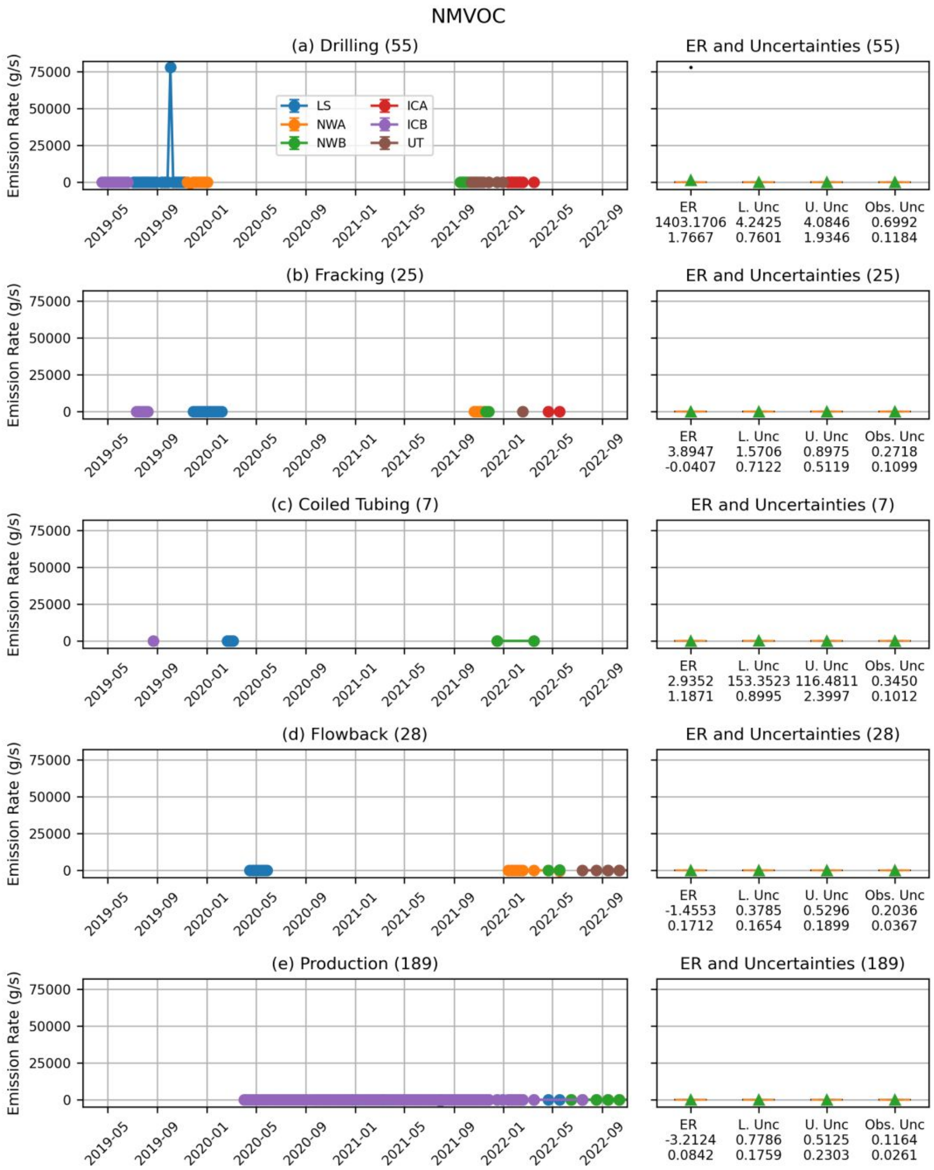

Figure S17. All emission rates and uncertainties of NMVOC for (a) drilling, (b) fracking, (c) coiled tubing, (d) flowback, and (e) production. The panels on the left show the emission rates colored by source locations, and the error bars show 95% CIs. The boxes and whiskers show 5th, 25th, 50th, 75th, and 95th percentiles of the emission rates, overall uncertainties (L. Unc and U. Unc), and observation perturbation alone uncertainties (Obs. Unc). The triangles show mean values. The

numeric values for the x-axis ticks show the mean and median values in the first and second rows, respectively.

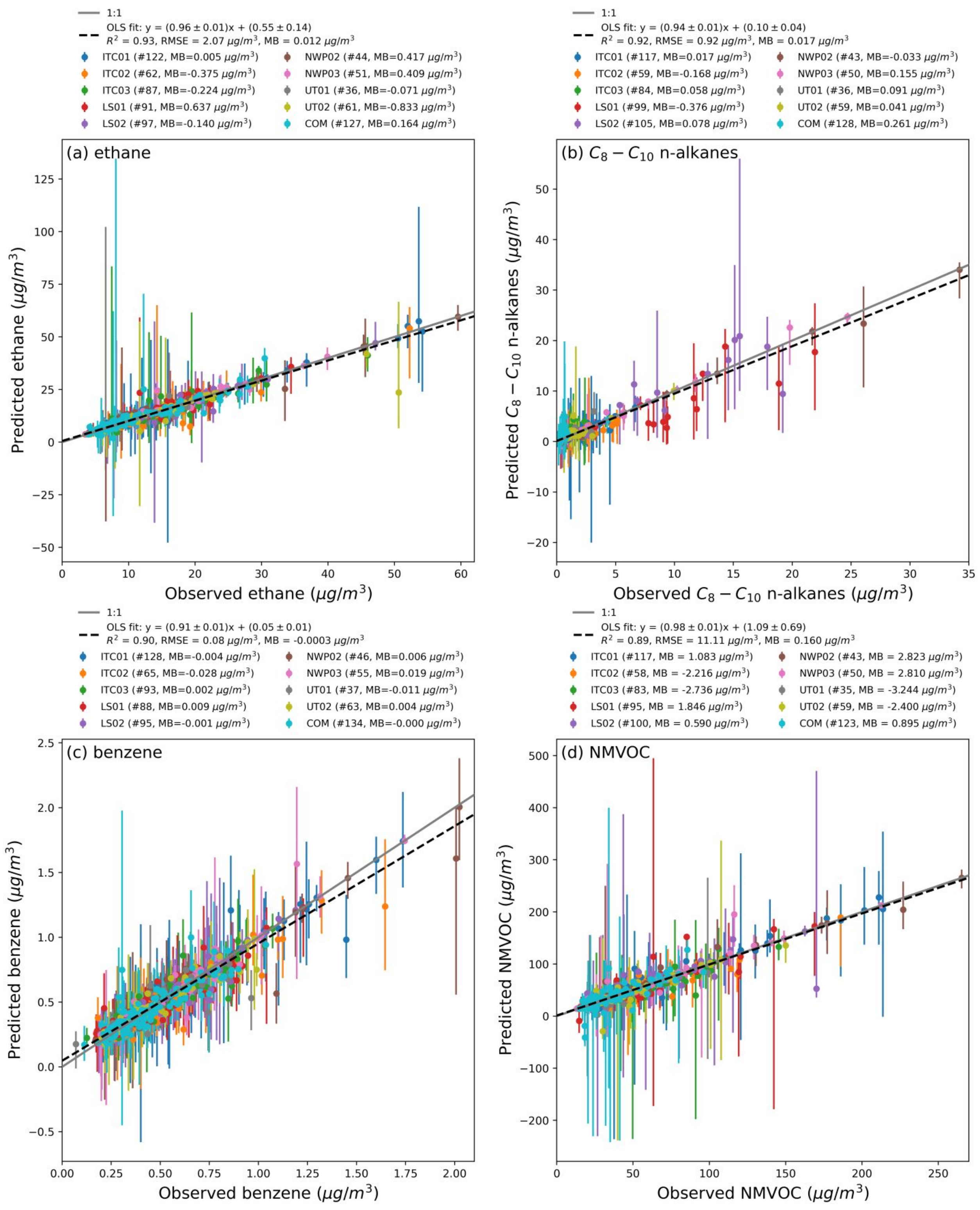

Figure S18. Predicted and observed VOC concentrations for all operations. Observed concentrations are from weekly canister samples, while predicted concentrations are the

AERMOD simulation results using the MLR constrained emission rates. Panels show results for (a) ethane, (b) C_8–C_{10} n-alkanes, (c) benzene, and (d) NMVOC. The solid gray line indicates the 1:1 line. The dashed black line represents the ordinary least squares (OLS) regression fit. The regression slope and intercept (±standard errors), R^2, root mean squared error (RMSE), the number of weekly concentrations (#), and the mean bias (MB) are shown in each panel's legend. Each of the ten monitoring sites (ITC01, ITC02, ITC03, LS02, LS02, NWP02, NSP03, UT01, UT02, and COM) are shown in different colors.

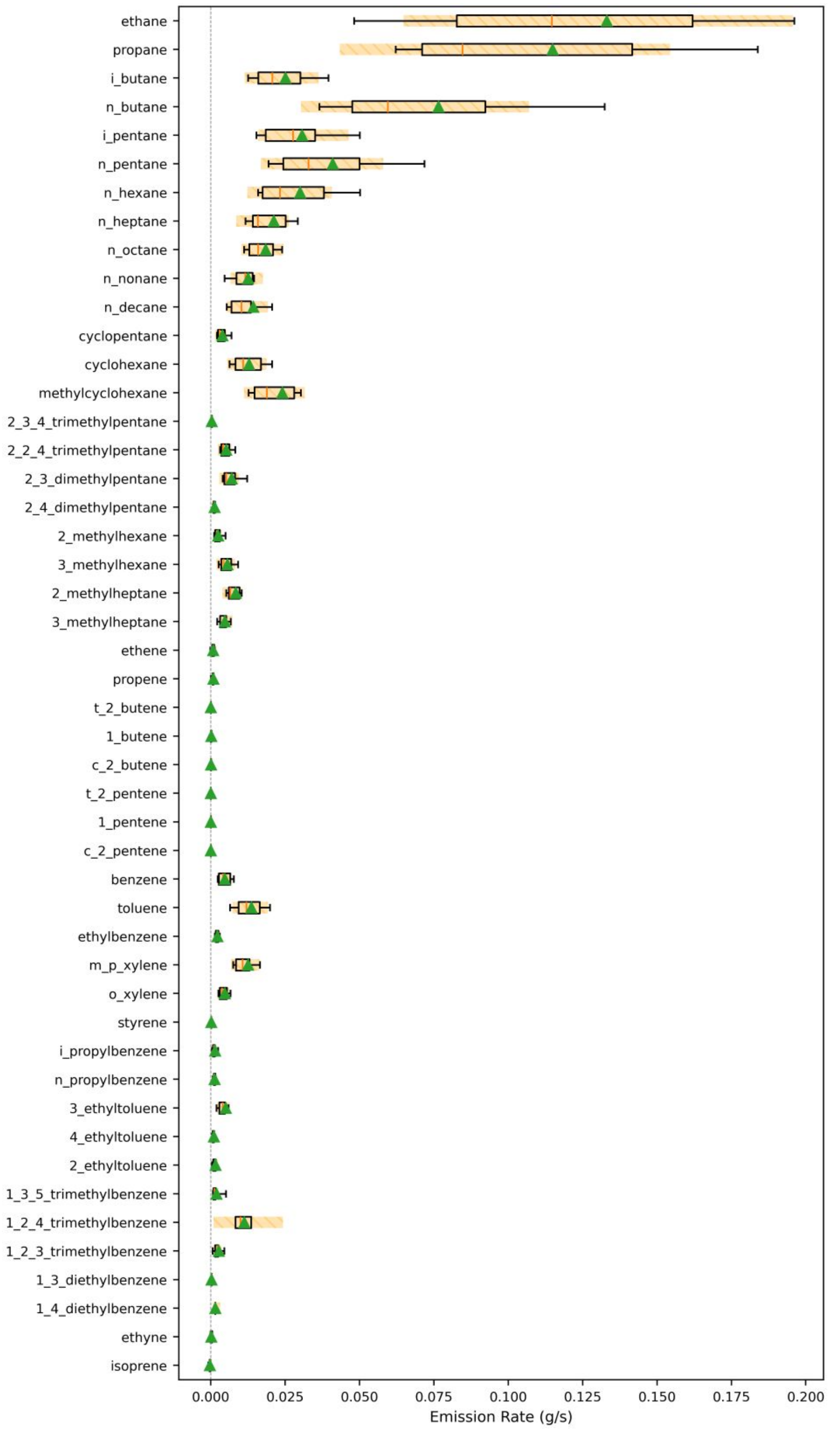

605

606 **Figure S19**. Emission rates of 48 VOCs during drilling operation using Gibson mud. The boxes

607 and whiskers represent the 5th, 25th, 75th, and 95th percentiles, respectively. Orange line and green

triangle represent the median and mean, respectively. The orange shaded area represents the aggregated 95% confidence intervals (CIs) for median emission rates.

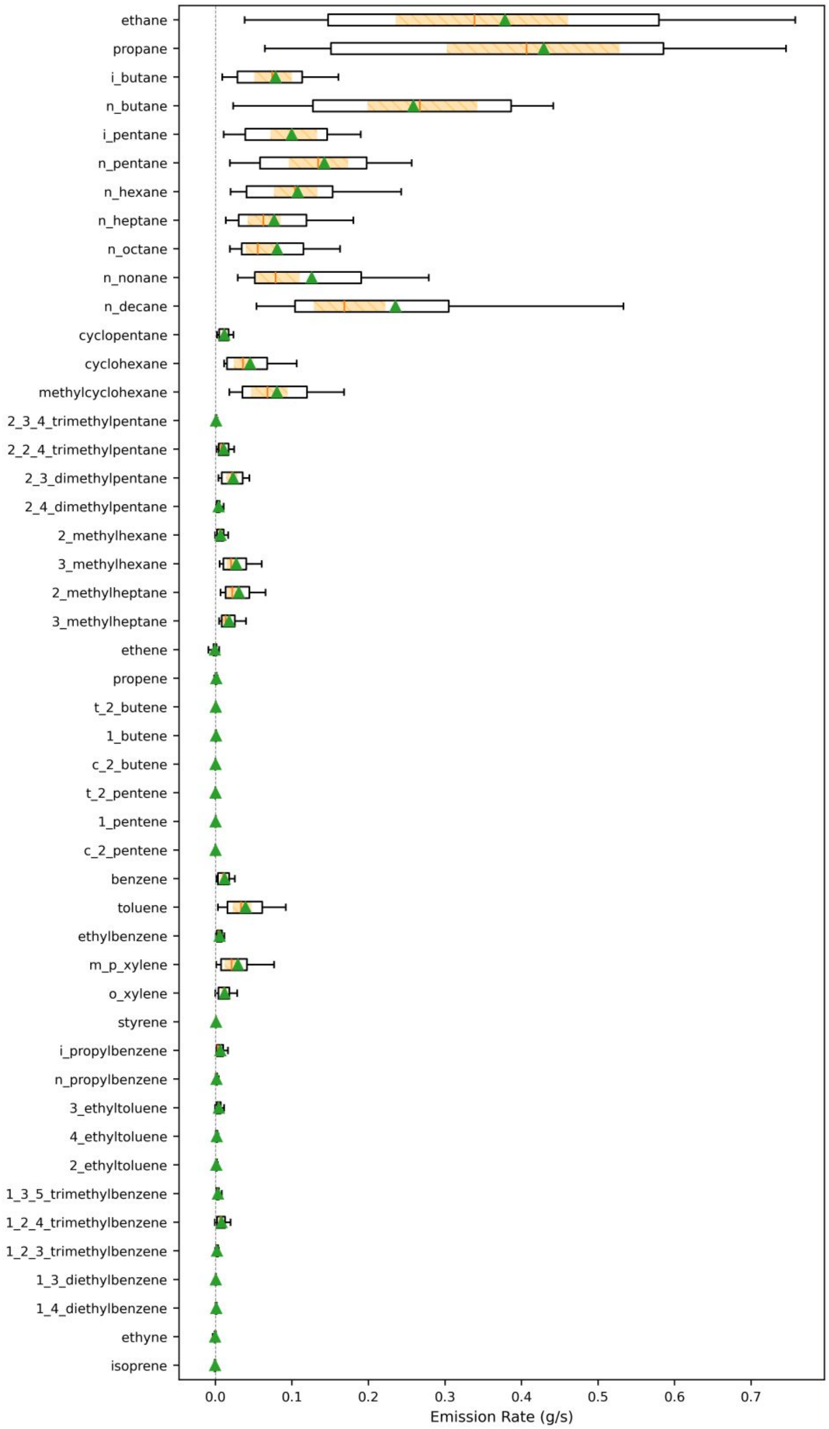

Figure S20. Emission rates of 48 VOCs during drilling operation using Neoflo mud. The boxes and whiskers represent the 5th, 25th, 75th, and 95th percentiles, respectively. Orange line and green triangle represent the median and mean, respectively. The orange shaded area represents the aggregated 95% confidence intervals (CIs) for median emission rates.

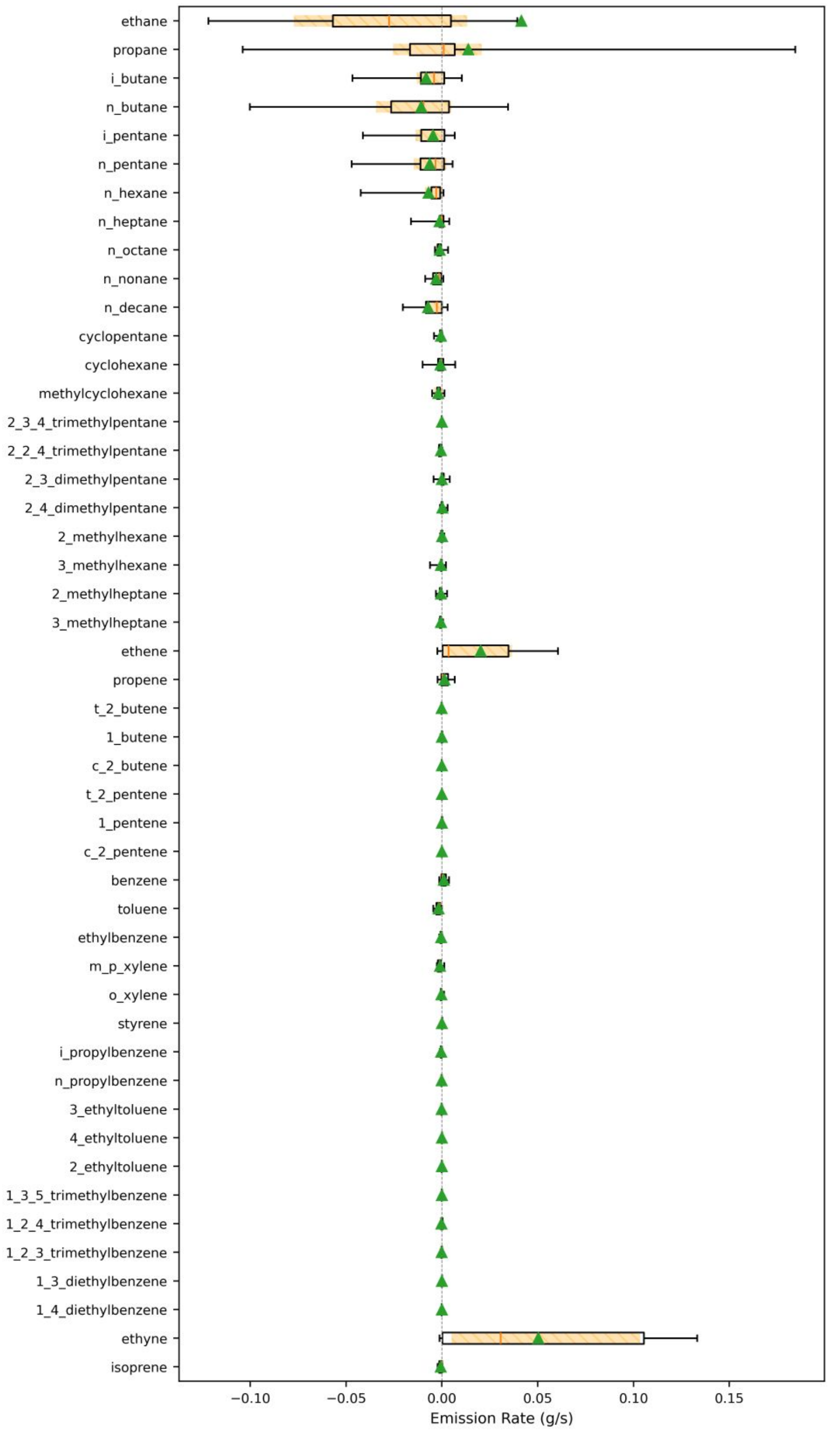

Figure S21. Emission rates of 48 VOCs during hydraulic fracturing. The boxes and whiskers represent the 5th, 25th, 75th, and 95th percentiles, respectively. Orange line and green triangle

represent the median and mean, respectively. The orange shaded area represents the aggregated 95% confidence intervals (CIs) for median emission rates.

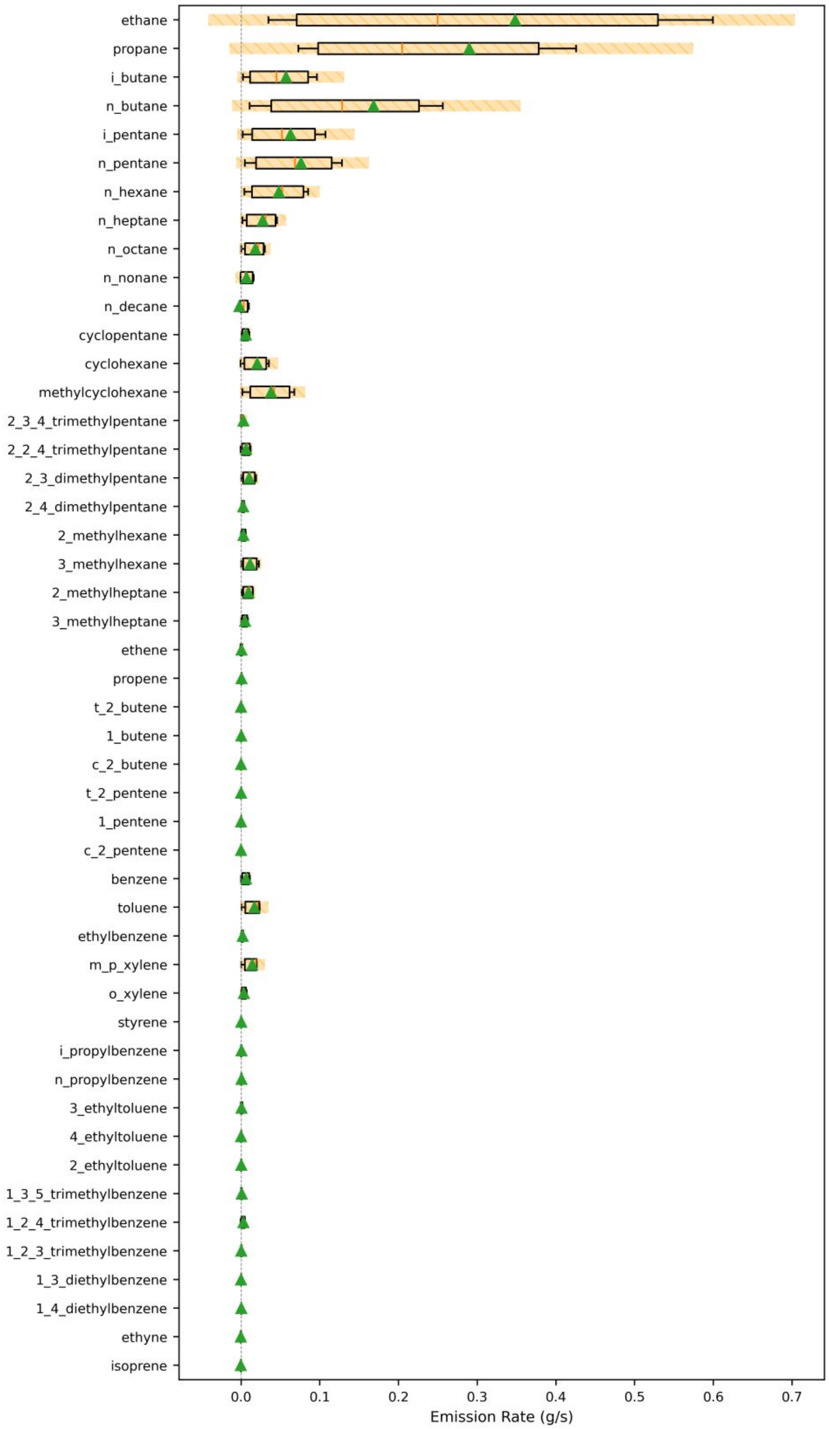

Figure S22. Emission rates of 48 VOCs during coiled tubing/millout. The boxes and whiskers represent the 5th, 25th, 75th, and 95th percentiles, respectively. Orange line and green triangle represent the median and mean, respectively. The orange shaded area represents the aggregated 95% confidence intervals (CIs) for median emission rates.

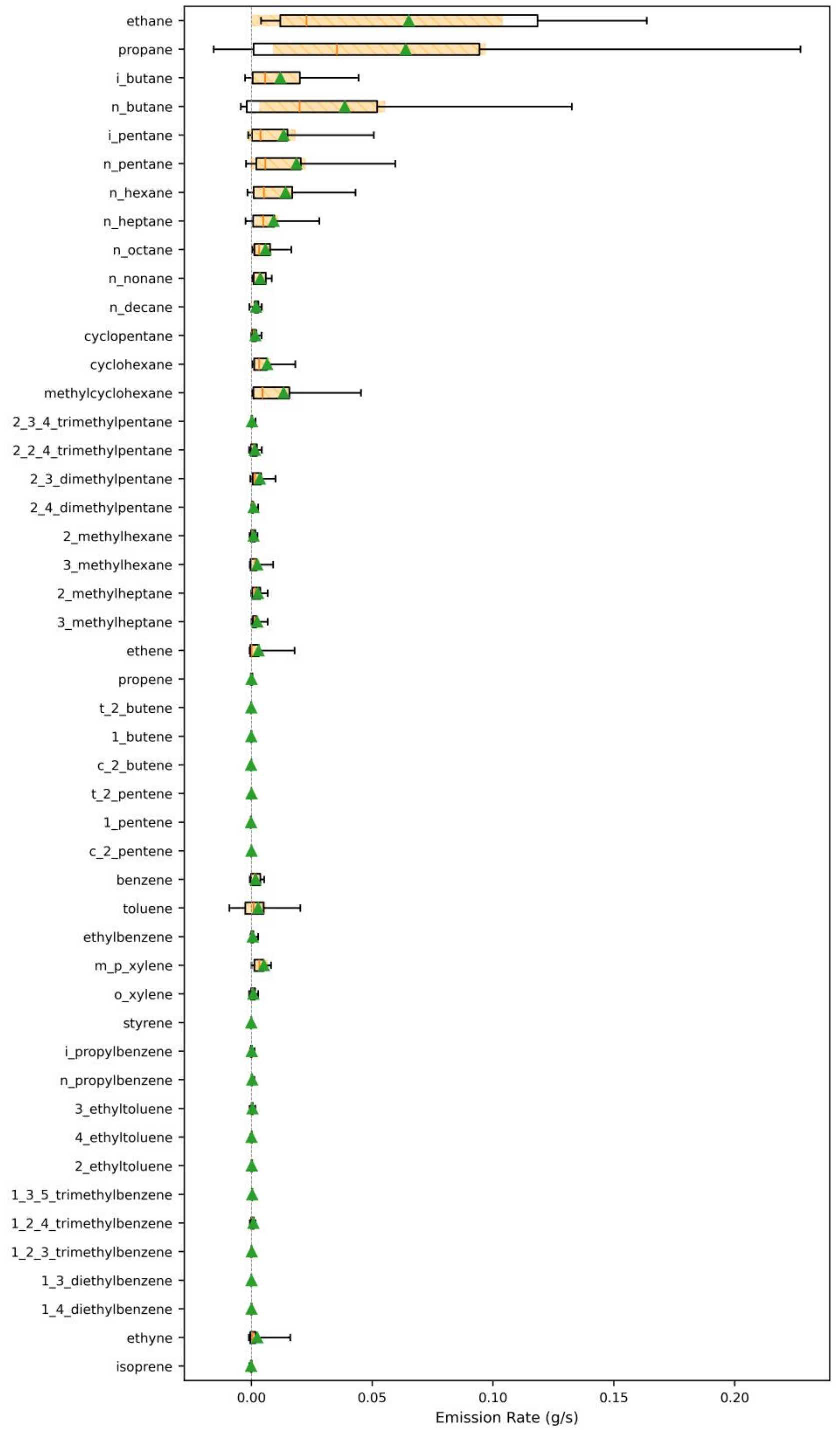

Figure S23. Emission rates of 48 VOCs during flowback. The boxes and whiskers represent the 5th, 25th, 75th, and 95th percentiles, respectively. Orange line and green triangle represent the median

and mean, respectively. The orange shaded area represents the aggregated 95% confidence intervals (CIs) for median emission rates.

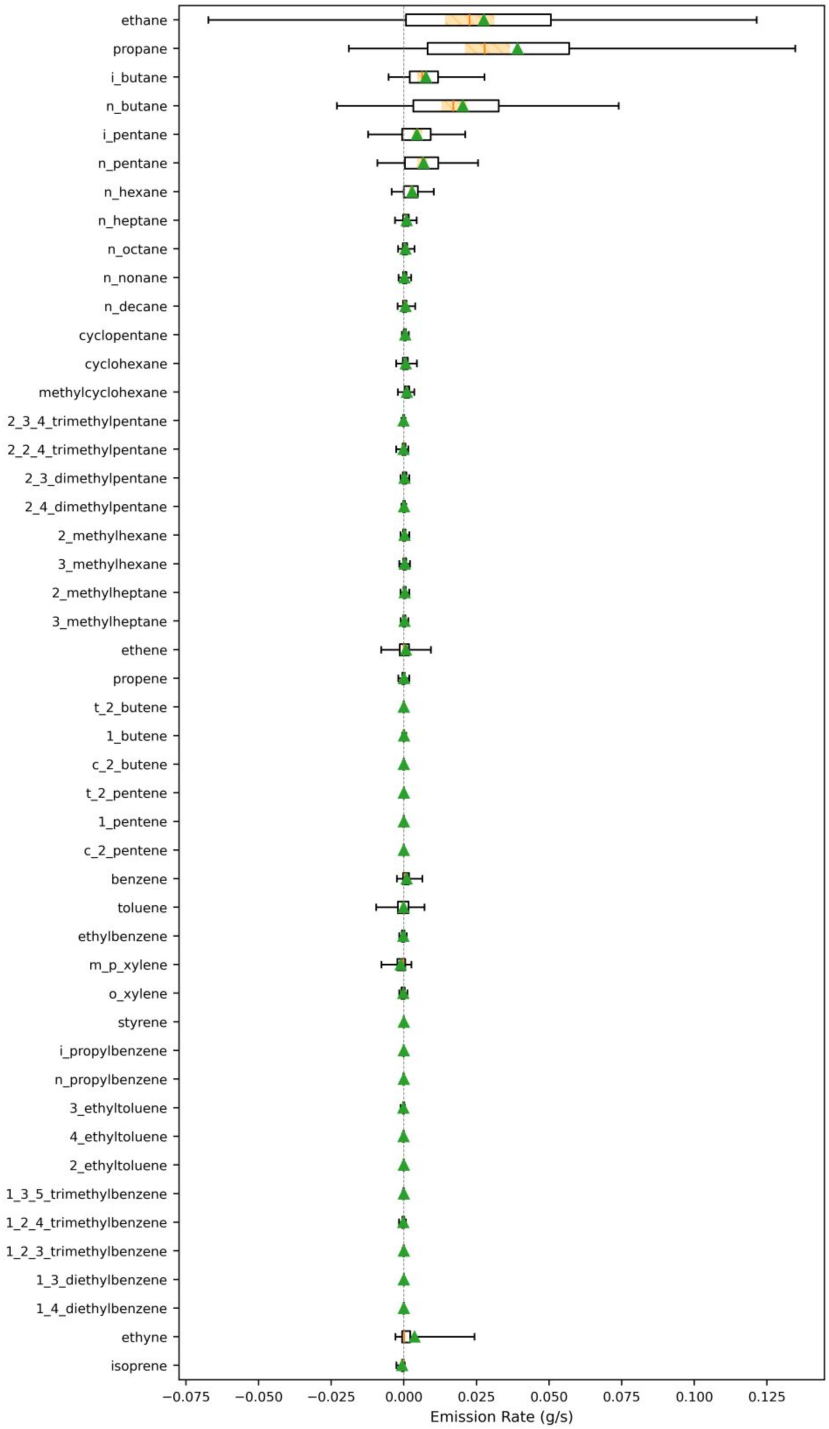

Figure S24. Emission rates of 48 VOCs during production. The boxes and whiskers represent the 5th, 25th, 75th, and 95th percentiles, respectively. Orange line and green triangle represent the median and mean, respectively. The orange shaded area represents the aggregated 95% confidence intervals (CIs) for median emission rates.

Supplementary Tables

Table S1. Number of weekly whole air canister samples collected at 10 monitoring sites in Broomfield between the listed start date and end date.

Monitoring Site	Start Date	End Date	# of Weekly Samples
COM	2019-04-18	2022-12-29	192
ITC01	2019-04-18	2022-12-29	191
ITC02	2019-04-18	2022-09-22	71
ITC03	2019-04-18	2022-12-23	101
LS01	2019-04-18	2022-12-29	182
LS02	2019-04-18	2022-09-22	131
NWP02	2019-04-18	2022-09-22	49
NWP03	2019-04-18	2022-12-23	99
UT01	2019-04-18	2022-12-23	41
UT02	2019-04-18	2022-12-29	114

Table S2. Measurement uncertainties for 48 VOCs.

VOC	Measurement Uncertainty (%)	VOC	Measurement Uncertainty (%)
ethane	6.55	t-2-butene	5.4
propane	5.46	1-butene	5.35
i-butane	5.35	c-2-butene	5.32
n-butane	5.51	t-2-pentene	5.74
i-pentane	5.34	1-pentene	6.71
n-pentane	5.64	cis-2-pentene	7.68
n-hexane	5.84	benzene	5.77
n-heptane	6.25	toluene	5.74
n-octane	5.67	ethylbenzene	6.04
n-nonane	6.02	m+p-xylene	6.58
n-decane	6.54	o-xylene	6.19
cyclopentane	5.36	styrene	23.51
cyclohexane	5.52	i-propylbenzene	22.23
methylcyclohexane	6.27	n-propylbenzene	16.71
2,3,4-trimethylpentane (tmp)	5.63	3-ethyltoluene	18.45
2,2,4-tmp	16.9	4-ethyltoluene	23.53
2,3-dimethylpentane (dmp)	5.48	2-ethyltoluene	19.98
2,4-dmp	6.82	1,3,5-trimethylbenzene	13.93
2-methylhexane	5.67	1,2,4-trimethylbenzene	18.68
3-methylhexane	5.85	1,2,3-trimethylbenzene	20.66
2-methylheptane	5.68	1,3-diethylbenzene	15.42
3-methylheptane	5.7	1,4-diethylbenzene	18.72
ethene	5.54	ethyne	6.26
propene	5.7	isoprene	6.54

Table S3. Initial concentrations and 7-day recovery of VOC compounds from afternoon ambient air samples collected in Entech silonite-coated 6 L canisters with 56 ppbv of ozone.

**Compound**	**Initial concentration (ppbv)**	**7-day recovery (%)**
ethene	0.143	99.8
isoprene	0.757	95.8
ethane	1.66	99.3
propane	0.606	101.4
n-butane	0.291	100.9
ethyne	0.106	98.9
i-pentane	0.275	94.3
n-pentane	0.185	100.2

Table S4. WRF-ARW Parameterization Schemes.

WRF Physics Option Types	Physics Option Selected
Microphysics	WRF single-moment 3-class scheme
Long-wave radiation	RRTMG longwave scheme
Short-wave radiation	RRTMG shortwave scheme
Surface layer	Revised MM5 scheme
Land surface	Unified Noah land surface model
Planetary boundary layer	Yonsei university (YSU) scheme
Cumulus parameterization	Kain-Fritsch scheme (grid size > 10 km only)

Table S5. Number of emission rates excluded by quality control procedures. See SI Text 4.6 for more details.

Compound	Drilling	Fracking	Coiled Tubing	Flowback	Production
**Total Number of Emission Rates (Same for All Compounds)**					
	55	25	7	28	189
**Number of Emission Rates Excluded by Quality Control Procedures.**					
ethane	14	12	1	14	58
propane	12	8	1	12	55
i-butane	11	7	1	11	57
n-butane	11	8	1	11	54
i-pentane	11	8	1	12	56
n-pentane	11	7	1	10	55
n-hexane	10	7	1	10	55
n-heptane	10	7	1	10	58
n-octane	12	8	1	13	57
n- nonane	10	7	1	13	65
n-decane	12	7	1	15	74
cyclopentane	10	7	1	10	58
cyclohexane	10	7	1	11	60
methylcyclohexane	12	7	1	12	63
2,3,4-trimethylpentane	17	10	1	12	71
2,2,4-trimethylpentane	10	8	1	11	53
2,3-dimethylpentane	10	8	1	11	60
2,4-dimethylpentane	11	7	1	11	64
2-methylhexane	10	9	1	10	67
3-methylhexane	10	8	1	11	64
2-methylheptane	11	8	1	13	62
3-methylheptane	13	7	1	13	63
ethene	16	8	1	13	61
propene	13	8	1	15	68
t-2-butene	17	7	1	14	69
1-butene	15	8	2	15	64
c-2-butene	18	8	1	15	70
t-2-pentene	18	9	3	13	73
1-pentene	18	6	2	15	80
c-2-pentene	18	8	1	14	69
benzene	11	9	1	11	68
toluene	12	10	1	13	63
ethylbenzene	13	7	1	11	68

styrene	12	6	1	14	61

Table S5 (Continued).

m+p-xylene	12	8	1	13	57
o-xylene	11	8	1	10	65
i-propylbenzene	11	5	1	10	61
n-propylbenzene	12	9	1	12	59
3-ethyltoluene	17	8	1	11	57
4-ethyltoluene	17	7	1	12	57
1,3,5-trimethylbenzene	17	10	1	13	58
2-ethyltoluene	15	9	1	12	59
1,2,4-trimethylbenzene	11	8	1	12	56
1,2,3-trimethylbenzene	17	9	0	11	58
1,3-diethylbenzene	15	8	1	11	60
1,4-diethylbenzene	10	5	1	15	57
ethyne	22	6	2	12	60
isoprene	16	5	1	17	91
C_8–C_{10} n-alkanes	11	8	1	15	61
xylenes	11	8	1	11	57
NMVOC	13	10	1	13	59

Table S6. Median emission rates (g/s) of 48 VOCs during drilling with Gibson, drilling with Neoflo, fracking, coiled tubing, flowback, and production operations. Values in parentheses represent the lower- and upper-bound CIs.

VOC	Drilling with Gibson	Drilling with Neoflo	Fracking	Coiled Tubing	Flowback	Production
ethane	0.11462 (0.06478, 0.19565)	0.33840 (0.23552, 0.46078)	-0.02760 (-0.07723, 0.01326)	0.24960 (-0.04154, 0.70399)	0.02282 (0.00028, 0.10402)	0.02261 (0.01425, 0.03118)
propane	0.08464 (0.04334, 0.15446)	0.40636 (0.30229, 0.52799)	0.00097 (-0.02550, 0.02087)	0.20456 (-0.01456, 0.57445)	0.03540 (0.00886, 0.09706)	0.02781 (0.02100, 0.03652)
i-butane	0.02071 (0.01144, 0.03625)	0.07399 (0.05086, 0.09986)	-0.00419 (-0.01309, 0.00134)	0.04473 (-0.00457, 0.13102)	0.00562 (0.00022, 0.01953)	0.00625 (0.00465, 0.00801)
n-butane	0.05955 (0.03031, 0.10695)	0.26674 (0.19868, 0.34210)	-0.00957 (-0.03449, 0.00490)	0.12827 (-0.01136, 0.35538)	0.01987 (0.00331, 0.05542)	0.01701 (0.01292, 0.02170)
i-pentane	0.02766 (0.01600, 0.04634)	0.10003 (0.07174, 0.13286)	-0.00494 (-0.01373, 0.00061)	0.05187 (-0.00505, 0.14427)	0.00375 (-0.00153, 0.01847)	0.00473 (0.00315, 0.00635)
n-pentane	0.03283 (0.01686, 0.05796)	0.13403 (0.09596, 0.17362)	-0.00339 (-0.01471, 0.00243)	0.06846 (-0.00635, 0.16255)	0.00568 (-0.00036, 0.02247)	0.00627 (0.00457, 0.00809)
n-hexane	0.02325 (0.01232, 0.04074)	0.10386 (0.07643, 0.13332)	-0.00298 (-0.00879, 0.00017)	0.05228 (-0.00020, 0.09979)	0.00516 (0.00057, 0.01733)	0.00248 (0.00177, 0.00314)
n-heptane	0.01577 (0.00852, 0.02630)	0.06259 (0.04206, 0.08540)	-0.00032 (-0.00194, 0.00143)	0.03097 (-0.00141, 0.05739)	0.00482 (0.00150, 0.01112)	0.00069 (0.00038, 0.00098)
n-octane	0.01586 (0.01031, 0.02446)	0.05505 (0.03956, 0.08095)	-0.00121 (-0.00306, 0.00014)	0.02146 (-0.00232, 0.03777)	0.00315 (0.00062, 0.00851)	0.00033 (0.00016, 0.00054)
n-nonane	0.01158 (0.00662, 0.01754)	0.07845 (0.05325, 0.11027)	-0.00158 (-0.00457, -0.00020)	0.00523 (-0.00719, 0.01419)	0.00324 (0.00105, 0.00603)	0.00016 (0.00002, 0.00037)
n-decane	0.01031 (0.00544, 0.01925)	0.16840 (0.12855, 0.22219)	-0.00270 (-0.00823, -0.00022)	0.00312 (-0.00373, 0.00941)	0.00175 (0.00068, 0.00316)	0.00014 (-0.00008, 0.00043)
cyclopentane	0.00294 (0.00146, 0.00530)	0.01126 (0.00821, 0.01505)	-0.00027 (-0.00126, 0.00021)	0.00576 (-0.00026, 0.01243)	0.00078 (0.00014, 0.00183)	0.00041 (0.00029, 0.00052)
cyclohexane	0.01085 (0.00561, 0.01879)	0.03596 (0.02411, 0.04957)	-0.00034 (-0.00212, 0.00120)	0.02065 (-0.00229, 0.04703)	0.00314 (0.00093, 0.00794)	0.00069 (0.00041, 0.00098)
methylcyclohexane	0.01889 (0.01116, 0.03159)	0.06792 (0.04653, 0.09432)	-0.00151 (-0.00410, 0.00042)	0.04183 (-0.00162, 0.08151)	0.00458 (0.00082, 0.01610)	0.00089 (0.00064, 0.00117)
2,3,4-trimethylpentane	0.00028 (0.00005, 0.00052)	0.00041 (0.00011, 0.00087)	-0.00009 (-0.00029, 0.00005)	0.00069 (-0.00035, 0.00770)	0.00002 (-0.00013, 0.00028)	-0.00007 (-0.00014, -0.00001)
2,2,4-trimethylpentane	0.00414 (0.00236, 0.00700)	0.00719 (0.00490, 0.01112)	-0.00057 (-0.00135, -0.00000)	0.00728 (-0.00142, 0.01425)	0.00102 (0.00007, 0.00238)	0.00015 (-0.00005, 0.00033)
2,3-dimethylpentane	0.00519 (0.00285, 0.00941)	0.02111 (0.01419, 0.02879)	0.00013 (-0.00066, 0.00117)	0.01133 (-0.00080, 0.02197)	0.00141 (0.00041, 0.00476)	0.00024 (0.00004, 0.00045)
2,4-dimethylpentane	0.00101 (0.00059, 0.00179)	0.00413 (0.00295, 0.00568)	0.00004 (-0.00037, 0.00040)	0.00225 (-0.00016, 0.00524)	0.00041 (0.00017, 0.00096)	0.00009 (0.00000, 0.00018)
2-methylhexane	0.00209 (0.00119, 0.00375)	0.00544 (0.00304, 0.00827)	-0.00008 (-0.00059, 0.00040)	0.00332 (-0.00084, 0.00677)	0.00035 (-0.00023, 0.00144)	0.00009 (-0.00003, 0.00024)
3-methylhexane	0.00433 (0.00190, 0.00819)	0.02036 (0.01424, 0.03017)	-0.00036 (-0.00121, 0.00032)	0.01081 (-0.00105, 0.02587)	0.00151 (0.00034, 0.00343)	0.00017 (-0.00001, 0.00037)
2-methylheptane	0.00651 (0.00393, 0.01048)	0.02170 (0.01509, 0.03200)	-0.00069 (-0.00154, -0.00006)	0.01100 (-0.00055, 0.01871)	0.00150 (0.00011, 0.00368)	0.00022 (0.00009, 0.00034)

3-methylheptane	0.00486 (0.00302, 0.00730)	0.01321 (0.00961, 0.01958)	-0.00059 (-0.00112, -0.00012)	0.00524 (-0.00147, 0.00980)	0.00144 (0.00049, 0.00258)	0.00014 (0.00002, 0.00027)

Table S6 (Continued).

VOC	Drilling with Gibson	Drilling with Neoflo	Fracking	Coiled Tubing	Flowback	Production
ethene	0.00083 (0.00006, 0.00174)	-0.00117 (-0.00284, 0.00048)	0.00348 (0.00030, 0.03664)	0.00060 (-0.00282, 0.00256)	0.00016 (-0.00087, 0.00316)	0.00001 (-0.00052, 0.00060)
propene	0.00079 (0.00030, 0.00131)	0.00117 (0.00059, 0.00198)	0.00027 (-0.00054, 0.00205)	0.00043 (-0.00004, 0.00116)	-0.00009 (-0.00045, 0.00032)	-0.00005 (-0.00022, 0.00010)
t-2-butene	0.00002 (-0.00006, 0.00009)	0.00007 (-0.00011, 0.00022)	-0.00018 (-0.00035, -0.00007)	-0.00006 (-0.00024, 0.00010)	-0.00003 (-0.00016, 0.00008)	-0.00004 (-0.00008, -0.00001)
1-butene	0.00006 (-0.00008, 0.00036)	0.00043 (0.00019, 0.00075)	-0.00003 (-0.00034, 0.00025)	-0.00002 (-0.00021, 0.00050)	0.00003 (-0.00016, 0.00017)	-0.00000 (-0.00005, 0.00005)
c-2-butene	0.00007 (-0.00004, 0.00019)	0.00008 (-0.00005, 0.00019)	-0.00007 (-0.00025, 0.00004)	-0.00006 (-0.00026, 0.00007)	-0.00004 (-0.00018, 0.00005)	-0.00005 (-0.00008, -0.00002)
t-2-pentene	0.00002 (-0.00003, 0.00005)	-0.00004 (-0.00011, 0.00005)	-0.00007 (-0.00013, 0.00001)	-0.00002 (-0.00036, 0.00011)	-0.00000 (-0.00004, 0.00005)	-0.00001 (-0.00003, 0.00000)
1-pentene	0.00000 (-0.00009, 0.00008)	0.00009 (-0.00003, 0.00026)	-0.00005 (-0.00022, 0.00017)	-0.00008 (-0.00056, 0.00006)	-0.00011 (-0.00024, -0.00002)	-0.00002 (-0.00005, 0.00002)
c-2-pentene	0.00001 (-0.00002, 0.00009)	-0.00004 (-0.00015, 0.00004)	-0.00003 (-0.00008, 0.00000)	-0.00004 (-0.00013, 0.00001)	-0.00001 (-0.00004, 0.00003)	-0.00000 (-0.00002, 0.00001)
benzene	0.00404 (0.00219, 0.00699)	0.01005 (0.00584, 0.01524)	0.00021 (-0.00068, 0.00216)	0.00680 (-0.00016, 0.01286)	0.00126 (0.00006, 0.00315)	0.00045 (0.00011, 0.00085)
toluene	0.01201 (0.00730, 0.01926)	0.03345 (0.02272, 0.04734)	-0.00121 (-0.00354, 0.00022)	0.02115 (-0.00107, 0.03505)	0.00094 (-0.00296, 0.00579)	-0.00003 (-0.00072, 0.00061)
ethylbenzene	0.00204 (0.00127, 0.00318)	0.00357 (0.00199, 0.00574)	-0.00021 (-0.00071, 0.00009)	0.00187 (-0.00082, 0.00425)	0.00017 (-0.00018, 0.00078)	-0.00007 (-0.00022, 0.00005)
m-p-xylene	0.01074 (0.00686, 0.01638)	0.02079 (0.01202, 0.03232)	-0.00124 (-0.00254, -0.00035)	0.01871 (-0.00102, 0.03046)	0.00330 (0.00113, 0.00684)	-0.00027 (-0.00070, 0.00012)
o-xylene	0.00374 (0.00227, 0.00629)	0.01136 (0.00741, 0.01564)	-0.00038 (-0.00082, -0.00000)	0.00303 (-0.00145, 0.00635)	0.00059 (-0.00002, 0.00189)	-0.00011 (-0.00026, 0.00004)
styrene	0.00019 (0.00005, 0.00045)	0.00037 (0.00018, 0.00063)	0.00001 (-0.00013, 0.00016)	0.00011 (-0.00017, 0.00029)	-0.00005 (-0.00016, 0.00004)	-0.00002 (-0.00005, 0.00000)
i-propylbenzene	0.00104 (0.00052, 0.00189)	0.00295 (0.00119, 0.00592)	-0.00027 (-0.00062, -0.00008)	0.00008 (-0.00041, 0.00111)	0.00003 (-0.00016, 0.00024)	-0.00002 (-0.00004, -0.00001)
n-propylbenzene	0.00122 (0.00066, 0.00192)	0.00105 (0.00053, 0.00168)	-0.00012 (-0.00032, 0.00003)	0.00027 (-0.00030, 0.00073)	0.00011 (-0.00005, 0.00034)	-0.00003 (-0.00007, 0.00001)
3-ethyltoluene	0.00399 (0.00230, 0.00632)	0.00382 (0.00218, 0.00587)	-0.00012 (-0.00041, 0.00013)	0.00059 (-0.00100, 0.00196)	0.00017 (-0.00006, 0.00058)	-0.00008 (-0.00016, -0.00001)
4-ethyltoluene	0.00092 (0.00051, 0.00152)	0.00116 (0.00060, 0.00203)	-0.00000 (-0.00011, 0.00009)	0.00021 (-0.00041, 0.00075)	0.00008 (-0.00004, 0.00024)	-0.00002 (-0.00006, 0.00001)
2-ethyltoluene	0.00142 (0.00064, 0.00238)	0.00101 (0.00056, 0.00169)	-0.00005 (-0.00018, 0.00009)	0.00007 (-0.00038, 0.00050)	0.00001 (-0.00009, 0.00022)	-0.00003 (-0.00007, -0.00000)
1,3,5-trimethylbenzene	0.00118 (0.00051, 0.00265)	0.00251 (0.00149, 0.00376)	-0.00006 (-0.00021, 0.00007)	0.00076 (-0.00020, 0.00188)	0.00012 (-0.00001, 0.00035)	-0.00003 (-0.00006, 0.00000)
1,2,4-trimethylbenzene	0.00995 (0.00097, 0.02428)	0.00594 (0.00342, 0.00986)	-0.00013 (-0.00074, 0.00035)	0.00301 (-0.00101, 0.00623)	0.00036 (-0.00010, 0.00104)	-0.00007 (-0.00020, 0.00005)
1,2,3-trimethylbenzene	0.00214 (0.00106, 0.00359)	0.00157 (0.00087, 0.00264)	-0.00008 (-0.00029, 0.00009)	0.00046 (-0.00029, 0.00110)	0.00003 (-0.00010, 0.00014)	-0.00002 (-0.00006, 0.00001)
1,3-diethylbenzene	0.00028 (0.00013, 0.00051)	0.00029 (0.00014, 0.00051)	-0.00001 (-0.00008, 0.00006)	-0.00005 (-0.00013, 0.00007)	-0.00000 (-0.00005, 0.00004)	-0.00000 (-0.00001, 0.00001)

1,4-diethylbenzene	0.00149 (0.00060, 0.00318)	0.00071 (0.00029, 0.00123)	-0.00008 (-0.00021, 0.00002)	0.00011 (-0.00019, 0.00035)	0.00002 (-0.00007, 0.00010)	-0.00003 (-0.00005, -0.00000)

Table S6 (Continued).

VOC	Drilling with Gibson	Drilling with Neoflo	Fracking	Coiled Tubing	Flowback	Production
ethyne	0.00011 (-0.00023, 0.00060)	-0.00046 (-0.00160, 0.00017)	0.03057 (0.00501, 0.10338)	-0.00006 (-0.00222, 0.00168)	0.00038 (-0.00017, 0.00183)	0.00011 (-0.00013, 0.00050)
isoprene	-0.00016 (-0.00049, -0.00001)	-0.00013 (-0.00060, 0.00007)	-0.00008 (-0.00094, 0.00004)	0.00001 (-0.00184, 0.00016)	-0.00011 (-0.00051, 0.00005)	-0.00013 (-0.00026, -0.00006)

Table S7. Mean emission rates (g/s) of 48 VOCs during drilling with Gibson, drilling with Neoflo, fracking, coiled tubing, flowback, and production operations. Values in parentheses represent the lower- and upper-bound CIs.

VOC	Drilling with Gibson	Drilling with Neoflo	Fracking	Coiled Tubing	Flowback	Production
ethane	0.13310 (0.08040, 0.20654)	0.37822 (0.27338, 0.49632)	0.04141 (-0.01529, 0.10453)	0.34803 (0.07623, 0.69728)	0.06516 (0.02548, 0.11617)	0.02752 (0.01689, 0.03897)
propane	0.11488 (0.07054, 0.17695)	0.42878 (0.32772, 0.55887)	0.01376 (-0.05118, 0.06545)	0.28991 (0.06854, 0.60105)	0.06381 (0.02643, 0.11348)	0.03912 (0.02980, 0.04925)
i-butane	0.02511 (0.01537, 0.03886)	0.07850 (0.05638, 0.10507)	-0.00822 (-0.02747, 0.00325)	0.05684 (0.00707, 0.12795)	0.01192 (0.00308, 0.02219)	0.00757 (0.00551, 0.00977)
n-butane	0.07654 (0.04711, 0.11681)	0.25840 (0.19449, 0.33510)	-0.01082 (-0.06428, 0.02268)	0.16825 (0.03289, 0.36059)	0.03860 (0.01465, 0.07011)	0.02030 (0.01450, 0.02628)
i-pentane	0.03069 (0.01868, 0.04740)	0.09963 (0.07423, 0.13015)	-0.00465 (-0.01857, 0.00816)	0.06259 (0.00916, 0.13762)	0.01328 (0.00329, 0.02641)	0.00447 (0.00235, 0.00653)
n-pentane	0.04095 (0.02517, 0.06197)	0.14215 (0.10661, 0.18330)	-0.00641 (-0.02872, 0.01092)	0.07606 (0.01427, 0.15716)	0.01862 (0.00632, 0.03579)	0.00674 (0.00433, 0.00918)
n-hexane	0.03000 (0.01896, 0.04472)	0.10736 (0.07984, 0.14080)	-0.00694 (-0.02247, 0.00445)	0.04793 (0.01078, 0.09221)	0.01408 (0.00492, 0.02698)	0.00280 (0.00172, 0.00392)
n-heptane	0.02113 (0.01397, 0.03111)	0.07668 (0.05594, 0.10161)	-0.00126 (-0.00799, 0.00333)	0.02727 (0.00522, 0.05236)	0.00916 (0.00269, 0.01903)	0.00097 (0.00039, 0.00161)
n-octane	0.01848 (0.01232, 0.02762)	0.08059 (0.05980, 0.10936)	-0.00110 (-0.00524, 0.00174)	0.01800 (0.00304, 0.03449)	0.00587 (0.00238, 0.01014)	0.00051 (0.00013, 0.00091)
n-nonane	0.01252 (0.00758, 0.01980)	0.12578 (0.09092, 0.17663)	-0.00305 (-0.00875, 0.00030)	0.00677 (-0.00197, 0.01636)	0.00362 (0.00151, 0.00611)	0.00026 (-0.00009, 0.00058)
n-decane	0.01426 (0.00722, 0.02657)	0.23511 (0.17199, 0.32811)	-0.00721 (-0.01841, -0.00022)	-0.00219 (-0.01463, 0.00639)	0.00186 (0.00017, 0.00381)	0.00053 (0.00009, 0.00100)
cyclopentane	0.00387 (0.00238, 0.00575)	0.01170 (0.00866, 0.01530)	-0.00056 (-0.00259, 0.00098)	0.00581 (0.00115, 0.01170)	0.00159 (0.00055, 0.00306)	0.00043 (0.00026, 0.00061)
cyclohexane	0.01290 (0.00738, 0.01987)	0.04509 (0.03313, 0.06015)	-0.00078 (-0.00566, 0.00279)	0.02047 (0.00249, 0.04458)	0.00654 (0.00230, 0.01246)	0.00063 (0.00016, 0.00110)
methylcyclohexane	0.02408 (0.01493, 0.03701)	0.08030 (0.05738, 0.10968)	-0.00192 (-0.00886, 0.00290)	0.03781 (0.00761, 0.07295)	0.01328 (0.00500, 0.02551)	0.00101 (0.00059, 0.00142)
2,3,4-trimethylpentane	0.00030 (0.00011, 0.00054)	0.00063 (0.00016, 0.00113)	-0.00007 (-0.00032, 0.00019)	0.00283 (-0.00007, 0.00964)	0.00026 (-0.00012, 0.00075)	-0.00011 (-0.00024, -0.00001)
2,2,4-trimethylpentane	0.00519 (0.00331, 0.00784)	0.01053 (0.00703, 0.01522)	-0.00057 (-0.00207, 0.00060)	0.00621 (0.00054, 0.01303)	0.00143 (0.00009, 0.00323)	-0.00010 (-0.00043, 0.00020)
2,3-dimethylpentane	0.00706 (0.00421, 0.01075)	0.02316 (0.01686, 0.03083)	-0.00007 (-0.00348, 0.00214)	0.01009 (0.00154, 0.01995)	0.00345 (0.00093, 0.00673)	0.00026 (-0.00005, 0.00052)
2,4-dimethylpentane	0.00130 (0.00079, 0.00207)	0.00409 (0.00297, 0.00546)	0.00022 (-0.00068, 0.00101)	0.00241 (0.00047, 0.00496)	0.00089 (0.00036, 0.00165)	0.00006 (-0.00007, 0.00019)
2-methylhexane	0.00246 (0.00136, 0.00386)	0.00655 (0.00428, 0.00914)	0.00012 (-0.00077, 0.00100)	0.00295 (0.00006, 0.00620)	0.00090 (-0.00000, 0.00207)	0.00020 (-0.00002, 0.00043)
3-methylhexane	0.00556 (0.00211, 0.00921)	0.02713 (0.01912, 0.03714)	-0.00054 (-0.00341, 0.00120)	0.01138 (0.00183, 0.02327)	0.00235 (0.00043, 0.00496)	0.00027 (-0.00001, 0.00053)
2-methylheptane	0.00837 (0.00557, 0.01235)	0.03063 (0.02251, 0.04173)	-0.00055 (-0.00252, 0.00097)	0.00919 (0.00191, 0.01729)	0.00263 (0.00103, 0.00488)	0.00030 (0.00007, 0.00052)

3-methylheptane	0.00473 (0.00299, 0.00695)	0.01787 (0.01340, 0.02339)	-0.00051 (-0.00158, 0.00037)	0.00511 (0.00096, 0.00995)	0.00242 (0.00107, 0.00462)	0.00021 (-0.00003, 0.00045)

Table S7 (Continued).

VOC	Drilling with Gibson	Drilling with Neoflo	Fracking	Coiled Tubing	Flowback	Production
ethene	0.00076 (-0.00006, 0.00169)	-0.00089 (-0.00411, 0.00172)	0.02018 (0.00510, 0.04115)	0.00035 (-0.00279, 0.00358)	0.00301 (0.00008, 0.00734)	0.00074 (-0.00039, 0.00188)
propene	0.00088 (0.00034, 0.00170)	0.00109 (0.00015, 0.00206)	0.00132 (-0.00044, 0.00346)	0.00050 (-0.00000, 0.00119)	0.00003 (-0.00038, 0.00041)	0.00002 (-0.00024, 0.00031)
t-2-butene	0.00002 (-0.00007, 0.00013)	0.00022 (0.00001, 0.00044)	-0.00022 (-0.00050, -0.00006)	-0.00006 (-0.00033, 0.00009)	-0.00007 (-0.00022, 0.00005)	-0.00002 (-0.00008, 0.00003)
1-butene	0.00014 (-0.00004, 0.00037)	0.00067 (0.00034, 0.00106)	-0.00007 (-0.00045, 0.00027)	0.00006 (-0.00028, 0.00053)	-0.00004 (-0.00022, 0.00013)	0.00004 (-0.00006, 0.00014)
c-2-butene	0.00006 (-0.00006, 0.00018)	0.00005 (-0.00020, 0.00024)	-0.00010 (-0.00030, 0.00007)	-0.00010 (-0.00037, 0.00007)	-0.00016 (-0.00042, 0.00001)	-0.00003 (-0.00009, 0.00001)
t-2-pentene	0.00002 (-0.00003, 0.00006)	-0.00002 (-0.00019, 0.00013)	-0.00006 (-0.00016, 0.00002)	-0.00007 (-0.00045, 0.00009)	-0.00001 (-0.00010, 0.00006)	-0.00002 (-0.00005, 0.00001)
1-pentene	-0.00000 (-0.00009, 0.00008)	0.00019 (0.00001, 0.00038)	-0.00002 (-0.00024, 0.00017)	-0.00020 (-0.00117, 0.00009)	-0.00014 (-0.00031, -0.00002)	-0.00004 (-0.00008, 0.00001)
c-2-pentene	0.00003 (-0.00002, 0.00011)	-0.00006 (-0.00024, 0.00007)	-0.00005 (-0.00013, -0.00000)	-0.00009 (-0.00043, 0.00001)	0.00000 (-0.00004, 0.00005)	-0.00002 (-0.00004, 0.00001)
benzene	0.00473 (0.00283, 0.00723)	0.01201 (0.00842, 0.01608)	0.00092 (-0.00113, 0.00278)	0.00605 (0.00114, 0.01165)	0.00170 (0.00022, 0.00349)	0.00098 (0.00040, 0.00155)
toluene	0.01363 (0.00891, 0.02013)	0.03934 (0.02782, 0.05333)	-0.00184 (-0.00620, 0.00118)	0.01691 (0.00285, 0.03218)	0.00275 (-0.00413, 0.01071)	-0.00009 (-0.00135, 0.00109)
ethylbenzene	0.00223 (0.00145, 0.00328)	0.00535 (0.00351, 0.00755)	-0.00043 (-0.00097, -0.00001)	0.00180 (-0.00021, 0.00423)	0.00048 (-0.00024, 0.00135)	-0.00019 (-0.00044, 0.00002)
m-p-xylene	0.01257 (0.00806, 0.01942)	0.02922 (0.01933, 0.04130)	-0.00104 (-0.00306, 0.00066)	0.01411 (0.00205, 0.02724)	0.00514 (0.00211, 0.01026)	-0.00106 (-0.00192, -0.00033)
o-xylene	0.00467 (0.00291, 0.00714)	0.01181 (0.00847, 0.01566)	-0.00032 (-0.00115, 0.00035)	0.00345 (0.00047, 0.00675)	0.00078 (-0.00017, 0.00198)	-0.00022 (-0.00046, 0.00001)
styrene	0.00019 (0.00005, 0.00045)	0.00069 (0.00034, 0.00124)	-0.00007 (-0.00042, 0.00019)	0.00005 (-0.00015, 0.00024)	-0.00006 (-0.00018, 0.00003)	-0.00002 (-0.00006, 0.00003)
i-propylbenzene	0.00140 (0.00066, 0.00253)	0.00613 (0.00392, 0.00904)	-0.00040 (-0.00094, -0.00006)	0.00030 (-0.00037, 0.00117)	0.00012 (-0.00024, 0.00061)	-0.00004 (-0.00007, -0.00001)
n-propylbenzene	0.00128 (0.00079, 0.00190)	0.00141 (0.00085, 0.00207)	-0.00015 (-0.00040, 0.00002)	0.00032 (-0.00009, 0.00077)	0.00029 (0.00001, 0.00072)	-0.00004 (-0.00009, 0.00002)
3-ethyltoluene	0.00505 (0.00286, 0.00910)	0.00450 (0.00253, 0.00712)	-0.00015 (-0.00049, 0.00016)	0.00037 (-0.00137, 0.00233)	0.00040 (-0.00011, 0.00106)	-0.00018 (-0.00032, -0.00006)
4-ethyltoluene	0.00106 (0.00062, 0.00175)	0.00148 (0.00079, 0.00231)	-0.00001 (-0.00018, 0.00014)	-0.00008 (-0.00102, 0.00051)	0.00005 (-0.00016, 0.00028)	-0.00007 (-0.00014, -0.00001)
2-ethyltoluene	0.00160 (0.00086, 0.00275)	0.00113 (0.00053, 0.00179)	-0.00001 (-0.00020, 0.00019)	0.00010 (-0.00028, 0.00048)	0.00008 (-0.00015, 0.00032)	-0.00006 (-0.00011, -0.00001)
1,3,5-trimethylbenzene	0.00190 (0.00063, 0.00476)	0.00303 (0.00193, 0.00461)	-0.00009 (-0.00033, 0.00009)	0.00064 (-0.00125, 0.00194)	0.00036 (0.00012, 0.00079)	-0.00006 (-0.00012, 0.00000)
1,2,4-trimethylbenzene	0.01123 (0.00084, 0.02569)	0.00772 (0.00445, 0.01216)	-0.00031 (-0.00147, 0.00047)	0.00294 (0.00006, 0.00686)	0.00079 (0.00002, 0.00193)	-0.00022 (-0.00045, -0.00002)
1,2,3-trimethylbenzene	0.00268 (0.00139, 0.00474)	0.00192 (0.00108, 0.00302)	-0.00013 (-0.00045, 0.00014)	0.00004 (-0.00157, 0.00144)	0.00009 (-0.00015, 0.00039)	-0.00005 (-0.00011, 0.00001)
1,3-diethylbenzene	0.00026 (0.00011, 0.00046)	0.00041 (0.00024, 0.00062)	-0.00001 (-0.00011, 0.00007)	-0.00002 (-0.00011, 0.00007)	-0.00001 (-0.00009, 0.00005)	0.00000 (-0.00002, 0.00003)

1,4-diethylbenzene	0.00149 (0.00059, 0.00322)	0.00091 (0.00053, 0.00136)	-0.00009 (-0.00033, 0.00010)	0.00014 (-0.00008, 0.00037)	0.00001 (-0.00009, 0.00010)	-0.00004 (-0.00009, -0.00000)

Table S7 (Continued).

VOC	Drilling with Gibson	Drilling with Neoflo	Fracking	Coiled Tubing	Flowback	Production
ethyne	0.00016 (-0.00022, 0.00057)	-0.00070 (-0.00267, 0.00083)	0.05030 (0.01457, 0.09843)	-0.00035 (-0.00518, 0.00367)	0.00238 (0.00015, 0.00594)	0.00365 (0.00202, 0.00548)
isoprene	-0.00031 (-0.00137, 0.00025)	-0.00056 (-0.00165, 0.00016)	-0.00064 (-0.00148, -0.00000)	-0.00059 (-0.00276, 0.00020)	-0.00015 (-0.00046, 0.00010)	-0.00056 (-0.00087, -0.00031)

Table S8. Statistical test results for changes in drilling with Neoflo emission rates before and after 2020 gap for 48 VOC species and 3 VOC groups.

	Number before	Number after	Mean change (g/s)	Median change (g/s)	Median LB Unc (g/s)	p (t-test)	p (U-test)	Trend (t-test)	Trend (U-test)
**ethane**	21	10	0.0542	0.0542	0.1979	0.6124	0.5401	no trend	no trend
**propane**	23	10	0.1063	0.1215	0.2281	0.3802	0.2172	no trend	no trend
**i-butane**	24	10	0.0167	0.0310	0.0577	0.3975	0.2193	no trend	no trend
**n-butane**	24	10	0.0425	0.0413	0.1750	0.5025	0.3544	no trend	no trend
**i-pentane**	24	10	0.0250	0.0349	0.0722	0.3221	0.2338	no trend	no trend
**n-pentane**	24	10	0.0342	0.0347	0.0958	0.3615	0.3351	no trend	no trend
**n-hexane**	25	10	0.0129	-0.0072	0.0716	0.6630	0.6220	no trend	no trend
**n-heptane**	25	10	0.0079	0.0228	0.0497	0.7018	0.4993	no trend	no trend
**n-octane**	23	10	-0.0121	0.0080	0.0485	0.5913	0.8601	no trend	no trend
**n-nonane**	25	10	-0.0730	-0.0308	0.0567	0.0446	0.0766	decrease	no trend
**n-decane**	24	10	-0.0934	-0.0237	0.1242	0.2043	0.5329	no trend	no trend
**cyclopentane**	25	10	0.0036	0.0030	0.0081	0.2692	0.2212	no trend	no trend
**cyclohexane**	25	10	0.0009	0.0020	0.0270	0.9423	0.6220	no trend	no trend
**methylcyclohexane**	23	10	0.0099	0.0252	0.0523	0.6167	0.3573	no trend	no trend
**2,3,4-trimethylpentane**	19	9	0.0005	0.0006	0.0015	0.2636	0.3016	no trend	no trend
**2,2,4-trimethylpentane**	25	10	-0.0029	0.0000	0.0087	0.2802	0.8983	no trend	no trend
**2,3-dimethylpentane**	25	10	0.0043	0.0061	0.0173	0.5409	0.4324	no trend	no trend
**2,4-dimethylpentane**	24	10	0.0008	0.0010	0.0032	0.4921	0.4384	no trend	no trend
**2-methylhexane**	25	10	0.0011	0.0025	0.0063	0.7020	0.5228	no trend	no trend
**3-methylhexane**	25	10	-0.0066	-0.0023	0.0146	0.3952	0.6220	no trend	no trend
**2-methylheptane**	24	10	0.0001	0.0038	0.0170	0.9871	0.5083	no trend	no trend
**3-methylheptane**	22	10	-0.0005	0.0008	0.0102	0.9225	0.7604	no trend	no trend
**ethene**	19	10	0.0040	0.0028	0.0098	0.0715	0.0459	no trend	increase
**propene**	22	10	0.0018	0.0010	0.0027	0.0727	0.0704	no trend	no trend
**t-2-butene**	19	9	0.0001	-0.0001	0.0006	0.5484	0.8440	no trend	no trend
**1-butene**	20	10	0.0001	0.0001	0.0010	0.8577	0.5824	no trend	no trend
**c-2-butene**	20	7	0.0000	0.0000	0.0006	0.9870	0.7253	no trend	no trend
**t-2-pentene**	19	9	-0.0002	-0.0001	0.0005	0.0748	0.1155	no trend	no trend
**1-pentene**	19	9	-0.0002	-0.0002	0.0005	0.1027	0.2009	no trend	no trend
**c-2-pentene**	20	9	-0.0001	0.0001	0.0004	0.5192	0.9812	no trend	no trend
**benzene**	24	10	0.0038	0.0059	0.0099	0.3476	0.3951	no trend	no trend
**toluene**	23	10	0.0094	0.0176	0.0315	0.4210	0.4219	no trend	no trend
**ethylbenzene**	22	10	0.0002	0.0032	0.0034	0.8923	0.4520	no trend	no trend
**styrene**	14	10	-0.0001	0.0003	0.0006	0.7210	0.2300	no trend	no trend
**m+p-xylene**	23	10	0.0113	0.0268	0.0204	0.2400	0.0884	no trend	no trend
**o-xylene**	24	10	0.0071	0.0104	0.0083	0.0599	0.0472	no trend	increase
**i-propylbenzene**	24	10	-0.0067	-0.0039	0.0042	0.0000	0.0000	decrease	decrease
**n-propylbenzene**	23	10	0.0012	0.0015	0.0014	0.0297	0.0178	increase	increase

Table S8 (Continued).

**3-ethyltoluene**	18	10	0.0017	0.0031	0.0035	0.3388	0.1310	no trend	no trend
**4-ethyltoluene**	18	10	0.0009	0.0016	0.0014	0.1518	0.0649	no trend	no trend
**1,3,5-trimethylbenzene**	18	10	0.0016	0.0029	0.0018	0.1091	0.0522	no trend	no trend
**2-ethyltoluene**	20	10	0.0013	0.0014	0.0013	0.0035	0.0052	increase	increase
**1,2,4-trimethylbenzene**	18	10	0.0038	0.0070	0.0068	0.1889	0.1192	no trend	no trend
**1,2,3-trimethylbenzene**	18	10	0.0011	0.0017	0.0020	0.1443	0.1192	no trend	no trend
**1,3-diethylbenzene**	21	10	0.0002	0.0004	0.0004	0.2075	0.1130	no trend	no trend
**1,4-diethylbenzene**	16	10	0.0007	0.0011	0.0011	0.0571	0.0328	no trend	increase
**ethyne**	15	8	0.0020	0.0008	0.0050	0.1005	0.1006	no trend	no trend
**isoprene**	21	8	0.0006	0.0004	0.0023	0.1311	0.3242	no trend	no trend
**C_8–C_{10} n-alkanes**	24	10	-0.2358	-0.0853	0.2310	0.0817	0.2193	no trend	no trend
**xylenes**	24	10	0.0156	0.0362	0.0316	0.2371	0.1354	no trend	no trend
**NMVOC**	22	10	0.1901	0.3056	1.5379	0.7552	0.6695	no trend	no trend

Table S9. Statistical test results for changes in flowback emission rates before and after 2020 gap for 48 VOC species and 3 VOC groups.

	Number before	Number after	Mean change (g/s)	Median change (g/s)	Median LB Unc (g/s)	p (t-test)	p (U-test)	Trend (t-test)	Trend (U-test)
**ethane**	9	5	-0.0126	-0.0118	0.0906	0.7997	0.8981	no trend	no trend
**propane**	11	5	0.0023	0.0027	0.0629	0.9646	0.6612	no trend	no trend
**i-butane**	11	6	0.0020	0.0036	0.0141	0.8146	0.5249	no trend	no trend
**n-butane**	11	6	-0.0021	0.0092	0.0408	0.9383	0.6605	no trend	no trend
**i-pentane**	10	6	-0.0052	-0.0059	0.0247	0.6377	0.8749	no trend	no trend
**n-pentane**	12	6	-0.0091	-0.0102	0.0225	0.5213	0.6820	no trend	no trend
**n-hexane**	12	6	-0.0132	-0.0104	0.0097	0.1721	0.1505	no trend	no trend
**n-heptane**	12	6	-0.0126	-0.0061	0.0079	0.0711	0.0245	no trend	decrease
**n-octane**	10	5	-0.0056	-0.0061	0.0039	0.0497	0.0553	decrease	no trend
**n-nonane**	10	5	-0.0029	-0.0031	0.0034	0.0919	0.0992	no trend	no trend
**n-decane**	9	4	-0.0030	-0.0025	0.0035	0.0453	0.0112	decrease	decrease
**cyclopentane**	12	6	-0.0013	-0.0009	0.0015	0.1911	0.2496	no trend	no trend
**cyclohexane**	12	5	-0.0046	-0.0031	0.0054	0.3190	0.1296	no trend	no trend
**methylcyclohexane**	11	5	-0.0142	-0.0069	0.0072	0.0993	0.0517	no trend	no trend
**2,3,4-trimethylpentane**	11	5	-0.0008	-0.0001	0.0005	0.0901	0.1451	no trend	no trend
**2,2,4-trimethylpentane**	11	6	-0.0032	-0.0023	0.0032	0.0066	0.0019	decrease	decrease
**2,3-dimethylpentane**	12	5	-0.0021	-0.0016	0.0032	0.4476	0.1946	no trend	no trend
**2,4-dimethylpentane**	11	6	-0.0010	-0.0002	0.0014	0.0933	0.3011	no trend	no trend
**2-methylhexane**	12	6	-0.0011	-0.0010	0.0023	0.1625	0.1246	no trend	no trend
**3-methylhexane**	11	6	-0.0035	-0.0021	0.0030	0.0410	0.0616	decrease	no trend
**2-methylheptane**	10	5	-0.0033	-0.0028	0.0017	0.0197	0.0127	decrease	decrease
**3-methylheptane**	11	4	-0.0022	-0.0005	0.0015	0.1177	0.1773	no trend	no trend
**ethene**	10	5	-0.0048	-0.0017	0.0050	0.0730	0.3710	no trend	no trend
**propene**	9	4	-0.0001	0.0001	0.0016	0.7424	0.5035	no trend	no trend
**t-2-butene**	9	5	-0.0003	-0.0003	0.0005	0.0695	0.0829	no trend	no trend
**1-butene**	9	4	-0.0002	-0.0002	0.0007	0.3132	0.3301	no trend	no trend
**c-2-butene**	7	6	-0.0004	-0.0003	0.0004	0.0816	0.0513	no trend	no trend
**t-2-pentene**	10	5	-0.0002	-0.0001	0.0002	0.0838	0.0400	no trend	decrease
**1-pentene**	11	2	0.0002	0.0002	0.0005	0.1171	0.0256	no trend	increase
**c-2-pentene**	9	5	-0.0001	0.0000	0.0001	0.0517	0.1119	no trend	no trend
**benzene**	12	5	-0.0027	-0.0026	0.0059	0.0027	0.0136	decrease	decrease
**toluene**	10	5	-0.0073	-0.0063	0.0147	0.1576	0.0753	no trend	no trend
**ethylbenzene**	12	5	-0.0014	-0.0008	0.0016	0.0410	0.0365	decrease	decrease
**styrene**	10	4	0.0000	0.0000	0.0004	0.7804	1.0000	no trend	no trend
**m+p-xylene**	11	4	-0.0065	-0.0048	0.0045	0.0440	0.0029	decrease	decrease
**o-xylene**	12	6	-0.0018	-0.0015	0.0018	0.0111	0.0182	decrease	decrease
**i-propylbenzene**	12	6	-0.0005	-0.0002	0.0008	0.0859	0.1797	no trend	no trend
**n-propylbenzene**	11	5	-0.0004	-0.0003	0.0007	0.1073	0.1804	no trend	no trend

Table S9 (Continued).

**3-ethyltoluene**	12	5	-0.0011	-0.0003	0.0007	0.0586	0.1296	no trend	no trend
**4-ethyltoluene**	11	5	-0.0004	-0.0002	0.0005	0.0596	0.0192	no trend	decrease
**1,3,5-trimethylbenzene**	11	4	-0.0003	-0.0002	0.0003	0.2619	0.4117	no trend	no trend
**2-ethyltoluene**	11	5	-0.0002	-0.0002	0.0004	0.1877	0.1149	no trend	no trend
**1,2,4-trimethylbenzene**	11	5	-0.0015	-0.0010	0.0012	0.0358	0.0055	decrease	decrease
**1,2,3-trimethylbenzene**	12	5	-0.0003	-0.0001	0.0005	0.1752	0.3284	no trend	no trend
**1,3-diethylbenzene**	12	5	0.0000	0.0000	0.0003	0.9281	0.7214	no trend	no trend
**1,4-diethylbenzene**	9	4	0.0000	0.0000	0.0003	0.6743	0.9399	no trend	no trend
**ethyne**	10	6	-0.0035	-0.0008	0.0026	0.1413	0.4923	no trend	no trend
**isoprene**	9	2	-0.0001	-0.0003	0.0005	0.3848	0.4364	no trend	no trend
**C_8–C_{10} n-alkanes**	9	4	-0.0116	-0.0109	0.0088	0.0211	0.0336	decrease	decrease
**xylenes**	11	6	-0.0111	-0.0068	0.0080	0.0103	0.0006	decrease	decrease
**NMVOC**	10	5	0.5500	-0.2044	0.3040	0.6513	0.5135	no trend	no trend

671 **Table S10.** Mann-Kendall test results for production emission rates at ICB and LS pads.

	Production Emission Rate (ER) Trend at ICB				Production Emission Rate (ER) Trend at LS			
	**Trend**	**p**	**Number of ER**	**Number of ER with LB > 0**	**Trend**	**p**	**Number of ER**	**Number of ER with LB > 0**
**ethane**	no trend	0.095564	57	36	no trend	0.195671	36	18
**propane**	no trend	0.85402	61	48	no trend	0.30259	38	30
**i_butane**	no trend	0.633922	58	41	no trend	0.375964	36	24
**n_butane**	no trend	0.770117	58	42	no trend	0.470351	36	26
**i_pentane**	increasing	0.04514	51	23	no trend	0.976347	34	17
**n_pentane**	no trend	0.242448	54	30	no trend	0.38989	34	21
**n_hexane**	increasing	0.026505	58	36	no trend	0.763556	37	25
**n_heptane**	increasing	0.044106	57	25	no trend	0.333502	36	16
**octane**	no trend	0.268371	61	31	no trend	0.287091	39	18
**nonane**	no trend	0.356108	55	32	no trend	0.792238	33	17
**decane**	no trend	0.086224	56	31	no trend	0.105455	35	17
**cyclopentane**	no trend	0.072965	51	28	no trend	0.505245	33	19
**cyclohexane**	no trend	0.413985	57	32	no trend	0.66603	37	19
**methylcyclohexane**	increasing	0.042109	55	35	no trend	0.119616	37	24
**2_3_4_trimethylpentane**	no trend	0.916668	43	11	no trend	0.966743	27	5
**2_2_4_trimethylpentane**	no trend	0.500499	55	17	no trend	0.865516	39	12
**2_3_dimethylpentane**	no trend	0.425503	51	20	no trend	0.592551	32	11
**2_4_dimethylpentane**	no trend	0.730844	52	25	no trend	0.338683	32	13
**2_methylhexane**	no trend	0.165175	47	22	no trend	0.943108	30	13
**3_methylhexane**	no trend	0.833437	48	17	no trend	0.453662	30	9
**2_methylheptane**	no trend	0.156244	55	32	increasing	0.024239	34	19
**3_methylheptane**	increasing	0.006141	53	32	increasing	0.026712	33	21
**ethene**	decreasing	0.033895	51	32	no trend	0.252523	29	10
**propene**	no trend	0.557462	46	15	no trend	0.540239	28	6
**t_2_butene**	no trend	0.337204	44	17	no trend	0.540239	28	10
**1_butene**	no trend	0.737237	48	19	no trend	0.341199	31	11
**c_2_butene**	no trend	0.933824	41	15	no trend	0.316995	27	7
**t_2_pentene**	no trend	0.568024	47	16	no trend	0.587805	27	7
**1_pentene**	no trend	0.107679	44	10	increasing	0.033561	25	6
**c_2_pentene**	no trend	0.136942	48	20	no trend	0.110118	31	12
**benzene**	no trend	0.268588	50	28	no trend	0.890007	28	11
**toluene**	increasing	0.012523	47	8	no trend	0.259887	34	4
**ethylbenzene**	no trend	0.481063	47	17	increasing	0.005313	31	11
**styrene**	no trend	0.794117	55	15	increasing	0.031262	33	8
**m_p_xylene**	no trend	0.314154	48	10	no trend	0.864665	33	1
**o_xylene**	no trend	0.595884	47	9	no trend	0.284975	29	4

Table S10 (Continued).

**i_propylbenzene**	no trend	0.070117	57	17	no trend	0.795792	36	10
**n_propylbenzene**	no trend	0.071574	50	13	no trend	0.139689	35	5
**3_ethyltoluene**	no trend	0.610656	53	8	no trend	0.858807	34	2
**4_ethyltoluene**	no trend	0.765061	56	11	no trend	0.776388	35	6
**1_3_5_trimethylbenzene**	no trend	0.587583	52	11	no trend	0.42341	34	4
**2_ethyltoluene**	no trend	0.790519	55	9	no trend	0.440783	34	6
**1_2_4_trimethylbenzene**	no trend	0.714924	53	10	no trend	0.858807	34	4
**1_2_3_trimethylbenzene**	no trend	0.500499	57	12	no trend	0.099326	36	6
**1_3_diethylbenzene**	no trend	0.221536	55	21	no trend	0.545656	33	14
**1_4_diethylbenzene**	no trend	0.381755	55	17	increasing	0.034752	36	11
**ethyne**	decreasing	7.61E-09	54	41	increasing	2E-05	32	19
**isoprene**	no trend	0.286742	39	7	no trend	0.341718	23	5
**C_8–C_{10} n-alkanes**	no trend	0.187799	62	31	no trend	0.953545	40	16
**xylenes**	no trend	0.508179	48	9	no trend	0.757997	32	3
**NMVOC**	no trend	0.183112	60	44	no trend	0.529614	38	24

Table S11. Comparison of lower-bound uncertainties for selected VOCs, contrasting the median uncertainty of individual weekly emission rates (“Weekly”) with the aggregated uncertainty of the median emission rate for each operation (“Aggregated”).

		Drilling with Gibson (g/s)	Drilling with Neoflo (g/s)	Fracking (g/s)	Coil Tubing (g/s)	Flowback (g/s)	Production (g/s)
Ethane	Weekly	0.105	0.207	0.193	0.233	0.091	0.041
	Aggregated	0.05	0.104	0.049	0.291	0.022	0.008
Propane	Weekly	0.094	0.243	0.12	0.191	0.063	0.034
	Aggregated	0.041	0.104	0.026	0.219	0.027	0.007
C_8–C_{10} n-alkanes	Weekly	0.026	0.232	0.045	0.028	0.0088	0.0047
	Aggregated	0.015	0.085	0.009	0.041	0.0071	0.0005
Benzene	Weekly	0.0039	0.0104	0.0053	0.0047	0.0059	0.0023
	Aggregated	0.002	0.004	0.0009	0.007	0.001	0.0003
Toluene	Weekly	0.0091	0.032	0.0112	0.0129	0.0147	0.006
	Aggregated	0.0047	0.0107	0.0023	0.0222	0.0039	0.0007
Ethylbenzene	Weekly	0.00151	0.00377	0.00288	0.00216	0.00156	0.00128
	Aggregated	0.00077	0.00158	0.00051	0.00269	0.00035	0.00014
Xylenes	Weekly	0.01114	0.03449	0.01207	0.01454	0.00801	0.00587
	Aggregated	0.00558	0.01333	0.00167	0.02471	0.00254	0.00059
Ethyne	Weekly	0.00117	0.00501	0.02472	0.00326	0.00261	0.00317
	Aggregated	0.00034	0.00113	0.02556	0.00216	0.00055	0.00024
NMVOC	Weekly	0.477	1.608	0.757	0.781	0.304	0.188
	Aggregated	0.213	0.635	0.15	1.175	0.101	0.028

Table S12. Median emission rates of select VOCs for drilling operations: this study vs. EPA Emission Tool vs. Hecobian et al. (2019). "NA" denotes no available value. Values in paratheses represent the lower- and upper-bound CIs.

**VOC**	**This study drilling Gibson mud median emission rates (g/s)**	**This study drilling Neoflo mud median emission rates (g/s)**	**EPA Emission Tool oil-based or synthetic-based drilling mud degassing emission rate (g/s)**	**Hecobian et al. drilling median emission rate (g/s) -- Piceance basin**
Ethane	0.11462 (0.06478, 0.19565)	0.33840 (0.23552, 0.46078)	NA	0.13
Propane	0.08464 (0.04334, 0.15446)	0.40636 (0.30229, 0.52799)	NA	0.12
C_8–C_{10} n-alkanes	0.03906 (0.02476, 0.05912)	0.30484 (0.21673, 0.41516)	NA	0.007
Benzene	0.00404 (0.00219, 0.00699)	0.01005 (0.00584, 0.01524)	NA	0.004
Toluene	0.01201 (0.00730, 0.01926)	0.03345 (0.02272, 0.04734)	NA	0.088
Ethylbenzene	0.00204 (0.00127, 0.00318)	0.00357 (0.00199, 0.00574)	NA	0.0009
Xylenes	0.01448 (0.00890, 0.02275)	0.03305 (0.01972, 0.04920)	NA	0.004
Ethyne	0.00011 (-0.00023, 0.00060)	-0.00046 (-0.00160, 0.00017)	NA	NA
NMVOC	0.45919 (0.24855, 0.81626)	2.82726 (2.19796, 3.48037)	0.36	0.43

Table S13. Median emission rates of select VOCs for hydraulic fracturing operations: this study (weekly samples) vs. EPA Emission Tool vs. Hecobian et al. (2019). "NA" denotes no available value. Values in paratheses represent the lower- and upper-bound CIs.

VOC	This study Fracking median emission rates (g/s)	EPA Emission Tool fracking 700 hp engine emission rate (g/s)	EPA Emission Tool fracking 1500 hp engine emission rate (g/s)	Hecobian et al. (2019) fracking median emission rate (g/s) -- DJ basin
Ethane	-0.02760 (-0.07723, 0.01326)	NA	NA	0.0026
Propane	0.00097 (-0.02550, 0.02087)	NA	NA	0.0005
C_8–C_{10} n-alkanes	-0.00545 (-0.01449, -0.00049)	NA	NA	0.0105
Benzene	0.00021 (-0.00068, 0.00216)	0.002	0.0042	0.0022
Toluene	-0.00121 (-0.00354, 0.00022)	0.0014	0.003	0.0056
Ethylbenzene	-0.00021 (-0.00071, 0.00009)	0.0002	0.0004	0.0008
Xylenes	-0.00155 (-0.00322, -0.00040)	0.0005	0.0011	0.0056
Ethyne	0.03057 (0.00501, 0.10338)	NA	NA	NA
NMVOC	-0.04073 (-0.19209, 0.10235)	0.04	0.08	0.08

Table S14. Median emission rates of select VOCs for coiled tubing/millout operations. Values in paratheses represent the lower- and upper-bound CIs.

VOC	This study coiled tubing median emission rates (g/s)
Ethane	0.24960 (-0.04154, 0.70399)
Propane	0.20456 (-0.01456, 0.57445)
C_8–C_{10} n-alkanes	0.01796 (-0.02187, 0.04984)
Benzene	0.00680 (-0.00016, 0.01286)
Toluene	0.02115 (-0.00107, 0.03505)
Ethylbenzene	0.00187 (-0.00082, 0.00425)
Xylenes	0.02181 (-0.00291, 0.03537)
Ethyne	-0.00006 (-0.00222, 0.00168)
NMVOC	1.09292 (-0.08707, 2.77241)

Table S15. Median emission rates of select VOCs for flowback operations: this study (weekly samples) vs. EPA Emission Tool vs. Hecobian et al. (2019). "NA" denotes no available value. Values in paratheses represent the lower- and upper-bound CIs.

VOC	This study flowback median emission rates (g/s)	EPA Emission Tool flowback green-completion emission rate (g/s)	Hecobian et al. (2019) flowback median emission rate (g/s) -- DJ basin
Ethane	0.02282 (0.00028, 0.10402)	0	1.1
Propane	0.03540 (0.00886, 0.09706)	0	0.75
C_8–C_{10} n-alkanes	0.01100 (0.00385, 0.01977)	0	0.342
Benzene	0.00126 (0.00006, 0.00315)	0	0.069
Toluene	0.00094 (-0.00296, 0.00579)	0	0.21
Ethylbenzene	0.00017 (-0.00018, 0.00078)	0	0.019
Xylenes	0.00246 (-0.00008, 0.00722)	0	0.274
Ethyne	0.00038 (-0.00017, 0.00183)	0	NA
NMVOC	0.22533 (0.12118, 0.53567)	0	6.33

Table S16. Median emission rates of select VOCs for production operations: this study (weekly samples) vs. Hecobian et al. (2019). "NA" denotes no available value. Values in paratheses represent the lower- and upper-bound CIs.

VOC	This study production median emission rates (g/s)	Hecobian et al. (2019) production median emission rate (g/s) -- DJ basin
Ethane	0.02261 (0.01425, 0.03118)	0.1
Propane	0.02781 (0.02100, 0.03652)	0.088
C_8–C_{10} n-alkanes	0.00048 (-0.00001, 0.00100)	0.0021
Benzene	0.00045 (0.00011, 0.00085)	0.0013
Toluene	-0.00003 (-0.00072, 0.00061)	0.0011
Ethylbenzene	-0.00007 (-0.00022, 0.00005)	0.0002
Xylenes	-0.00038 (-0.00097, 0.00012)	0.0016
Ethyne	0.00011 (-0.00013, 0.00050)	NA
NMVOC	0.11309 (0.08548, 0.14534)	0.33

REFERENCES

1. Ku, I.-T.; Zhou, Y.; Hecobian, A.; Benedict, K.; Buck, B.; Lachenmayer, E.; Terry, B.; Frazier, M.; Zhang, J.; Pan, D.; Low, L.; Sullivan, A.; Collett, J. L. Air Quality Impacts from the Development of Unconventional Oil and Gas Well Pads: Air Toxics and Other Volatile Organic Compounds. Atmospheric Environment 2024, 317, 120187. https://doi.org/10.1016/j.atmosenv.2023.120187.
2. Weber, D.T. Volatile Organic Compound Concentrations and the Impacts of Future Oil and Natural Gas Development in the Colorado Northern Front Range. M.S. Thesis, Colorado State University, Fort Collins, CO, 2018. https://doi.org/10.25675/3.020324.
3. LeBouf, R. F.; Stefaniak, A. B.; Virji, M. A. Validation of Evacuated Canisters for Sampling Volatile Organic Compounds in Healthcare Settings. J. Environ. Monit. 2012, 14 (3), 977–983. https://doi.org/10.1039/C2EM10896H.
4. Brown, P.J., Fuller, W.A., American Mathematical Society, Institute of Mathematical Statistics, Society for Industrial and Applied Mathematics (Eds.), 1990. Statistical analysis of measurement error models and applications: proceedings of the AMS-IMS-SIAM joint summer research conference held June 10-16, 1989, with support from the National Science Foundation and the U.S. Army Research Office, Contemporary mathematics. Presented at the AMS-IMS-SIAM Joint Summer Research Conference in the Mathematical Sciences on Statistical Analysis of Measurement Error Models and Applications, American Mathematical Society, Providence, R.I.

5. Refsgaard, J. C.; van der Sluijs, J. P.; Brown, J.; van der Keur, P. A Framework for Dealing with Uncertainty Due to Model Structure Error. Advances in Water Resources 2006, 29 (11), 1586–1597. https://doi.org/10.1016/j.advwatres.2005.11.013.
6. Kroese, Dirk P., Thomas Taimre, and Zdravko I. Botev. Handbook of Monte Carlo Methods. John Wiley & Sons, 2013. DOI:10.1002/9781118014967
7. ISO., I., and BIPM OIML. Guide to the Expression of Uncertainty in Measurement. Madrid, Spain: Aenor, 1993.
8. Cohen, J. T., Lampson, M. A. and Bowers, T. S. The use of two - stage Monte Carlo simulation techniques to characterize variability and uncertainty in risk analysis. Human and Ecological Risk Assessment: An International Journal, 1996, 2(4), pp. 939 - 971. doi: 10.1080/10807039609383657.
9. Sheskin, D. J. Handbook of Parametric and Nonparametric Statistical Procedures: Third Edition, 3rd ed.; Chapman and Hall/CRC: New York, 2003. https://doi.org/10.1201/9781420036268.
10. Hirsch, R. M.; Slack, J. R.; Smith, R. A. Techniques of Trend Analysis for Monthly Water Quality Data. Water Resources Research 1982, 18 (1), 107–121. https://doi.org/10.1029/WR018i001p00107.
11. CDPHE. Investigation of Reported Health Concerns Near Livingston Oil and Gas Site in Broomfield. 2020.
12. Stull, R.B. (Ed.). An Introduction to Boundary Layer Meteorology. Springer Netherlands, Dordrecht. 1988. https://doi.org/10.1007/978-94-009-3027-8

13. Eastern Research Group, Inc. 2020 Nonpoint Oil and Gas Emission Estimation Tool Version 1.3; 2022. https://www.epa.gov/system/files/documents/2024-02/2020-nonpoint-oil-and-gas-emission-estimation-tool-v1_3.pdf (accessed 2025-11-22)

14. Hecobian, A.; Clements, A. L.; Shonkwiler, K. B.; Zhou, Y.; MacDonald, L. P.; Hilliard, N.; Wells, B. L.; Bibeau, B.; Ham, J. M.; Pierce, J. R.; Collett, J. L. Jr. Air Toxics and Other Volatile Organic Compound Emissions from Unconventional Oil and Gas Development. Environ. Sci. Technol. Lett. 2019, 6 (12), 720–726. https://doi.org/10.1021/acs.estlett.9b00591.
